# Reactivity of highly Lewis acidic diborane(4) towards pyridine and isocyanide: formation of boraalkene–pyridine complex and *ortho*-functionalized pyridine derivatives†
†Electronic supplementary information (ESI) available: Experimental and computational details, and Cartesian coordinate. CCDC 1583725–1583743. For ESI and crystallographic data in CIF or other electronic format see DOI: 10.1039/c7sc04759b


**DOI:** 10.1039/c7sc04759b

**Published:** 2017-12-11

**Authors:** Yuhei Katsuma, Hiroki Asakawa, Makoto Yamashita

**Affiliations:** a Department of Applied Chemistry , Faculty of Science and Engineering , Chuo University , 1-13-27 Kasuga , Bunkyo-ku , 112-8551 , Tokyo , Japan; b Department of Molecular and Macromolecular Chemistry , Graduate School of Engineering , Nagoya University , Furo-cho, Chikusa-ku , Nagoya , 464-8603 , Aichi , Japan . Email: makoto@oec.chembio.nagoya-u.ac.jp

## Abstract

The reaction of pinB-BMes_2_ (pin = pinacolato, Mes = 2,4,6-Me_3_C_6_H_2_) with Xyl-NC (Xyl = 2,6-Me_2_C_6_H_3_) and pyridine results in the formation of a pyridine-coordinated boraalkene that exhibits an intense color caused by an intramolecular charge-transfer interaction. In the presence of an excess of pyridine, the *ortho* C–H bond of pyridine was selectively functionalized to afford a quinoid compound or an isocyanide-coupled product. Based on the concentration effect, the reaction stoichiometry, and previously reported DFT calculations, a reaction mechanism that involves several rearrangement reactions was proposed. Using the present method, substituted pyridines and N-heterocycles afforded the corresponding functionalized derivatives. A subsequent hydrolysis of one of the resulting products furnished an aminomethylated pyridine derivative in two steps from parent pyridine.

## Abstract 2

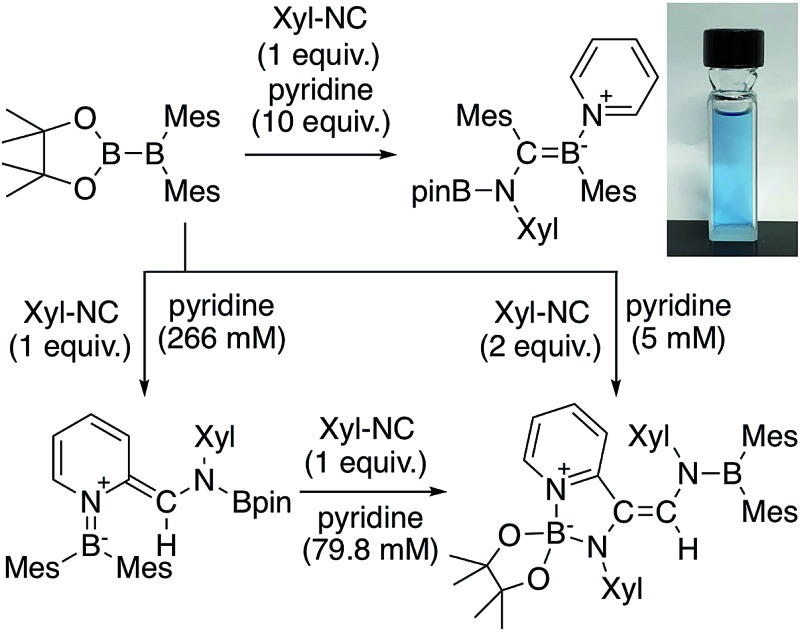
The reaction of pinB-BMes_2_ with Xyl-NC and pyridine results in the formation of a pyridine-coordinated boraalkene that exhibits an intense color. In the presence of an excess of pyridine, the *ortho* C–H bond of pyridine was selectively functionalized.

## Introduction

Pyridine is an important building block in pharmaceutical, materials, and organometallic chemistry. Due to the presence of the nitrogen atom in the pyridine ring, it should be possible to selectively functionalize the C–H bonds at the 2-, 3- and 4-positions of pyridine to construct bespoke molecular skeletons. Although many reports on the selective functionalization of pyridine derivatives can be found in the scientific literature,^1^ transition-metal-catalyzed C–H functionalizations of pyridine have become important, as they represent step- and atom-economical synthetic routes.^2^ Following the very early discovery of a selective functionalization of pyridine with transition-metal-based catalysts,^3^ several other metal-catalyzed functionalizations of pyridine have been reported.^1^ Furthermore, a recently reported “cooperative catalyst” system has demonstrated high selectivity and catalytic activity toward the functionalization of pyridine.^4^ Historically, anionic nucleophiles^5^ or electron-rich radicals^6^ have been used to selectively functionalize the 2-position (“*ortho*”-position) of pyridine. Inspired by these strategies, further new methods based on nucleophilic functionalization^7^ and radical addition^8^ are currently developed. It should be noted that the *ortho*-metalation of pyridine might represent an important method to achieve the selective functionalization of pyridine at the 2-position.^9^

Diborane(4) compounds that contain a B–B single bond^10^ are widely used in organic synthesis, especially for metal-catalyzed borylation reactions.^11^ In contrast to the rich chemistry of metal-catalyzed borylations, direct reactions between diborane(4) compounds and organic compounds remain scarce.^12^ Halogen-substituted diborane(4) compounds can react with alkenes and alkynes in the absence of a catalyst.^13^ In contrast, there have been no reports of oxygen-substituted diborane(4)s undergoing direct reactions with organic molecules until recently. The addition of nucleophilic or basic activators enables diborane(4)s to react with organic molecules.^14^ It should also be noted that diazo compounds derived from tosylhydrazone or similar carbenoid species can react with diborane(4) to form the corresponding alkylboronates in the absence of a metal catalyst.^15^ Independent of these two-electron processes, effective radical activations have been discovered for borylation reactions with diborane(4)s.^16^ More recently, direct reactions of some pyridine derivatives with diborane(4)s *via* ionic or radical pathways have been reported.^17^

**Scheme 1:**
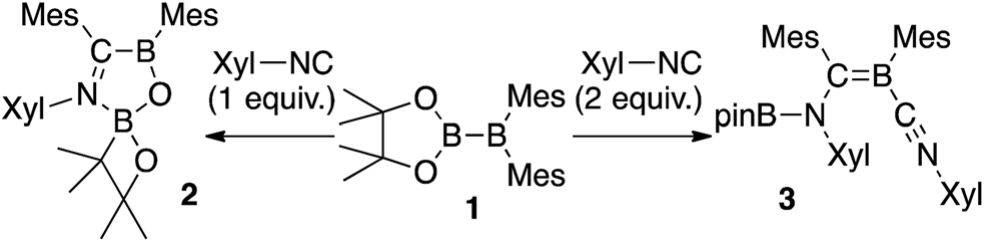
Previously reported reactions of the unsymmetrical diborane(4) **1** with Xyl-NC (Xyl = 2,6-Me_2_C_6_H_3_).

We have recently reported the operationally simple synthesis of unsymmetrical diborane(4) **1**,^18^ its reactivity toward CO and *tert*-butyl isocyanide inducing a cleavage of multiple bond(s),^18^ its high Lewis acidity and one-electron reduction to form a radical anion,^19^ as well as its reactivity toward Xyl-NC (Xyl = 2,6-dimethylphenyl) to form a spirocyclic oxaboretane (**2**) or isocyanide-coordinated boraalkene (**3**)^20^ (Scheme 1). The formation of **2** is a rare example of a ring contraction reaction that affords a four-membered boracycle. DFT calculations showed that several rearrangement reactions are involved in these transformations,^18^,^20^ as Lewis-base-coordinated ligands on the diborane(4) are known to undergo migration.^21^ Herein, we report the selective C–H functionalization of pyridine and other N-heterocycles with **1** and Xyl-NC. The thus obtained products, *i.e.*, pyridine-coordinated boraalkenes, dearomatized *ortho*-quinoid derivatives of pyridine, and *ortho*-functionalized pyridines from a reductive coupling of isocyanide, were fully characterized. Complex reaction mechanisms were postulated based on the structures of the products and previously reported DFT-based mechanisms.^18^,^20^ One of the obtained functionalized pyridines was subsequently hydrolyzed to afford an aminomethylated pyridine derivative.

## Results and discussion

**Scheme 2:**
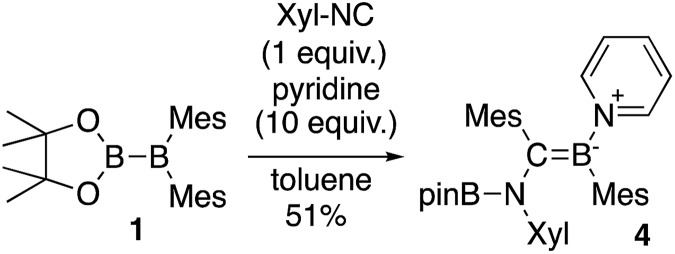
Reaction of **1** with Xyl-NC and 10 equiv. of pyridine (yield estimated by ^1^H NMR spectroscopy).

**Fig. 1:**
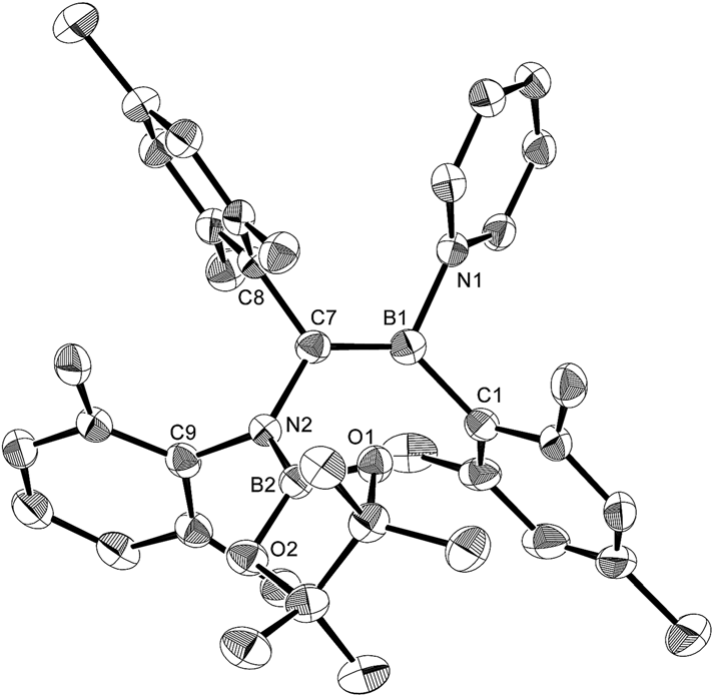
Molecular structure of **4** (thermal ellipsoids set at 50% probability; one of the two independent molecules of **4** per unit cell and hydrogen atoms omitted for clarity). Selected bond distances (Å), angles (°) and dihedral angles (°): B1–N1 1.586(3), B1–C1 1.594(3), B1–C7 1.441(3), C7–N2 1.482(3), N2–B2 1.404(3), N2–C9 1.454(3), C7–C8 1.501(3); N1–B1–C1 110.09(18), N1–B1–C7 115.8(2), C1–B1–C7 134.1(2), B1–C7–N2 123.1(2), B1–C7–C8 123.36(19), N2–C7–C8 113.51(17), C7–N2–B2 123.60(18), C7–N2–C9 117.07(17), B2–N2–C9 119.31(18); C1–B1–C7–N2 7.8(4), N1–B1–C7–C8 8.9(3), B1–C7–N2–B2 61.6(3).

**Fig. 2:**
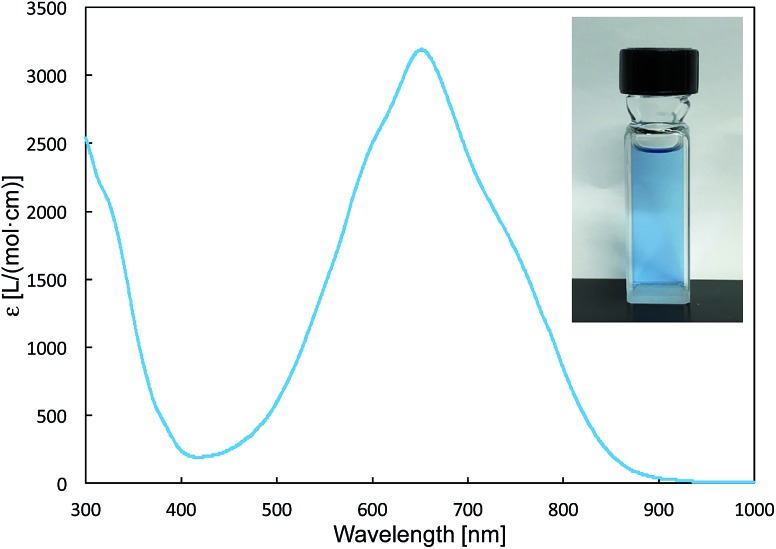
UV-vis spectrum of **4** (hexane), inset: photo of a hexane solution of **4**.

**Fig. 3:**
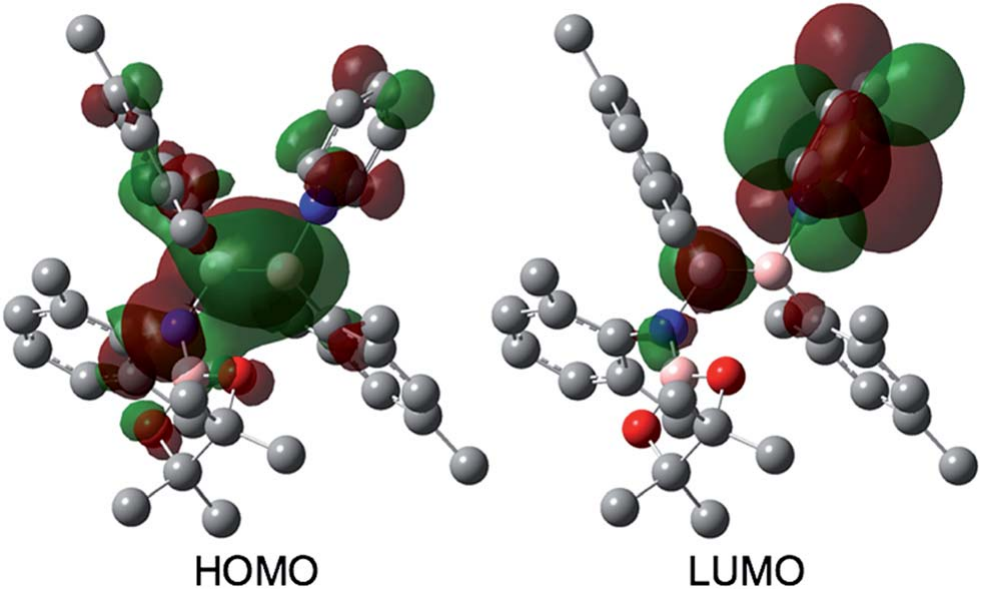
Frontier orbitals of **4** calculated at the B3LYP/6-31+G(d) level of theory.

The reaction of a toluene solution of **1** with one equivalent of Xyl-NC in the presence of pyridine (10 equiv.) afforded pyridine-coordinated boraalkene **4**. Compound **4** is probably formed *via* a cleavage of the B–B bond and a migration of the Mes group from the boron to the carbon atom (Scheme 2), as confirmed by a single-crystal X-ray diffraction analysis (Fig. 1; B1–C7: 1.441(3) Å). The length of the B–N (pyridine) bond (1.586(3) Å) is essentially identical to that of a twisted pyridine–boraalkene complex.^22^ It should be noted that **4** contains two Mes groups *trans* to each other, which is slightly different from the case of previously reported **3**, and thus indicates that the steric difference between Xyl-NC and ^*t*^Bu-NC may determine the regiochemistry of the products **3** and **4** upon coordination to the boraalkene intermediate. Interestingly, the UV-vis spectrum of a hexane solution of boraalkene **4** showed an intense blue color, with an absorption maximum at 648 nm (Fig. 2). In hexane solution, boraalkene **4** gradually decomposed at room temperature (*cf.* ESI†). This decomposition of **4** is decelerated in the presence of pyridine, indicating that the decomposition could be initiated by a dissociation of pyridine from **4**. Although all the decomposed products could not be identified, monitoring the decomposition by ^1^H NMR spectroscopy indicated that **2** was involved as a reaction intermediate (*cf.* ESI†). DFT calculations at the B3LYP/6-31+G(d) level of theory revealed that the HOMO orbital of **4** consists mainly of the B=C π bond, and that the LUMO orbital corresponds to the π*-orbital of the pyridine moiety (Fig. 3). TDDFT calculations revealed that the absorption of **4** at 648 nm corresponds to the HOMO–LUMO transition, indicative of an intramolecular charge-transfer character from the electron-rich boraalkene moiety to the electron-poor acid-coordinated pyridine moiety.

**Scheme 3:**
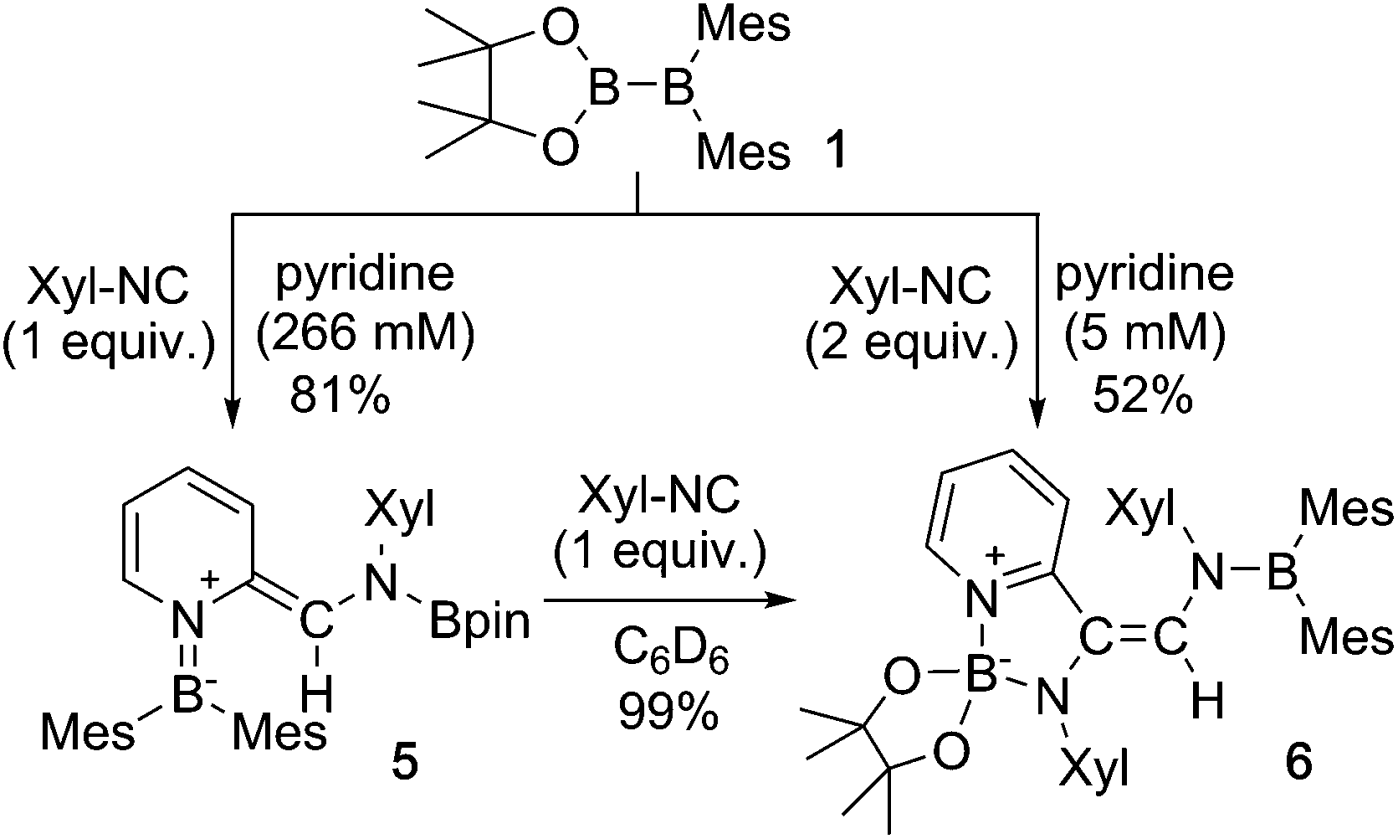
Reaction of **1** with Xyl-NC in the presence of an excess of pyridine (yields estimated by ^1^H NMR spectroscopy. The concentration of **1** or **5** is given in parentheses below pyridine).

**Fig. 4:**
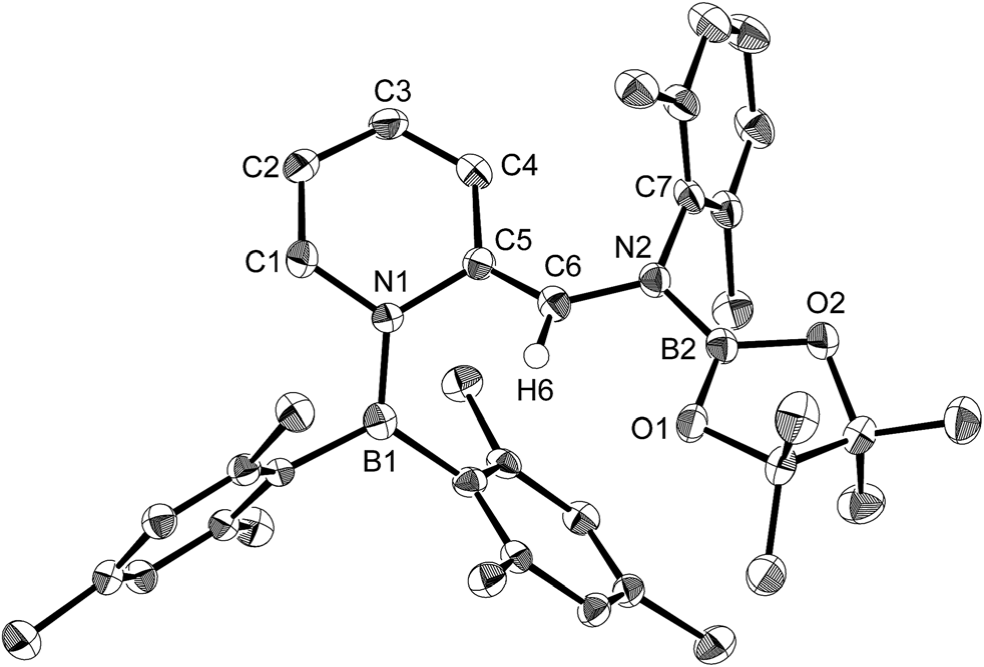
Molecular structure of **5** (thermal ellipsoids set at 50% probability; hydrogen atoms except for H6 omitted for clarity). Selected bond distances (Å), angles (°) and dihedral angles (°): B1–N1 1.428(4), N1–C1 1.425(4), C1–C2 1.334(5), C2–C3 1.449(5), C3–C4 1.344(5), C4–C5 1.467(5), N1–C5 1.457(4), C5–C6 1.354(4), C6–N2 1.412(4), N2–B2 1.425(5), B2–O1 1.376(4), B2–O2 1.386(4); B1–N1–C5 124.2(3), N1–C5–C6 114.8(3), C5–C6–N2 130.0(3), C6–N2–B2 118.6(3); B1–N1–C5–C6 –45.5(4), N1–C5–C6–N2 169.6(3), C5–C6–N2–B2 –167.2(3), C6–N2–B2–O1 –11.3(5).

**Fig. 5:**
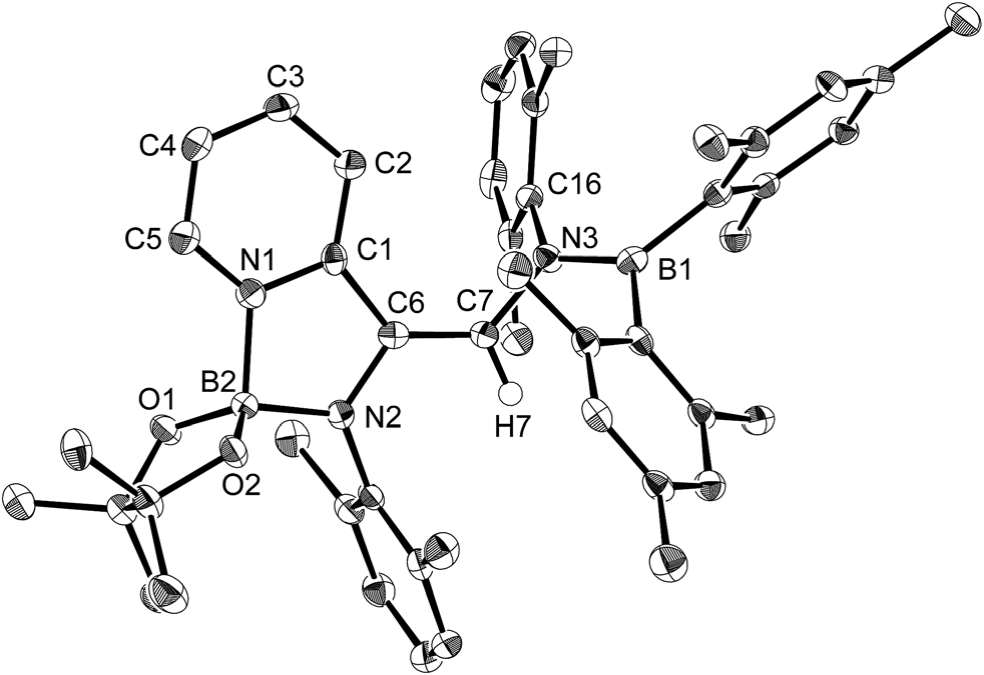
Molecular structure of **6** (thermal ellipsoids set at 50% probability; co-crystallized molecules of benzene and hydrogen atoms apart from H7 omitted for clarity). Selected bond distances (Å) and angles (°): N1–B2 1.640(4), N1–C1 1.365(3), C1–C2 1.390(4), C2–C3 1.382(4), C3–C4 1.393(4), C4–C5 1.374(4), C1–C6 1.471(4), N1–C5 1.338(3), B2–N2 1.533(4), N2–C6 1.388(3), C6–C7 1.353(4), C7–N3 1.450(3), B1–N3 1.436(3); N2–B2–N1 94.5(2), C1–N1–B2 112.4(2), C6–N2–B2 116.3(2), N1–C1–C6 109.4(2), N2–C6–C1 106.9(2).

Using the same combination of reagents, albeit in higher concentration, *i.e.*, by using pyridine as a solvent, afforded **5** (Scheme 3), which was formed *via* the cleavage of the B–B bond in **1** and the C–H bond in pyridine, in 81% NMR yield under concomitant formation of a small amount of **4** (16%). A single-crystal X-ray diffraction analysis revealed that **5** contains a dearomatized pyridine ring that exhibits a distorted quinoid structure, evident from the short B1–N1 and C5–C6 bond distances and the bond alternation in the pyridine ring (Fig. 4). Reflecting the restricted rotation of the substituents due to the distorted structure, the ^1^H and ^13^C NMR spectra of **5** exhibited several broad signals (*cf.* ESI†). Reaction of **1** with two equivalents of Xyl-NC under slightly lower concentration resulted in the formation of **6**, in which two carbon atoms of two isocyanide molecules were reductively coupled, and the resulting NCCN moiety was inserted into the B–B bond of **1** and the C–H bond of pyridine. A crystallographic analysis of **6** revealed an intramolecular coordination of the pyridine ring to the Bpin moiety, resulting in the formation of a spiroborate structure (Fig. 5). In solution, the ^11^B NMR spectrum of **6** showed three broad signals, implying an equilibrium of **1** with other isomers. This equilibrium prevented us from assigning all signals in the ^1^H NMR spectrum of **6** at room temperature. When a CD_2_Cl_2_ solution of **6** was cooled to –80 °C, some of the aromatic signals of each isomer, which can be distinguished by COSY measurements, changed their integration ratio to support the notion of such an equilibrium (*cf.* ESI†). It should be noted that **5** could be considered as an intermediate for the formation of **6**. This hypothesis was independently confirmed by addition of 1 equiv. of Xyl-NC to isolated **5**, which result in the formation of **6** in high yield.

Next, we investigated the dependency of the product ratio between **4** and **5** on the concentration of **1** and the stoichiometry of pyridine in the reaction of **1** with 1 equiv. of Xyl-NC (Table 1). When pyridine was used as the solvent, dearomatized **5** was the major product for any concentration of **1** (runs 1–4). Maintaining the concentration of **1** at 266 mM, which produced the largest amount of **5** in run 1, by keeping the volume of the total solvent (0.300 mL) constant, the amount of the added toluene was varied to change the stoichiometry of pyridine (runs 1 and 5–7). Reducing the stoichiometry of pyridine led to a higher and lower yield of **4** and **5**, respectively. These results indicate that the formation of **5**, *via* a cleavage of the C–H bond requires an excess of pyridine.

**Table 1:** Dependency of the product ratio between **4** and **5** on the concentration of **1** and the stoichiometry of pyridine in the reaction of **1** with **1** equiv. of Xyl-NC

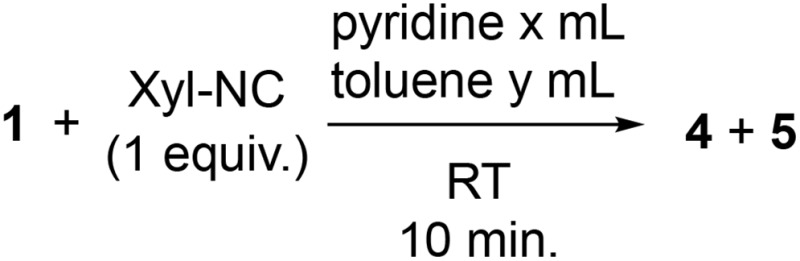						
Run	Conc.^*a*^ (mM)	*x* (mL)	*y* (mL)	Pyridine (equiv.)	Yield^*b*^ (%)	
					**4**	**5**
1	266	0.3	0	46	16	81
2	100	0.798	0	124	15	67
3	10	8	0	1240	15	79
4	5	16	0	2481	12	68
5	266	0.193	0.107	30	15	54
6	266	0.0970	0.203	15	22	54
7	266	0.0323	0.268	5	39	37

^*a*^Concentration of **1**.

^*b*^Estimated ^1^H NMR yield.

We also investigated the dependency of the product ratio among **3**, **4** and **6** on the concentration of **1** and the stoichiometry of pyridine in the reaction of **1** with 2 equiv. of Xyl-NC (Table 2). When pyridine was used as the solvent, the yield of the C–C coupled product **6** increased upon lowering the concentration of **1** (runs 1–4), which stands in contrast to the results of Table 1. Maintaining the concentration of **1** at 5 mM, which produced the highest yield of **6** (run 4), and fixing the volume of the total solvent to 16 mL, the amount of toluene added was varied to reduce the stoichiometry of pyridine (runs 5–8). Considering that reducing the stoichiometry of pyridine led to a significant increase of **3** and **4**, and that **5** is an intermediate for the formation of **6**, it seems feasible to expect that a large amount of pyridine is required for the formation of **5** and **6**. These results indicate that the complexation of **1** with pyridine prior to a reaction with Xyl-NC is the key step for the cleavage of the C–H bond of pyridine.

**Table 2:** Dependency of the product ratio among **4**, **5** and **6** on the concentration of **1** and the stoichiometry of pyridine in the reaction of **1** with **2** equiv. of Xyl-NC

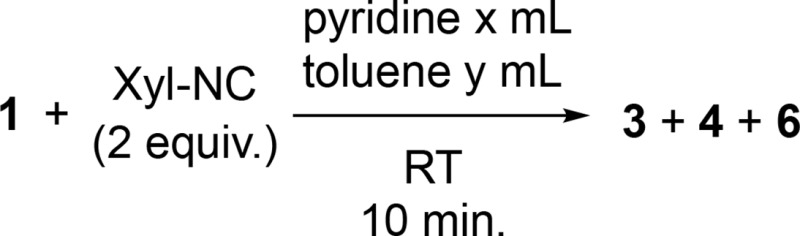							
Run	Conc.^*a*^ (mM)	*x* (mL)	*y* (mL)	Pyridine (equiv.)	Yield^*b*^ (%)		
					**3**	**4**	**6**
1	200	0.4	0	62	49	7	25
2	100	0.798	0	124	35	10	40
3	10	8	0	1240	5	9	49
4	5	16	0	2481	1	9	52
5	5	6.5	9.5	1000	6	13	50
6	5	0.645	15.4	100	20	40	18
7	5	0.0645	15.9	10	22	32	2
8	5	0.0323	16	5	28	46	1

^*a*^Concentration of **1**.

^*b*^Estimated ^1^H NMR yield.

**Scheme 4:**
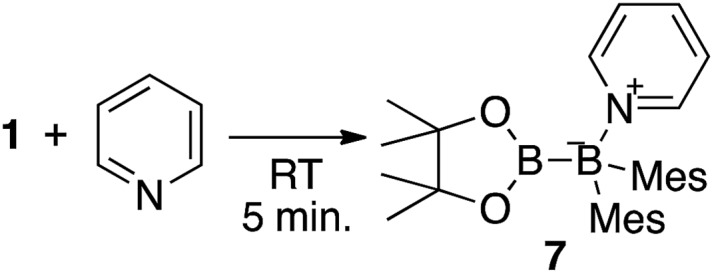
Reaction of **1** with pyridine.

**Fig. 6:**
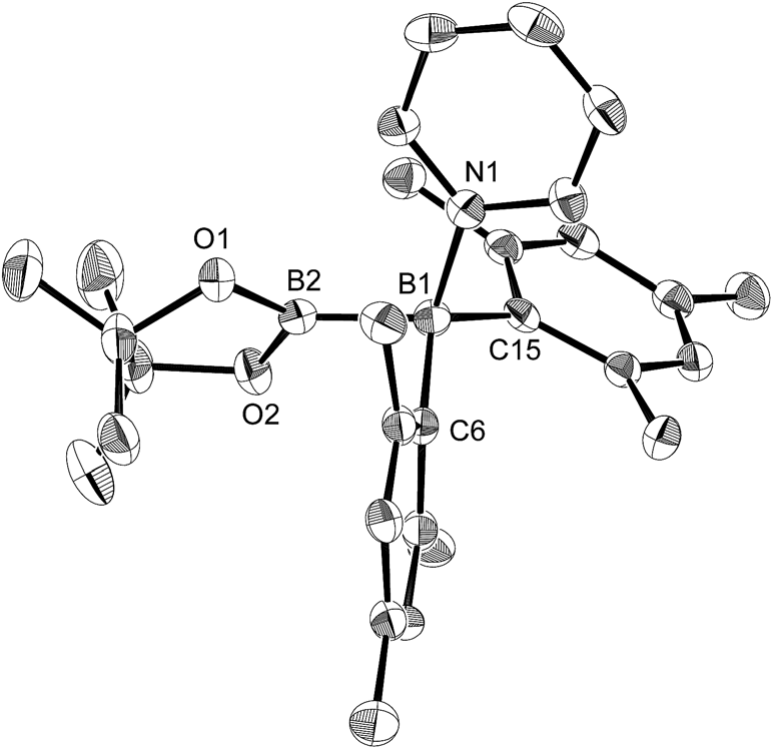
Molecular structure of **7** (thermal ellipsoids set at 50% probability; hydrogen atoms omitted for clarity). Selected bond distances (Å) and angles (°): B1–N1 1.653(3), B1–B2 1.717(3), B1–C6 1.665(3), B1–C15 1.644(3), B2–O1 1.396(3), B2–O2 1.385(3); B2–B1–N1 111.34(16), B2–B1–C6 94.31(16), B2–B1–C15 119.02(17), C6–B1–C15 119.62(17), C6–B1–N1 112.34(16), C15–B1–N1 100.77(15).

In order to determine the mechanism for the formation of **5** and **6**, a potential intermediate was synthesized and isolated from the reaction of **1** with pyridine (Scheme 4). Simple dissolution of **1** in pyridine followed by recrystallization from hexane afforded pyridine-coordinated sp^2^–sp^3^ species **7**. A C_6_D_6_ solution of **7** exhibited two broad signals in the ^11^B NMR spectrum at *δ*_B_ = 37 and 26 ppm. The former signal was assigned to an sp^3^-hybridized BMes_2_ moiety, even though it is high-field shifted in comparison with the BMes_2_ moiety of **1** (*δ*_B_ = 89 ppm).^18^ The presence of this signal indicates that the coordination of pyridine is retained in C_6_D_6_. The sp^2^–sp^3^ structure of **7** was unambiguously determined by single-crystal X-ray diffraction analysis (Fig. 6). Despite the coordination of pyridine, the B–B bond (1.717(3) Å) of **7** was identical to that of **1** (1.722(4) Å).^23^,^24^ The hybridization of the B1 atom is slightly distorted (B2–B1–C6 = 94.31(16)°) from ideal sp^3^ hybridization as observed in similar sp^2^–sp^3^ diborane(4) derivatives that contain a BMes_2_ moiety,^25^ which is probably due to the steric demand of the Mes groups.

**Scheme 5:**
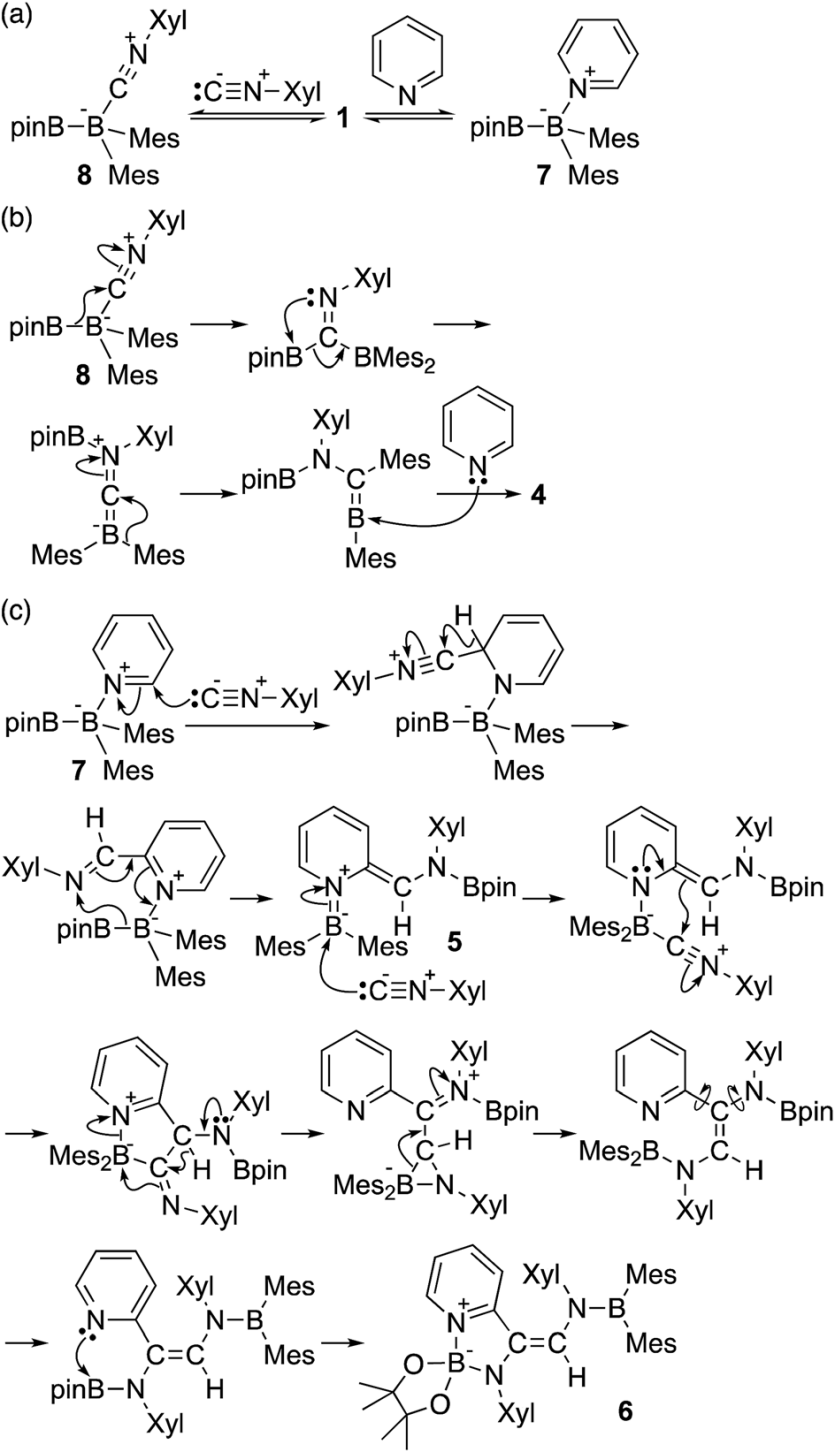
Plausible reaction mechanism for the formation of **4–7**.

Based on the product ratio and the formation of potential intermediate **7**, we tentatively propose a mechanism for the formation of **4–7** (Scheme 5). In reaction mixtures of **1**, Xyl-NC and pyridine, **1** should be coordinated by Xyl-NC and pyridine to form sp^2^–sp^3^ adducts **8** and **7** (Scheme 5a). According to our previous report on the reaction of **1** with ^*t*^Bu-NC, a boraalkene intermediate could be generated by three consecutive rearrangements, involving a pinB migration to the carbon atom of the isocyanide, a pinB capturing by the nitrogen atom, and an electrophilic migration of the Mes group toward the (push–pull-stabilized) carbenic carbon (Scheme 5b). Coordination of pyridine to the boraalkene intermediate furnished **4**. This mechanism is consistent with the fact of that decreasing the amount of pyridine led to a higher yield of **4** (Tables 1 and 2). Pyridine-coordinated intermediate **7** was initially attacked by the isocyanide at the 2-position of pyridine leading to a dearomatization of the pyridine ring (Scheme 5c).^26^ A subsequent 1,2-hydride migration would lead to the formation of an imidoylpyridine-coordinated sp^2^–sp^3^ adduct, and an ensuing nucleophilic migration of the Bpin group to the nitrogen atom would directly afford dearomatized quinoid **5**. Further coordination of a second molecule of isocyanide to **5** would induce the formation of a five-membered ring. The subsequent nitrogen-induced 1,2-hydride migration could cleave the N–B coordination bond and generate the three-membered cyclic intermediate. Breaking the azaaboracyclopropane ring should then furnish the diaminopyridylalkene intermediate. The subsequent formation of an intramolecular N–B coordination would finally generate **6**.

**Scheme 6:**
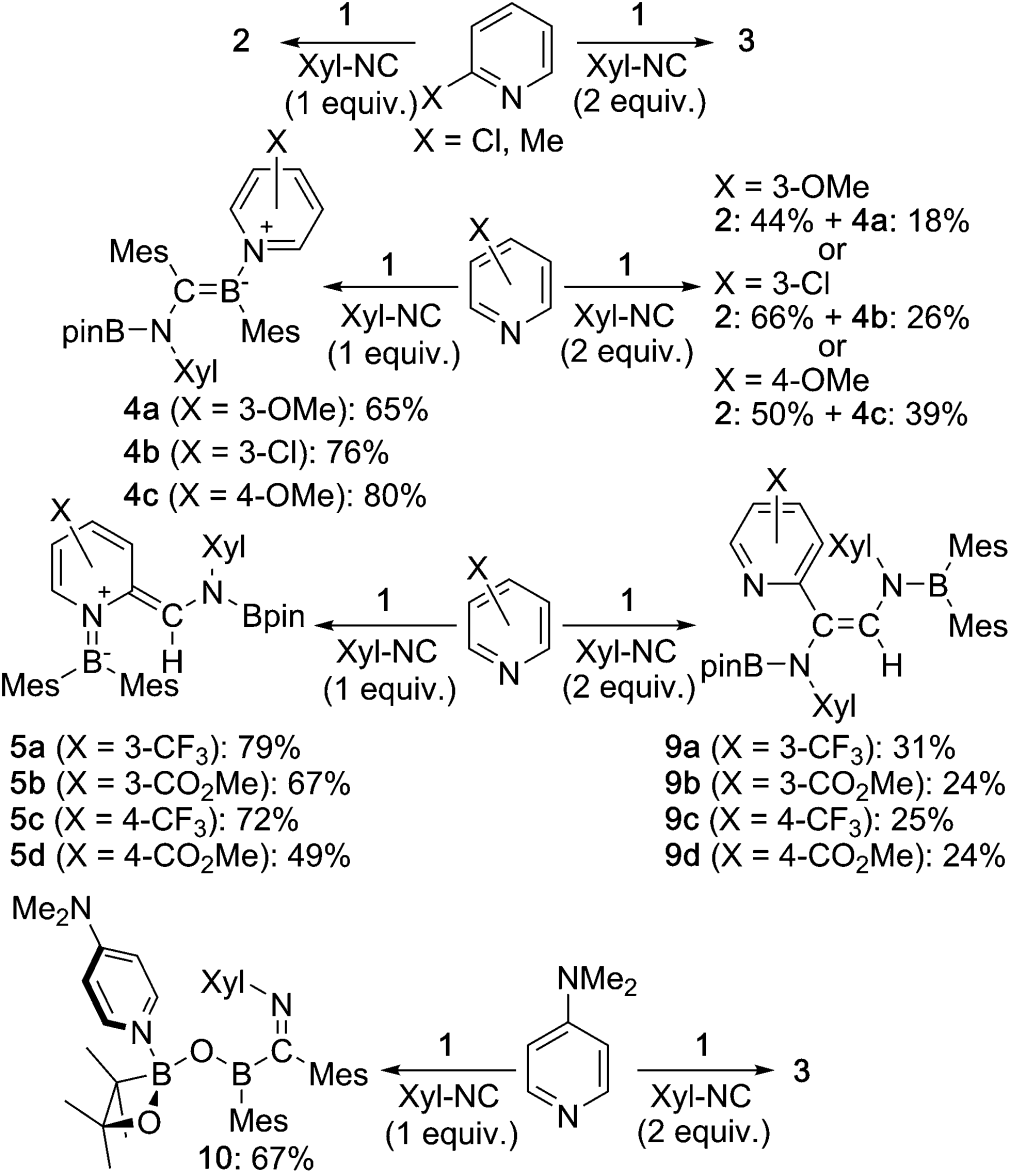
Reaction of **1** with substituted pyridine derivatives and Xyl-NC (yields estimated by ^1^H NMR spectroscopy).

**Fig. 7:**
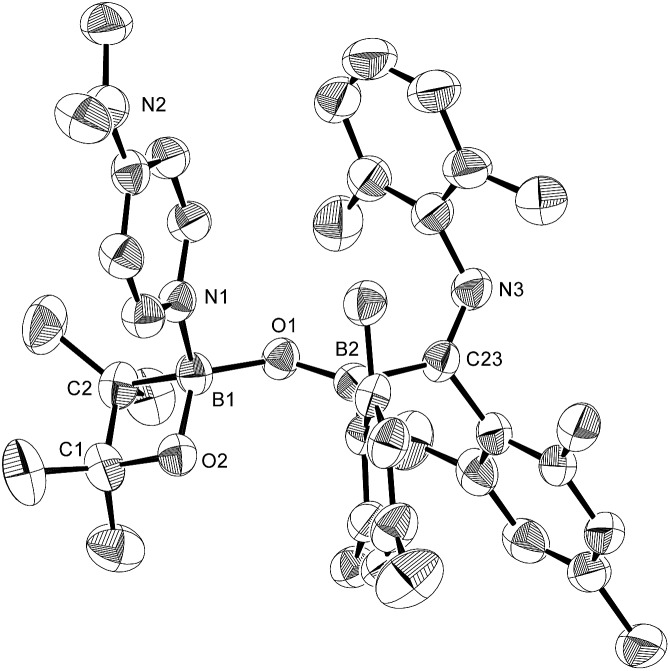
Molecular structure of **10** (thermal ellipsoids set at 50% probability; co-crystallized molecules of toluene and hydrogen atoms omitted for clarity). Selected bond distances (Å), angles (°) and dihedral angles (°): B1–O1 1.470(4), B1–O2 1.465(4), B1–C2 1.628(5), B1–N1 1.612(4), O1–B2 1.330(4), B2–C23 1.641(5), C23–N3 1.295(4); O2–B1–O1 117.0(3), O2–B1–N1 110.6(2), O1–B1–N1 105.2(2), O2–B1–C2 91.6(2), O1–B1–C2 118.4(3), N1–B1–C2 113.9(3), B1–O2–C1 91.2(2), B1–O1–B2 128.6(3), O1–B2–C23 118.1(3), B2–C23–N3 127.9(3); B1–O1–B2–C23 176.5(3), O1–B2–C23–N3 63.4(4).

Then, we examined the substrate scope of the present reaction with respect to pyridine derivatives (Scheme 6). In the case of *ortho*-substituted pyridines, such as 2-methylpyridine and 2-chloropyridine, only **2** and **3** were obtained without incorporation of pyridine-derivatives. The steric hindrance of the *ortho*-substituent should thus inhibit the coordination of pyridine and the subsequent reactions outlined in the proposed mechanism (Scheme 5c). In the case of pyridine derivatives that contain electron-donating or weakly electron-withdrawing groups,^27^ such as 3-MeO, 3-Cl and 4-MeO, pyridine-coordinated boraalkene derivatives **4a**, **4b** and **4c** were obtained, respectively. Similar to the case of **4**, **4a–c** gradually decomposed in solution at room temperature, which hampered a characterization by ^13^C NMR spectroscopy. We were however able to confirm the formation of sp^2^–sp^3^ diborane intermediates **7a–c**, which are similar to **7**, through coordination of pyridine derivatives to **1** as evident from ^11^B NMR spectroscopy (*cf.* ESI†). Therefore, we conclude that the nucleophilic attack of the Xyl-NC on the pyridine derivatives should be suppressed (Scheme 5c). Upon increasing the amount of Xyl-NC to two equivalents, mixtures of **2** and **4a–c** were obtained. This result is consistent with Scheme 5a, where the equilibrium is shifted to the left upon increasing the amount of Xyl-NC in order to prevent the formation of pyridine-incorporated products **4a–c**. When using pyridines that contain strongly electron-withdrawing groups, such as 3-CF_3_, 3-MeOCO, 4-CF_3_ or 4-MeOCO, *ortho*-functionalized pyridine derivatives **5a–d** and **9a–d** were obtained, depending on the stoichiometry of Xyl-NC. These compounds are similar to **5** and **6** in the reaction of non-substituted pyridine, although **9a–d** do not exhibit intramolecular N–B coordination in the crystal structure (*cf.* ESI†), which is probably due to the presence of the electron-withdrawing group on the pyridine. Reaction of **1** with one equivalent of Xyl-NC and 4-(*N*,*N*-dimethylamino)pyridine (DMAP) afforded oxaboretane **10** (Fig. 7), which can be considered as a ring-opened derivative of **2** upon coordination of DMAP to the boron atom in the four-membered ring. In fact, a reaction of isolated **2** and DMAP smoothly furnished **10**. The reaction of **1** with two equivalents of Xyl-NC and DMAP simply afforded **3**, whereby DMAP was not incorporated in the product. Thus, the balance of the coordinating ability of the pyridine derivative and the stoichiometry of Xyl-NC affects the structure of the products.

**Scheme 7:**
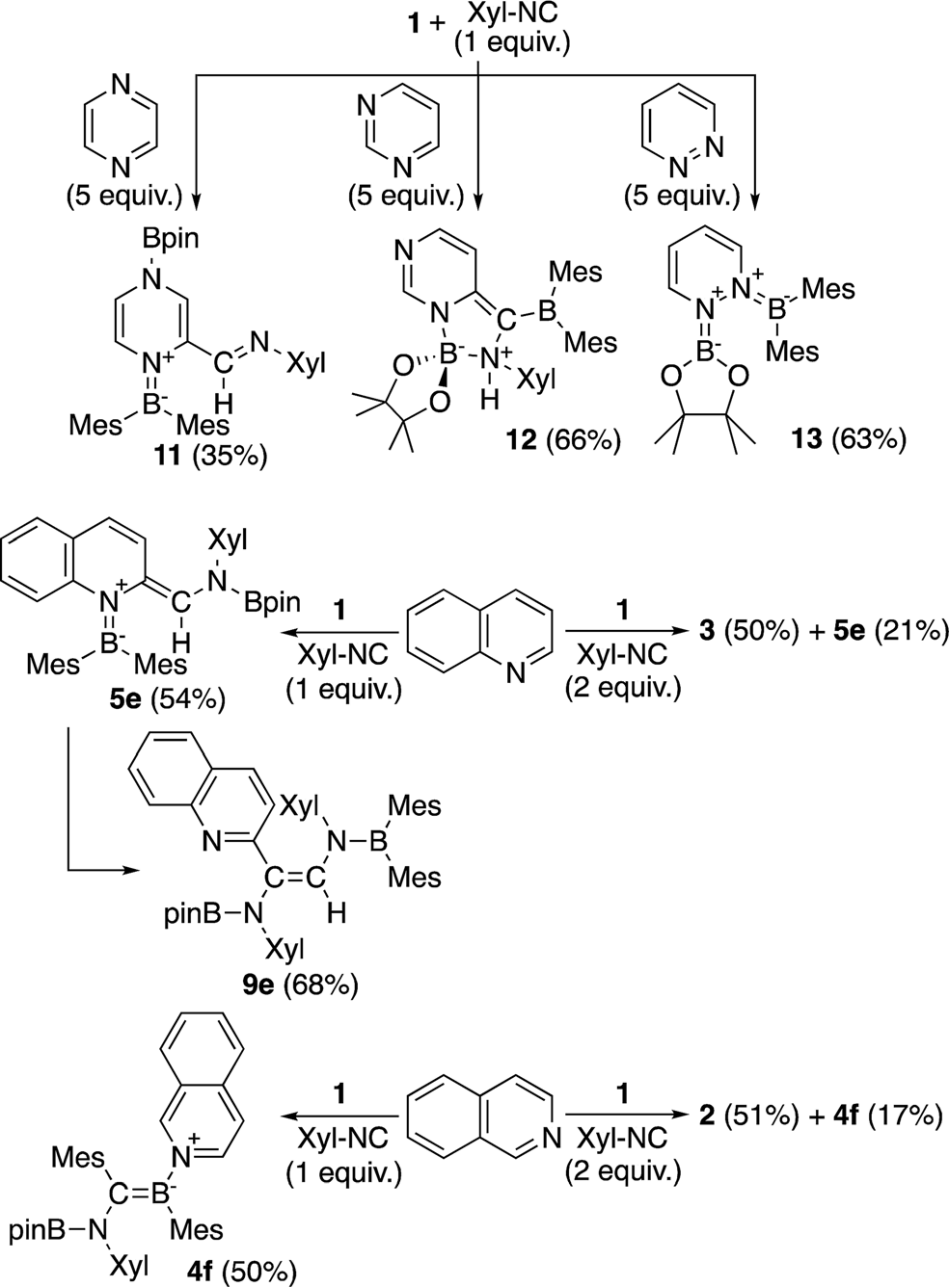
Reaction of **1** with Xyl-NC in the presence of N-heterocycles other than pyridines (yields estimated by ^1^H NMR spectroscopy).

**Fig. 8:**
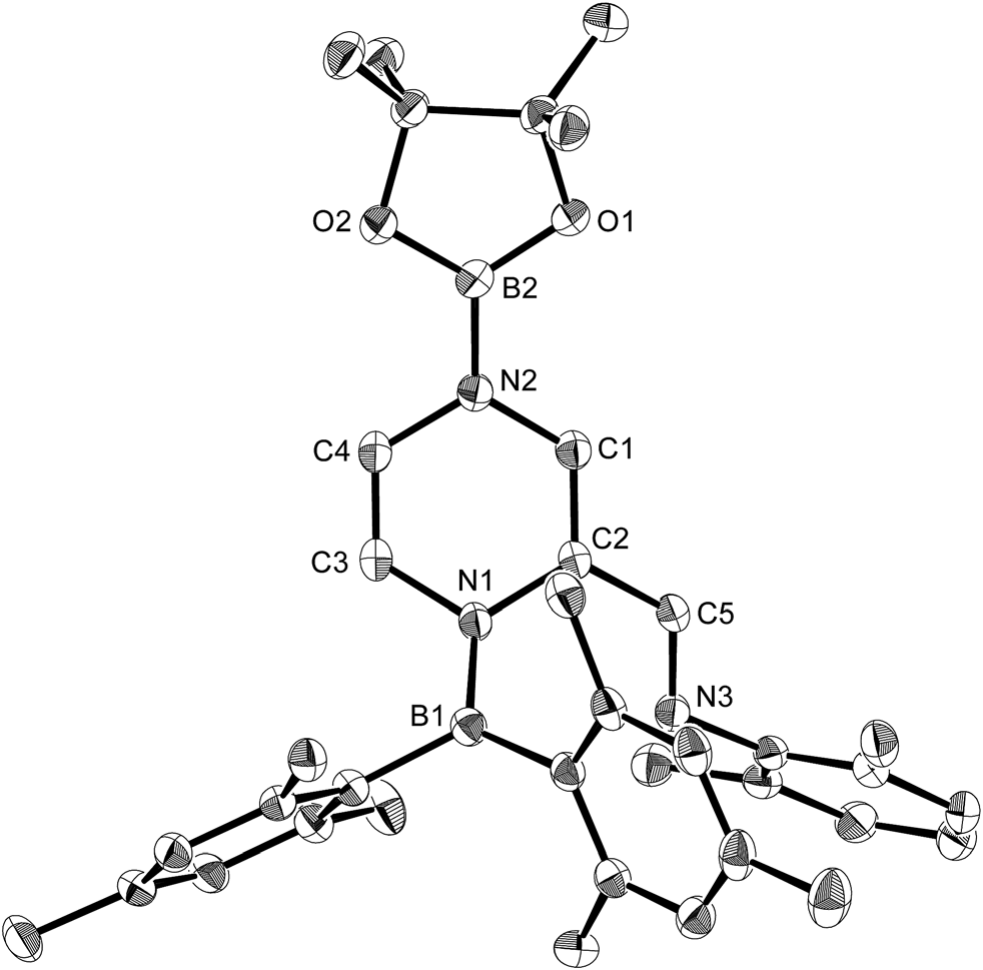
Molecular structure of **11** (thermal ellipsoids set at 50% probability; hydrogen atoms omitted for clarity). Selected bond distances (Å), angles (°) and dihedral angles (°): N1–B1 1.421(2), N1–C2 1.435(2), N1–C3 1.431(2), C1–C2 1.346(2), N2–C1 1.395(2), N2–C4 1.421(2), C3–C4 1.321(3), C2–C5 1.459(2), C5–N3 1.278(2), O1–B2 1.370(2), O2–B2 1.373(2); B1–N1–C2 127.08(14), B1–N1–C3 120.90(14), N1–C2 C5 120.52(15), C2–C5–N3 122.87(15); B1–N1–C2–C5 43.8(2), N3–C5–C2–N1 6.6(3), C3–N1–C2–C1 26.6(2).

**Fig. 9:**
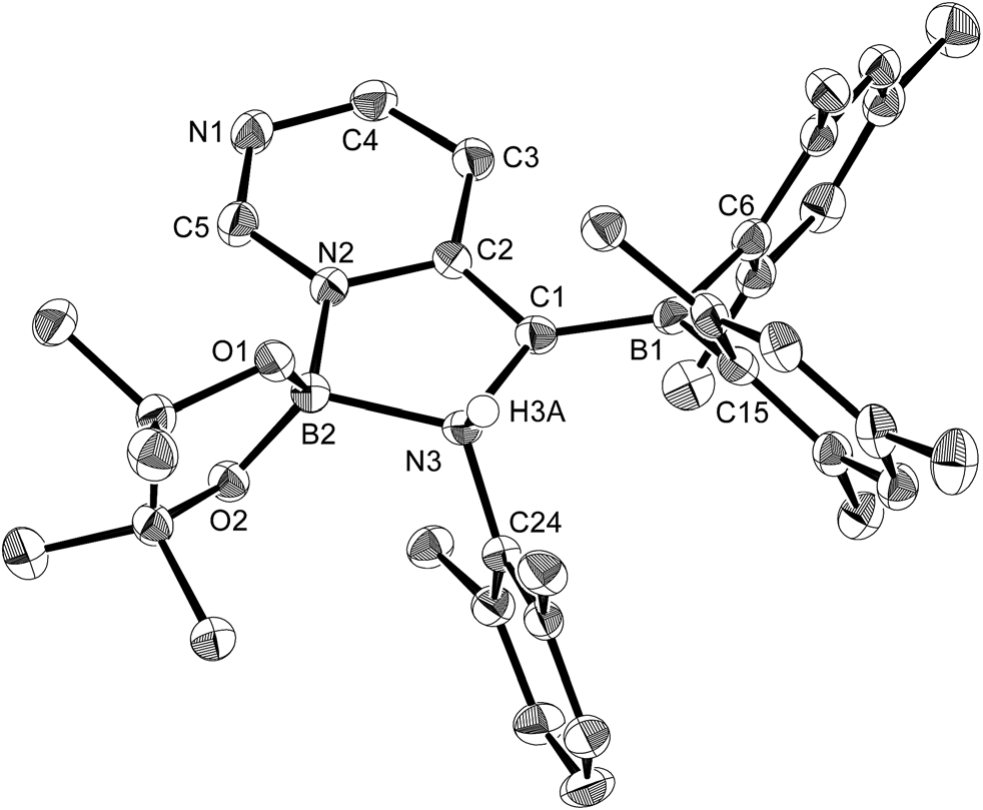
Molecular structure of **12** (thermal ellipsoids set at 50% probability; hydrogen atoms except for H3A omitted for clarity). Selected bond distances (Å), angles (°) and dihedral angles (°): N2–B2 1.570(3), N3–B2 1.691(3), N2–C5 1.352(3), N1–C5 1.304(3), N1–C4 1.381(3), C4–C3 1.360(3), C3–C2 1.426(3), N2–C2 1.385(3), C1–C2 1.391(3), C1–B1 1.509(3), N3–C1 1.510(3), O1–B2 1.432(3), O2–B2 1.410(3); C5–N2–C2 120.4(2), C5–N2–B2 125.57(19), C2–N2–B2 113.99(18), N1–C5–N2 126.2(2), C3–C4–N1 124.5(2), C4–C3–C2 118.6(2), C2–C1–B1 128.1(2), C2–C1–N3 107.25(18), B1–C1–N3 123.21(19), O2–B2–O1 109.03(19), N2–B2–N3 95.50(16); C24–N3–C1–B1 –62.2(3), C24–N3–B2–O1 112.7(2), C24–N3–B2–N2 –127.28(18).

**Fig. 10:**
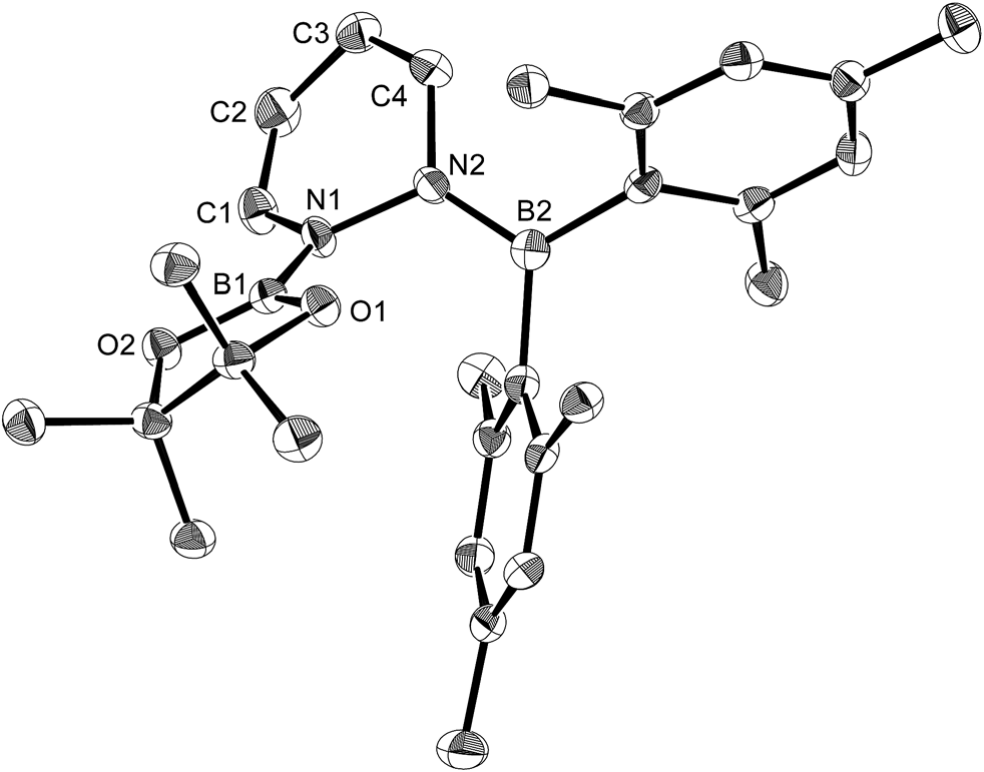
Molecular structure of **13** (thermal ellipsoids set at 50% probability, hydrogen atoms omitted for clarity). Selected bond distances (Å), angles (°) and dihedral angles (°): N1–B1 1.418(3), C1–N1 1.407(3), C1–C2 1.328(3), C3–C2 1.453(3), C4–C3 1.334(3), N2–C4 1.418(3), N2–B2 1.410(3), O1–B1 1.370(3), O2–B1 1.369(3); C1–N1–B1 123.90(18), C1–N1–N2 115.70(17), B1–N1–N2 119.74(17), C2–C1–N1 119.9(2), C1–C2–C3 118.1(2), C4–C3–C2 119.7(2), C3–C4–N2 119.8(2), C4–N2–N1 112.21(16), B2–N2–C4 126.36(19), B2–N2–N1 121.17(18); B2–N2–N1–B1 58.4(3), B2–N2–N1–C1 –130.6(2), C4–N2–N1–C1 43.9(2).

The present reaction was also expanded to include N-heterocycles other than pyridines, which afforded a variety of products (Scheme 7) that were all characterized by single-crystal X-ray diffraction analysis (*cf.* ESI†). The reaction of **1** with pyrazine and Xyl-NC furnished diboryl pyrazine **11**, which contains a 1,4-dihydropyrazine core and an imine functionality that is derived from Xyl-NC (Fig. 8). The formation of **11** may be explained by a prior formation of a quinoid intermediate similar to **5a–d** and a subsequent intramolecular 1,5-migration or intermolecular transfer of a Bpin group to the nitrogen atom (*cf.* ESI†). In the case of pyrimidine, spirocyclic borate **12** with a dearomatized pyrimidine ring was obtained (Fig. 9). Although the mechanism for the formation of **12** is not clear yet, we would like to postulate a mechanism based on those for similar quinoid intermediates (*cf.* ESI†). The reaction of **1** with pyridazine in the presence of Xyl-NC afforded *ortho*-quinodimethane **13** (Fig. 10), which contains two borylated nitrogen atoms. The formation of **13** could be explained by a direct addition of **1** to the N=N double bond of pyridazine. Quinoline could also be used in the same reaction to give *ortho*-functionalized derivatives **5e** and **9e**, depending on the stoichiometry of Xyl-NC, similarly to the case of the formation of **5a–e** and **9a–e** (Scheme 5). On the contrary, isoquinoline also reacted with **1** and Xyl-NC, but C–H functionalized quinoline derivatives were not obtained.

**Scheme 8:**
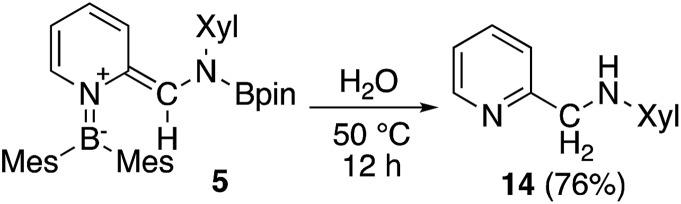
Hydrolysis of **5**.

The *ortho*-functionalized pyridine derivative **5** was then further converted (Scheme 8). Heating **5** with H_2_O in THF to 50 °C for 12 h afforded the aminomethylated pyridine **14**^28^ in 76% yield. This two-step procedure to obtain **14** from non-substituted pyridine surpasses previously reported synthetic protocols which includes at least five steps.^28^,^29^ Therefore, the present method can be considered as an effective route for the functionalization of pyridine. However, limitations exist, especially for examples where the isocyanide substrate is not readily accessible. Although some metal-catalyzed *ortho*-functionalizations of pyridine with aldimine,^30^ carbon monoxide,3*c* and isocyanide,^31^ have been reported, this method is the only example for a selective formation of methylene-substituted pyridines. Also, the hydrolysis can be considered as a hydrogenative quenching of **5** under concomitant deoxygenation of water.

## Conclusions

In conclusion, we have discovered a reaction of the previously reported diborane(4) pinB-BMes_2_ with Xyl-NC and pyridine that affords pyridine-coordinated boraalkenes, which show an intense color caused by an intramolecular charge-transfer interaction. In the presence of an excess of pyridine, the *ortho* C–H bond of pyridine was selectively functionalized to form a quinoid compound or an isocyanide-coupled product. Based on the concentration effect, reaction stoichiometry, and previously reported DFT calculations,^18^,^20^ a reaction mechanism was proposed that involves numerous rearrangement reactions. Substituted pyridines and other N-heterocycles can also be used in the present method to afford the corresponding functionalized derivatives. A subsequent hydrolysis of one of the resulting products furnished an aminomethylated pyridine derivative, which requires several steps when using a conventional synthetic procedure.

## Conflicts of interest

There are no conflicts to declare.

## Supplementary Material

Supplementary information

Supplementary information

Crystal structure data
