# Supplementary material for: Reactivity of highly Lewis acidic diborane(4) towards pyridine and isocyanide: formation of boraalkene–pyridine complex and ortho-functionalized pyridine derivatives
Source: Chem Sci. 2017 Dec 11;9(5):1301–10. doi: 10.1039/c7sc04759b (PMC5887101; doi:10.1039/c7sc04759b)
Supplement: Supplementary file 1 [file SC-009-C7SC04759B-s001.pdf]

Electronic Supplementary Material (ESI) for Chemical Science.
This journal is © The Royal Society of Chemistry 2017

Supporting Information for

Reactivity of Highly Lewis Acidic Diborane(4) towards Pyridine and Isocyanide: Formation of Boraalkene-Pyridine Complex and *ortho*-Functionalized Pyridine Derivatives

Yuhei Katsuma,[1] Hiroki Asakawa,[1] Makoto Yamashita[2]*

[1] Department of Applied Chemistry, Faculty of Science and Engineering, Chuo University, 1-13-27, Kasuga, Bunkyo-ku, 112-8551, Tokyo, Japan.

[2] Department of Molecular and Macromolecular Chemistry, Graduate School of Engineering, Nagoya University, Furo-cho, Chikusa-ku, Nagoya, 464-8603 Aichi (Japan)

E-mail: makoto@oec.chembio.nagoya-u.ac.jp

General

All manipulations involving the air- and moisture-sensitive compounds were carried out in glovebox (KIYON, Korea and ALS Technology, Japan) under argon atmosphere. Benzene-d_6 was dried over Na/Ph_2C=O and was distilled under reduced pressure. Pentane (Super Dehydrated, Kanto Chemical. Co., Inc.) was directly used as received. NMR spectra were recorded at 22 °C on 500 or 400 MHz spectrometers unless otherwise noted. Chemical shifts are reported in ppm relative to the residual protiated solvent for ^{1}H, deuterated solvent for ^{13}C, and external $BF_3 \cdot OEt_2$ for ^{11}B nuclei. Data are presented in the following space: chemical shift, multiplicity (s = singlet, d = doublet, t = triplet, sept = septet, m = multiplet, br = broad, brs = broad singlet), coupling constant in hertz (Hz), and signal area integration in natural numbers. The unsymmetrical diborane(4) **1** was synthesized according to the literature.[1] Elemental analyses were performed at the A Rabbit Science Co., Ltd. or Instrumental Analysis Room, School of Engineering, Nagoya University. Melting points (m.p.) were determined with a MPA100 OptiMelt (Tokyo Instruments, Inc.) and were uncorrected. UV/vis spectrum was recorded on UV-3600 (Shimadzu) spectrometer with a 1 cm quartz cell. X-ray crystallographic analysis was performed on VariMax/Saturn CCD diffractometer. Mass spectra were measured on a JEOL JMS-700 mass spectrometer. Melting points (m.p.) were determined with a MPA100 OptiMelt (Tokyo Instruments, Inc.) and were uncorrected.

Synthesis of 4

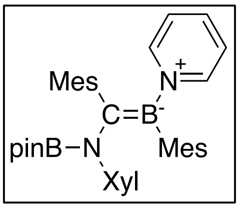

In a glovebox, a toluene solution (30 mL) of Xyl-NC (174 mg, 1.33 mmol) was added to a toluene solution (235 mL) of **1** (500 mg, 1.33 mmol) and pyridine (1.08 mL, 13.3 mmol) in a 250 mL Schlenk flask at room temperature. During the reaction mixture was stirred at room temperature for 5 min, the color of the resulting solution turned to be blue. Volatiles were removed from the reaction mixture under reduced pressure. The residue was precipitated from toluene/hexane to afford blue solids of **4** (67.2 mg, 0.115 mmol, 9%). Single crystals suitable for X-ray diffraction analysis were obtained by recrystallization from hexane (–35 °C). ^{1}H NMR (400 MHz, C_6D_6) δ

1.09 (s, 12H, CH_3 of pin), 2.19 (s, 3H, *p*-CH_3 of Mes), 2.25 (s, 6H, *o*-CH_3 of Ar), 2.31 (s, 3H, *p*-CH_3 of Mes), 2.51 (s, 6H, CH_3), 2.83 (s, 6H, CH_3), 5.87 (t, J = 7 Hz, 2H, 3,5-CH of pyridine), 6.13 (t, J = 7 Hz, 1H, 3-CH of pyridine), 6.77 (s, 2H, CH of Mes), 6.91 (dd, J = 7, 2 Hz, 1H, 4-CH of Xyl), 6.95-7.05 (m, 3H), 8.04 (dd, J = 7, 1 Hz, 1H, 2-CH of pyridine); ^{11}B NMR (160.5 MHz, C_6D_6) δ 29 (s), 24 (s); Decomposition of **4** in solution at room temperature (as described in the main text) prevented us to perform complete characterization with ^{13}C NMR spectrum; mp 87.0-89.8 °C (decomp.); Anal. Calcd for $C_{38}H_{48}B_2N_2O_2$: C, 77.83; H, 8.25; N, 4.78; Found: C, 77.95; H, 7.98; N, 4.51.

Estimation of NMR yield for the formation of 4

In a glovebox, a toluene solution (7.9 mL) of Xyl-NC (10.5 mg, 79.8 μmol) was added to a toluene solution (7.9 mL) of **1** (30.0 mg, 79.8 μmol) and pyridine (64.6 μL, 798 μmol) in a 30 mL vial at room temperature. After stirring the reaction mixture for 10 min at room temperature, volatiles were removed from the reaction mixture under reduced pressure. A benzene-*d*$_6$ solution (600 μL) of 1,3,5-trimethoxybenzene (13.7 mg, 81.5 μmol) was added to the residue and the resulting mixture was stirred for 5 min at room temperature. An aliquot (600 μL) of the resulting solution was pipetted to a screw-capped NMR tube. After bringing the NMR tube out from the glovebox, ^{1}H NMR spectrum was recorded to estimate the NMR yield of **4** (51%).

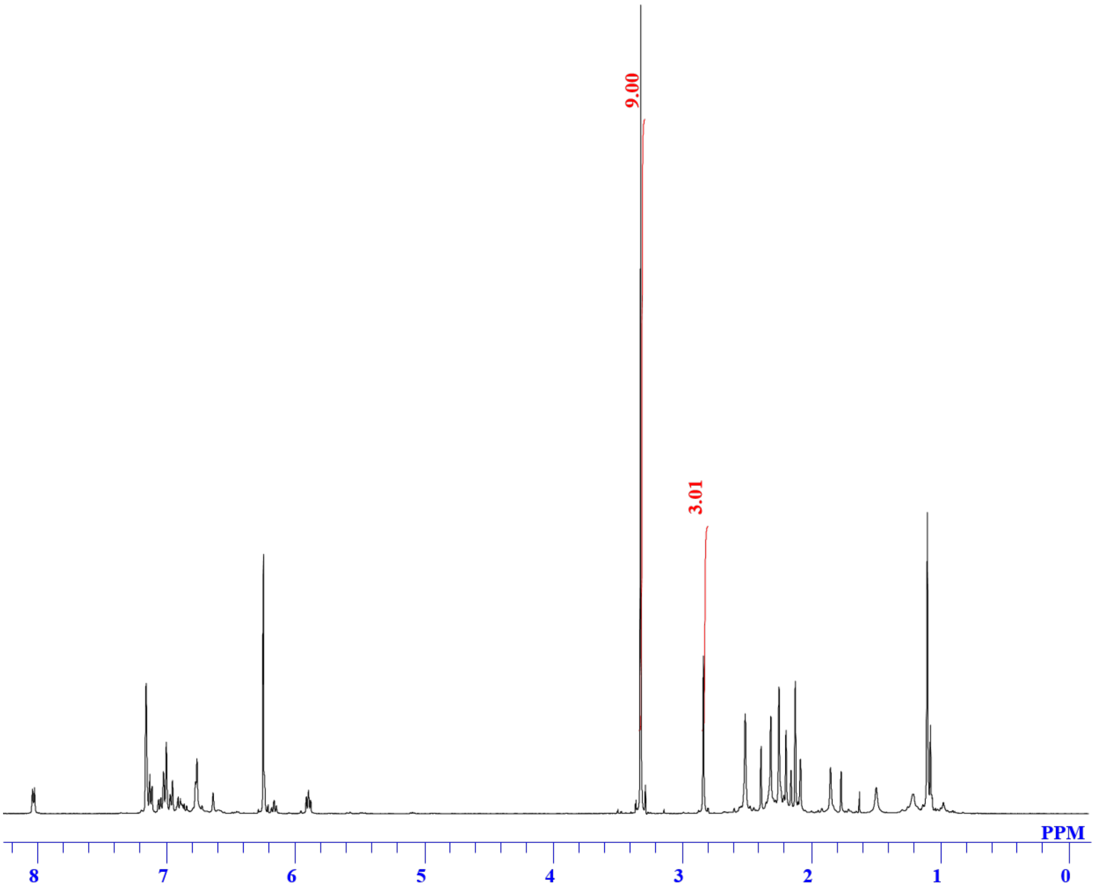

Figure S1. The ^{1}H NMR spectrum of the crude product for the synthesis of **4**

Monitoring the stability of 4 in solution by UV-vis spectroscopy

Solids of **4** (11.7 mg, 20.0 μmol) was dissolved in hexane (10.0 mL). An aliquot (1.00 mL) of the resulting solution was diluted to 10 mL with hexane in a volumetric flask two times to prepare a 100 μM solution. The

solution was pipetted into a 1 cm quarts cell. For the experiment with pyridine, a 0.300 M hexane solution (10 µL) of pyridine added to 100 µM solution (3 mL) of **4** to prepare a mixture of 4 with 10 eq. pyridine. The decay of the absorption at 648 nm was monitored with UV-vis spectroscopy.

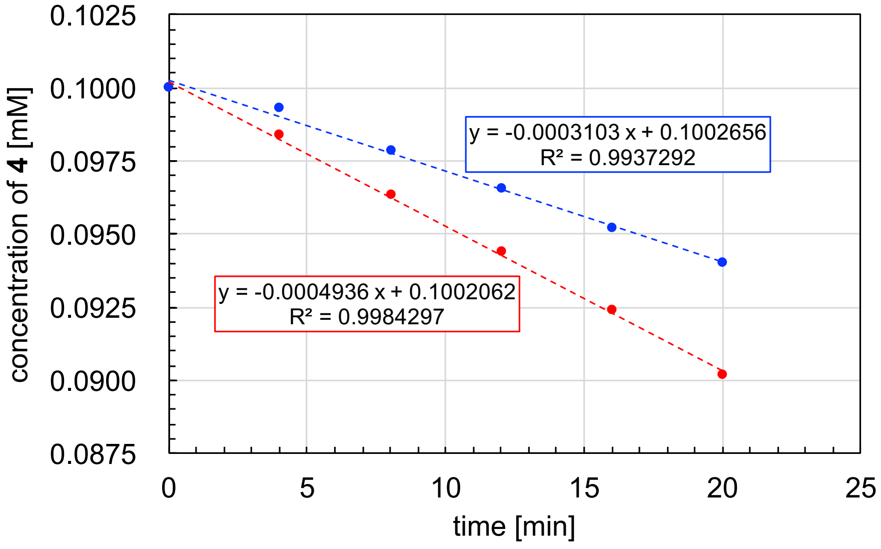

Figure S2. Time course for the decomposition of **4** by using UV-vis spectrum of **4** in hexane (monitored with the absorption maximum at 648 nm); red: no additive, blue: with 10 equiv. of pyridine

Monitoring the stability of 4 in solution by ^{1}H NMR spectroscopy

In a glove box, a freshly prepared benzene-d_6 solution (600 µL) of **4** (5.0 mg, 8.5 µmol) was pipetted to a screw-capped NMR tube. After bringing the NMR tube out from the glovebox, ^{1}H NMR spectra were recorded at 0, 13, and 24 h. In both aliphatic and aromatic region, decomposition of **4** and formation of **2** as an intermediate were detected.

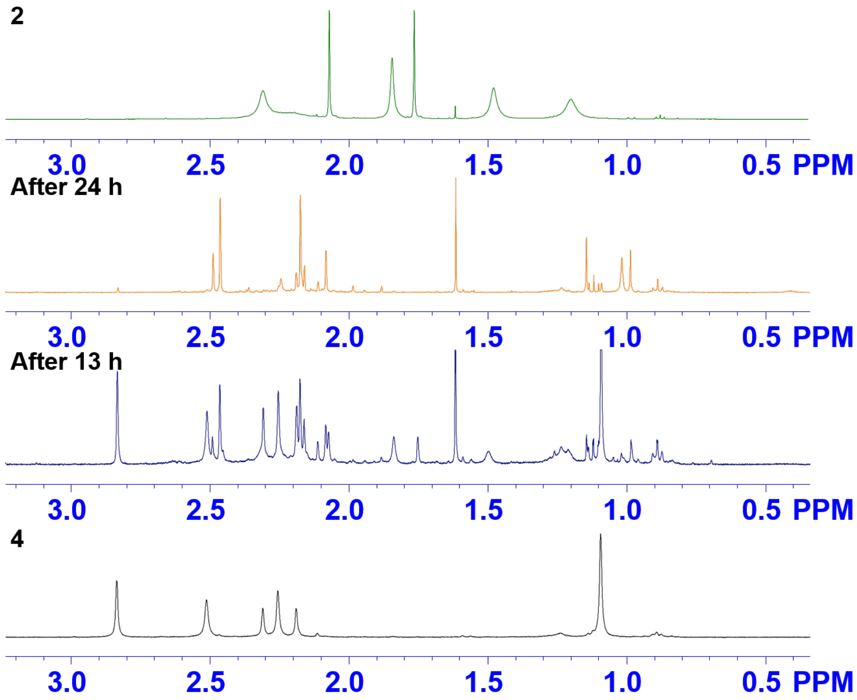

Figure S3. Time course for the decomposition of **4** by using ^{1}H NMR spectra of **4** in C_6D_6 (aliphatic region)

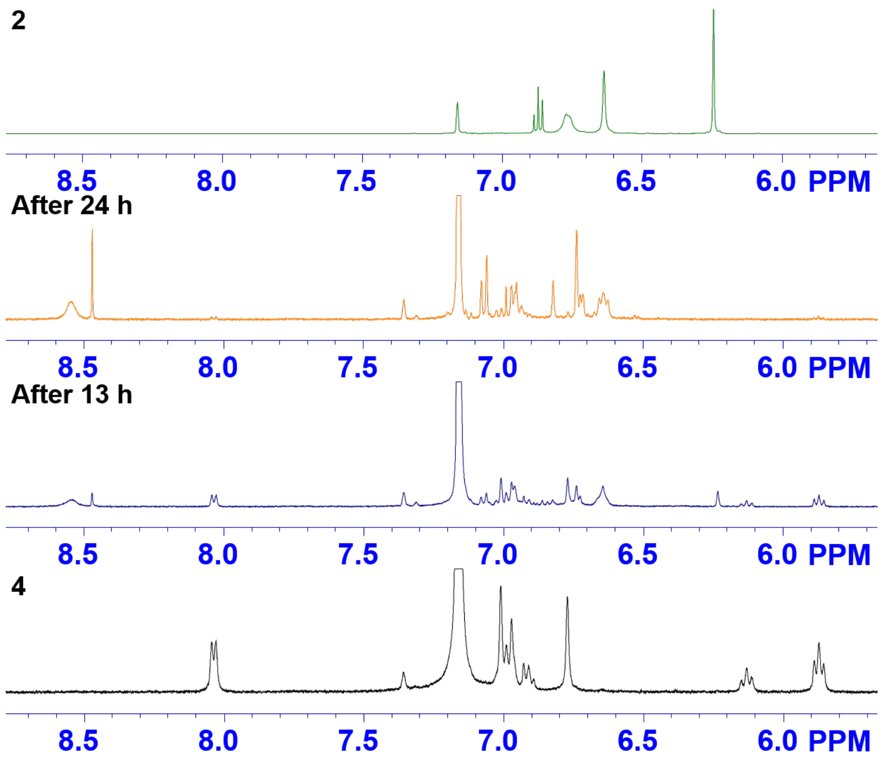

Figure S4. Time course for the decomposition of **4** by using ^{1}H NMR spectra of **4** in C_6D_6 (aromatic region)

Synthesis of 5

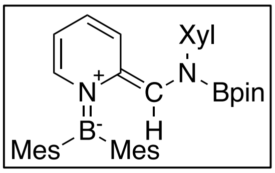

In a glovebox, a pyridine solution (15 mL) of Xyl-NC (525 mg, 4.00 mmol) was added to a pyridine solution (15 mL) of **1** (1500 mg, 3.99 mmol) in a 100 mL flask at room temperature. During the reaction mixture was stirred at room temperature for 5 min, the color of the resulting solution turned to be green. Volatiles were removed from the reaction mixture under reduced pressure. The residue was recrystallized from hexane (–35 °C) to afford yellow crystals of **5** (823 mg, 1.40 mmol, 35%). ^{1}H NMR (500 MHz, C_6D_6) δ 0.96 (br s, 12H, CH_3 of pin), 2.08 (br s, 6H, *o*-CH_3 of Mes or Xyl), 2.15 (s, 3H, *p*-CH_3 of Xyl), 2.23 (s, 3H, *p*-CH_3 of Mes), 2.33 (br s, 6H, *o*-CH_3 of Mes or Xyl), 2.52 (br s, 6H, *o*-CH_3 of Mes or Xyl), 5.09 (m, 1H, CH), 5.49 (dd, J = 10, 5 Hz, 1H, CH), 5.61 (dd, J = 10, 1 Hz, 1H, CH), 5.96 (s, 1H, CH), 6.73 (s, 2H, CH of Mes), 6.77-6.81 (m, 3H, CH), 6.88-6.94 (m, 3H, CH of Xyl); ^{11}B NMR (160.5 MHz, C_6D_6) δ 47 (s), 24 (s); ^{13}C NMR (126 MHz, C_6D_6) δ 18.0 (br, CH_3), 21.23 (CH_3), 21.25 (CH_3), 22.71 (br, CH_3), 22.72 (CH_3), 23.3 (br, CH_3), 24.4 (CH_3 of pin), 83.2 (4° of pin), 108.5 (CH), 120.6 (CH), 121.0 (CH), 122.9 (4°), 124.0 (CH), 127.1 (CH), 128.4 (CH), 128.8 (CH), 129.0 (CH), 135.9 (CH), 136.3 (4°), 136.7 (br, 4°), 138.0 (4°), 138.1 (br, 4°), 141.2 (4°), 141.3 (br, 4°); mp 190.5-193.9 °C (decomp.); Anal. Calcd for $C_{38}H_{48}B_2N_2O_2$: C, 77.83; H, 8.25; N, 4.78; Found: C, 77.90; H, 8.35; N, 4.91.

Estimation of NMR yield for the formation of 5

In a glovebox, a pyridine solution (600 μL) of Xyl-NC (10.5 mg, 79.8 μmol) was added to a pyridine solution (600 μL) of **1** (30.0 mg, 79.8 μmol) in a 15 mL vial at room temperature. After stirring the reaction mixture for 10 min at room temperature, volatiles were removed from the reaction mixture under reduced pressure. A

benzene-d_6 solution (1200 μL) of 1,3,5-trimethoxybenzene (13.4 mg, 79.8 μmol) was added to the residue and the resulting mixture was stirred for 5 min at room temperature. An aliquot (600 μL) of the resulting solution was pipetted to a screw-capped NMR tube. After bringing the NMR tube out from the glovebox, ^{1}H NMR spectrum was recorded to estimate the NMR yield of **2** (82%).

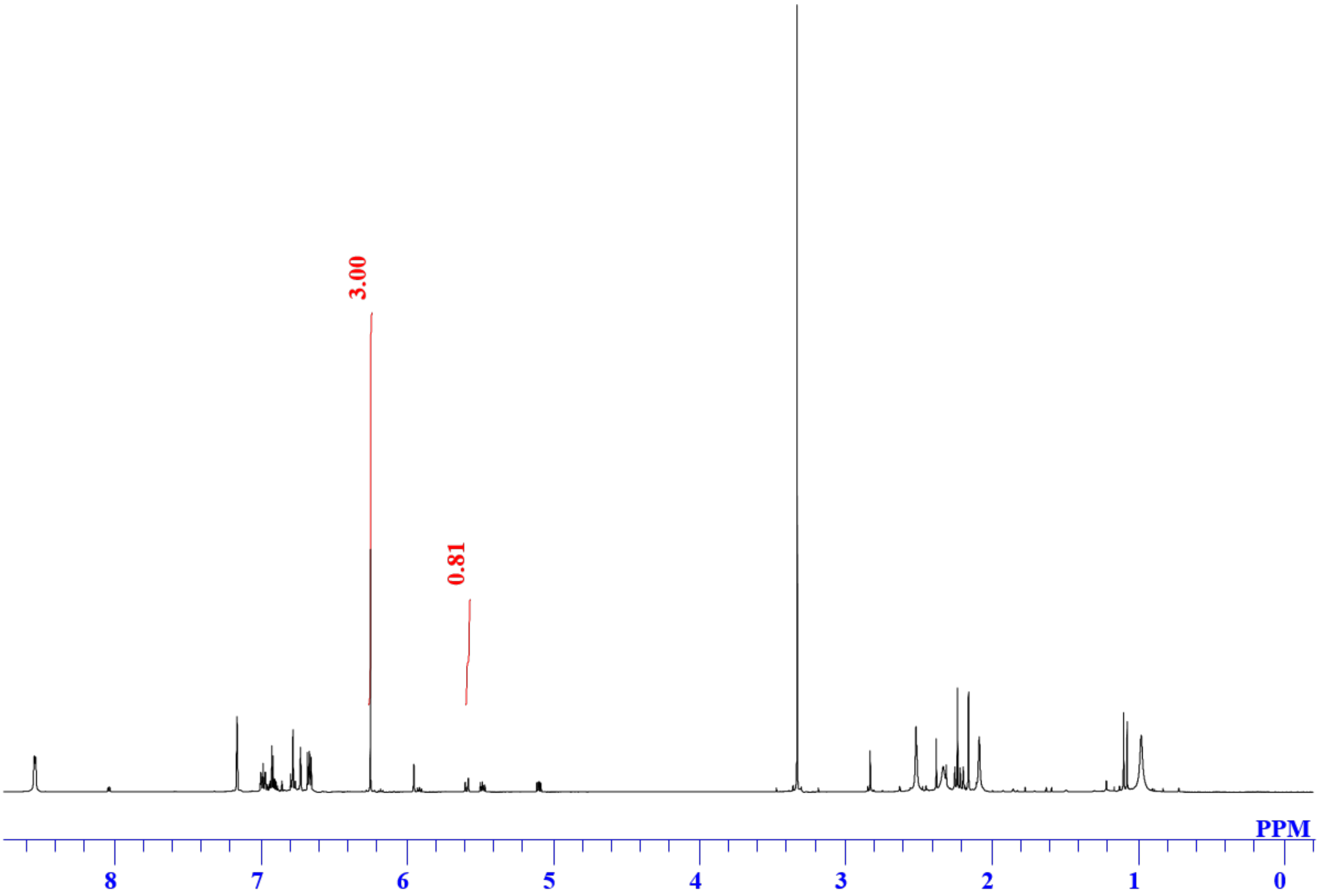

Figure S5. The ^{1}H NMR spectrum of the crude product for the synthesis of **5**

Synthesis of 6

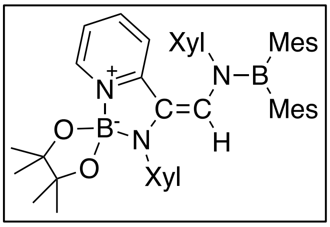

In a glovebox, a pyridine solution (15 mL) of Xyl-NC (349 mg, 2.66 mmol) was added to a pyridine solution (15 mL) of **1** (500 mg, 1.33 mmol) in a 100 mL flask at room temperature. During the reaction mixture was stirred at room temperature for 5 min, the color of the resulting solution turned to be red. Volatiles were removed from the reaction mixture under reduced pressure. The residue was recrystallized from benzene at room temperature to afford yellow crystals of **6** (88.7 mg, 0.124 mmol, 9%). Because of the existence of the equilibrium, signals in the ^{1}H, ^{11}B, and ^{13}C NMR spectrum (^{1}H NMR spectrum at room temperature is shown in Figures S6 and S7) could not be assigned as noted in the main text. However, four low-field signals were exchanged their positions upon cooling the CD_2Cl_2 solution of **6** down to –80 °C (Figure S8). As judged by HH COSY experiment (Figure S9), these four signals were divided to two groups and the blue-colored signal decreased its integral ratio upon cooling (Figure S8). mp 187.9-191.0 °C (decomp.); Anal. Calcd for $C_{47}H_{57}B_2N_3O_2$: C, 78.67; H, 8.01; N, 5.86; Found: C, 78.59; H, 7.90; N, 6.04.

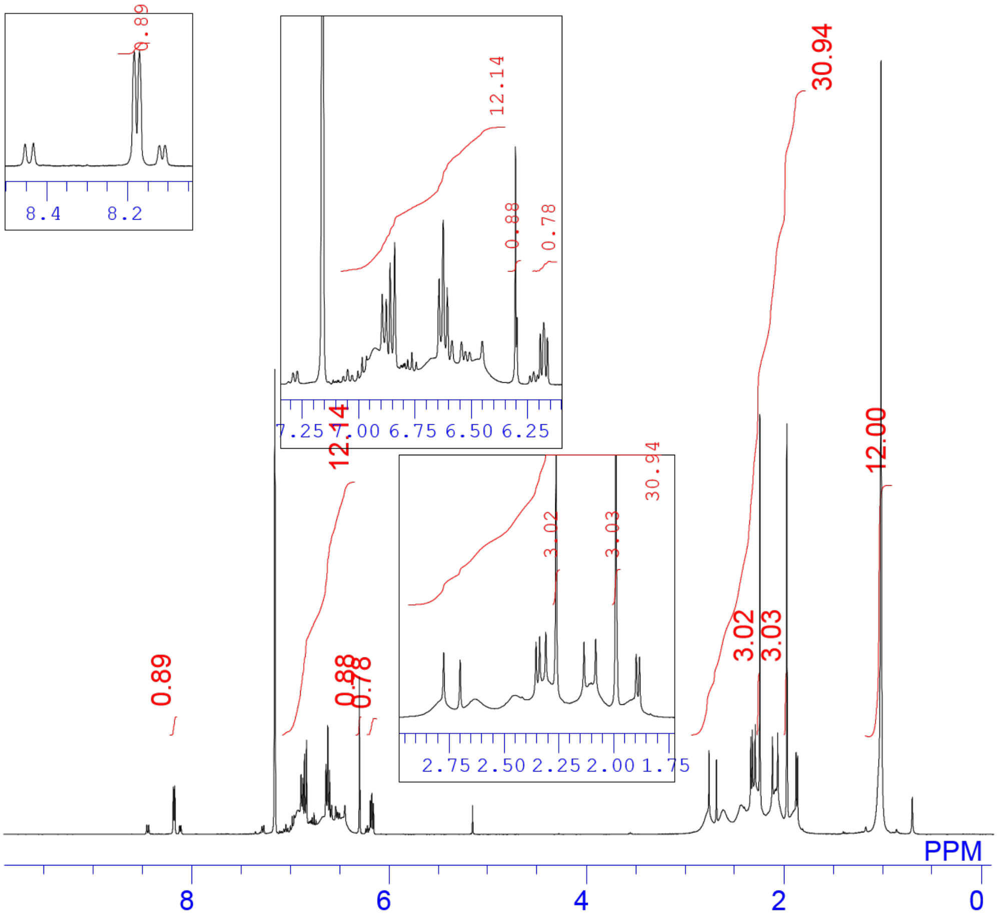

Figure S6. ^1H NMR spectrum of **6** (C_6D_6, RT): The most low-field shifted doublet was used for the estimation of the ^1H NMR yield (see below).

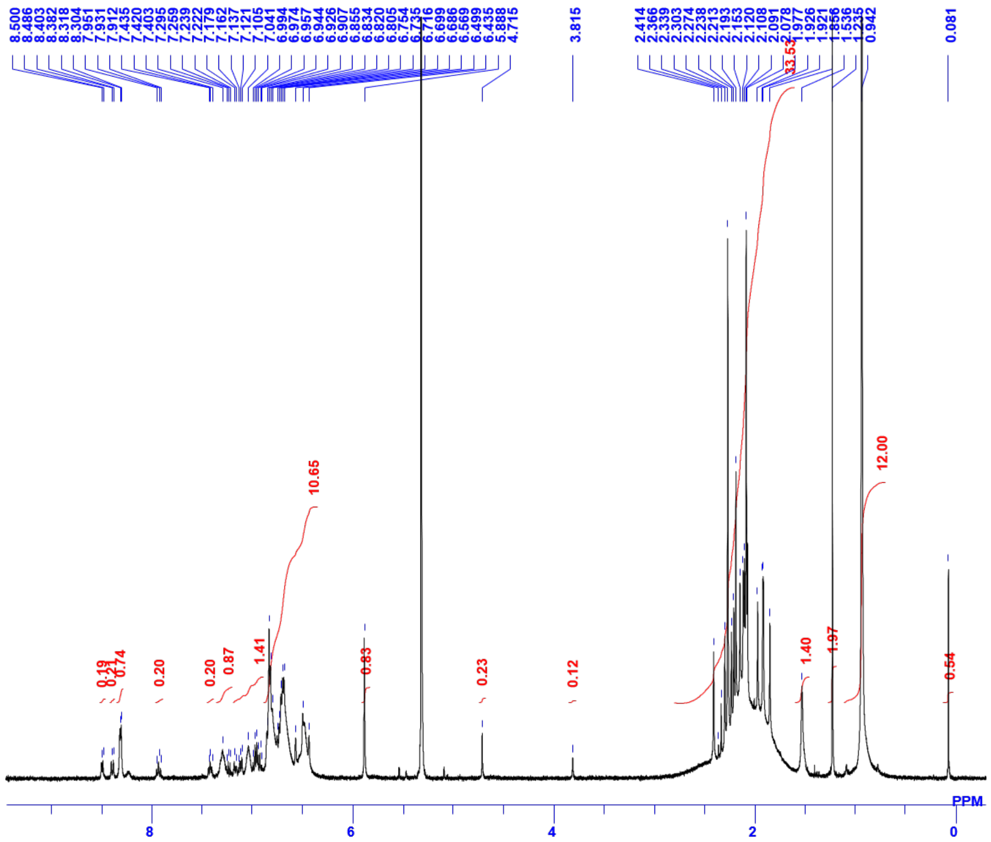

Figure S7. ^1H NMR spectrum of **6** (CD_2Cl_2, RT)

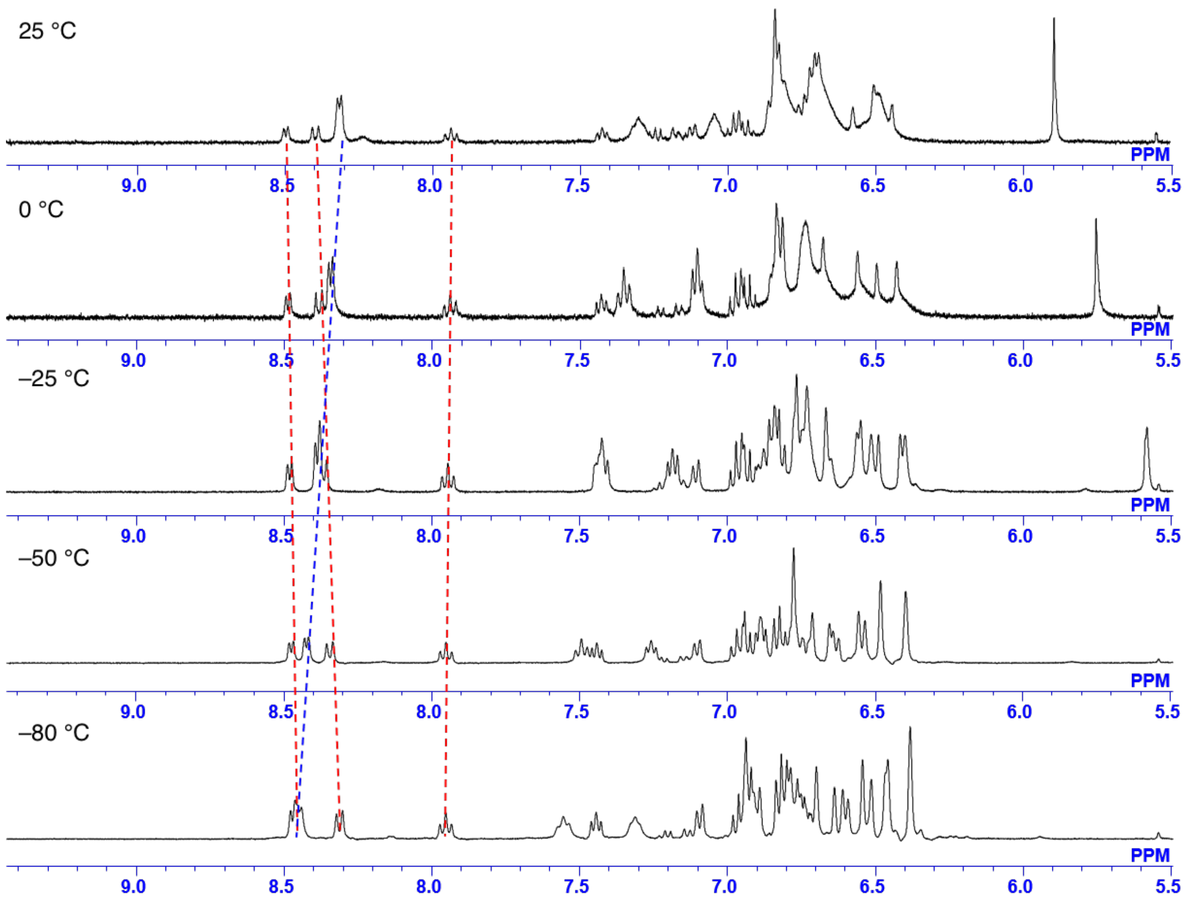

Figure S8. VT ^{1}H NMR spectrum of **6** (CD_2Cl_2) in the range of room temperature to –80 °C; dotted blue line: major isomer at RT, dotted red line: minor isomer at RT

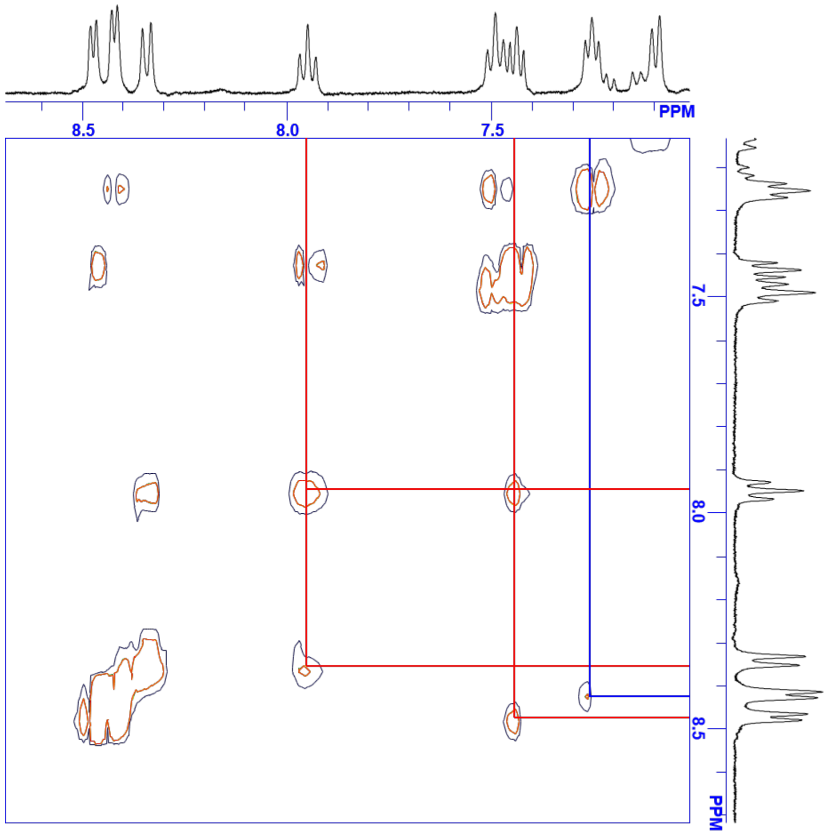

Figure S9. HH COSY spectrum of **6** (CD_2Cl_2) at –50 °C; color of lines for correlation corresponds to the color in Figure S8 (above)

Estimation of NMR yield for the formation of 6

In a glovebox, a pyridine solution (8 mL) of Xyl-NC (21.1 mg, 161 μmol) was added to a pyridine solution (8 mL) of **1** (30.0 mg, 79.8 μmol) in a 30 mL vial at room temperature. After stirring the reaction mixture for 10 min at room temperature, volatiles were removed from the reaction mixture under reduced pressure. A benzene-d_6 solution (600 μL) of 1,3,5-trimethoxybenzene (13.4 mg, 79.8 μmol) was added to the residue and the resulting mixture was stirred for 5 min at room temperature. An aliquot (600 μL) of the resulting solution was pipetted to a screw-capped NMR tube. After bringing the NMR tube out from the glovebox, ^{1}H NMR spectrum was recorded to estimate the NMR yield of **6** (52%).

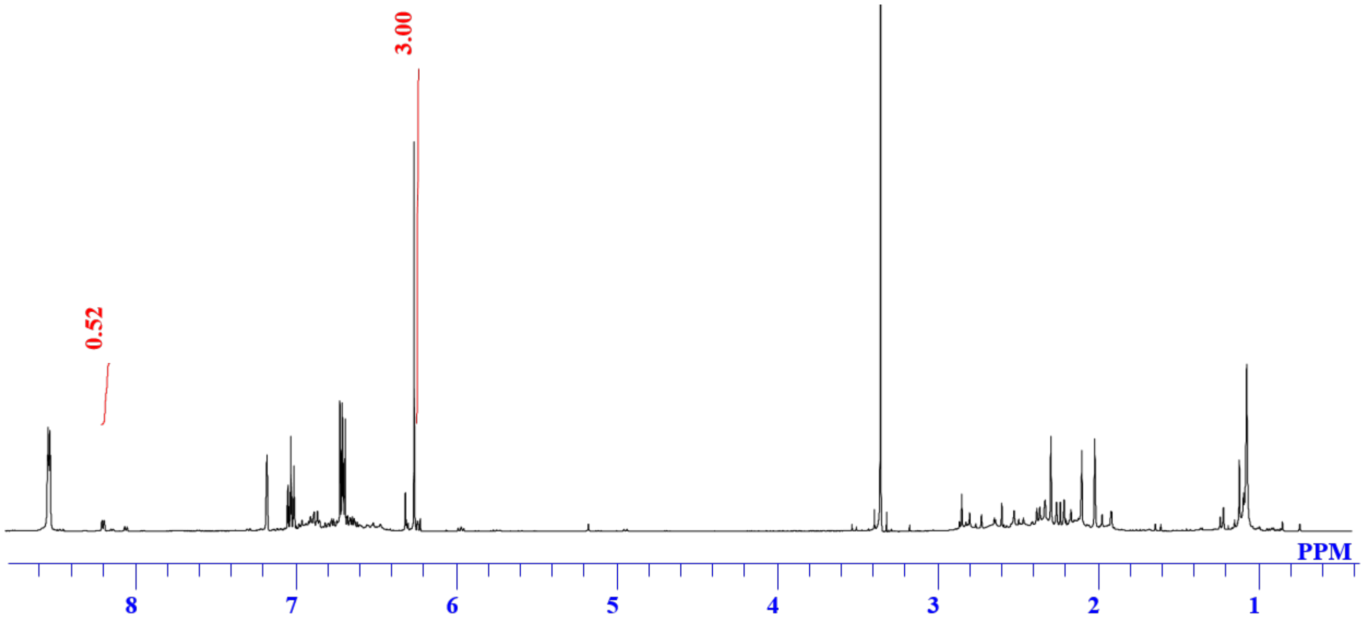

Figure S10. The ^{1}H NMR spectrum of the crude product for the synthesis of **6**

Estimation of NMR yield for the formation of 6 from 5

In a glovebox, a benzene-d_6 solution (200 μL) of Xyl-NC (10.5 mg, 79.8 μmol) was added to a benzene-d_6 solution (800 μL) of **5** (46.8 mg, 79.8 μmol) and in a 15 mL vial at room temperature. After stirring the reaction mixture for 10 min at room temperature, a benzene-d_6 solution (300 μL) of 1,3,5-trimethoxybenzene (13.3 mg, 79.1 μmol) was added to the crude product and the resulting mixture was stirred for 5 min at room temperature. An aliquot (600 μL) of the resulting solution was pipetted to a screw-capped NMR tube. After bringing the NMR tube out from the glovebox, ^{1}H NMR spectrum was recorded to estimate the NMR yield of **6** (99%).

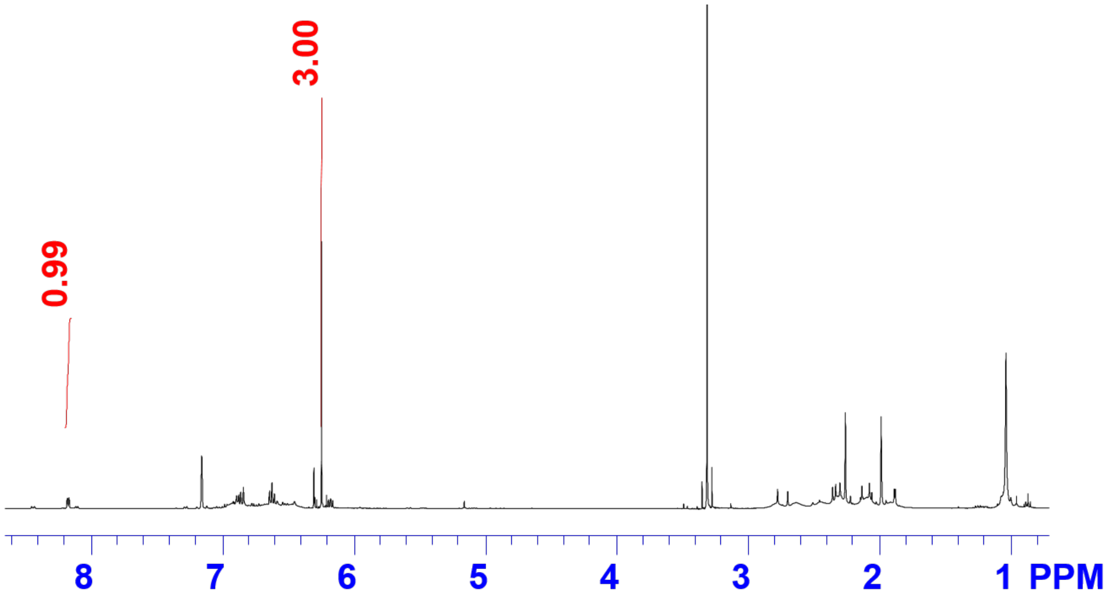

Figure S11. The ^{1}H NMR spectrum of the crude product for the synthesis of **6** from **5**

General procedure for Table 1

In a glovebox, a toluene solution (y/2 mL, y = from Table 1) of Xyl-NC (10.5 mg, 79.8 μmol) was added to a toluene solution (y/2 mL, y = from Table 1) of **1** (30.0 mg, 79.8 μmol) and pyridine (x mL, x = from Table 1) in a vial at room temperature. After stirring the reaction mixture for 10 min at room temperature, volatiles were removed from the reaction mixture under reduced pressure. A benzene-d_6 solution (600 μL) of 1,3,5-trimethoxybenzene (13.4 mg, 79.8 μmol) was added to the residue and the resulting mixture was stirred for 5 min at room temperature. An aliquot (600 μL) of the resulting solution was pipetted to a screw-capped NMR tube. After bringing the NMR tube out from the glovebox, ^{1}H NMR spectrum was recorded to estimate the NMR yield.

General procedure for Table 2

In a glovebox, a toluene solution (y/2 mL, y = from Table 1) of Xyl-NC (21.0 mg, 162 μmol) was added to a toluene solution (y/2 mL, y = from Table 1) of **1** (30.0 mg, 79.8 μmol) and pyridine (x mL, x = from Table 1) in a vial at room temperature. After stirring the reaction mixture for 10 min at room temperature, volatiles were removed from the reaction mixture under reduced pressure. A benzene-d_6 solution (600 μL) of 1,3,5-trimethoxybenzene (13.4 mg, 79.8 μmol) was added to the residue and the resulting mixture was stirred for 5 min at room temperature. An aliquot (600 μL) of the resulting solution was pipetted to a screw-capped NMR tube. After bringing the NMR tube out from the glovebox, ^{1}H NMR spectrum was recorded to estimate the NMR yield.

Synthesis of 7

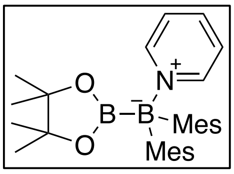

In a glovebox, a pyridine (4 mL, 49.5 mmol) was added to **1** (200 mg, 0.532 mmol) in a 30 mL vial at room temperature. During the reaction mixture was stirred at room temperature for 5 min, the color of the resulting solution turned to be yellow. Volatiles were removed from the reaction mixture under reduced pressure. The residue was recrystallized from hexane (–35 °C) to afford yellow crystals of **7** (190 mg, 0.420 mmol, 78%). ^{1}H NMR (400 MHz, C_6D_6) δ 1.06 (s, 12H, CH_3 of pin), 2.15 (s, 6H, *p*-CH_3 of Mes), 2.39 (s, 12H, *o*-CH_3 of Mes), 6.52-6.65 (br, 2H, 3,5-H of pyridine), 6.79 (s, 4H, CH of Mes), 6.84-6.96 (br, 1H, 4-H of pyridine), 8.53-8.72 (br, 2H, 2,6-H of pyridine); ^{11}B NMR (160.5 MHz, C_6D_6) δ 39 (br s), –0.6 (br s); ^{13}C NMR (126 MHz, C_6D_6) δ 21.1 (*p*-CH_3 of Mes), 24.9 (*o*-CH_3 of Mes), 25.1 (CH_3 of pin), 82.0 (4° of pin), 123.8 (3,5-CH of pyridine), 129.7 (CH of Mes), 135.5 (4°), 137.3 (4-CH of pyridine), 141.6 (4°), 147.3 (4°), 149.8 (2,6-CH of pyridine); mp 118.2-119.7 °C (decomp.); Anal. Calcd for $C_{29}H_{39}B_2NO_2$: C, 76.51; H, 8.64; N, 3.08; Found: C, 76.14; H, 8.39; N, 2.84.

Estimation of NMR yield for the formation of 7

In a glovebox, a pyridine solution (300 μL) was added to **1** (30.0 mg, 79.8 μmol) in a 15 mL vial at room temperature. After stirring the reaction mixture for 10 min at room temperature, volatiles were removed from the reaction mixture under reduced pressure. A benzene-*d*$_6$ solution (600 μL) of 1,3,5-trimethoxybenzene (13.0 mg, 77.3 μmol) was added to the residue and the resulting mixture was stirred for 5 min at room temperature. An aliquot (600 μL) of the resulting solution was pipetted to a screw-capped NMR tube. After bringing the NMR tube out from the glovebox, ^{1}H NMR spectrum was recorded to estimate the NMR yield of **7** (99%).

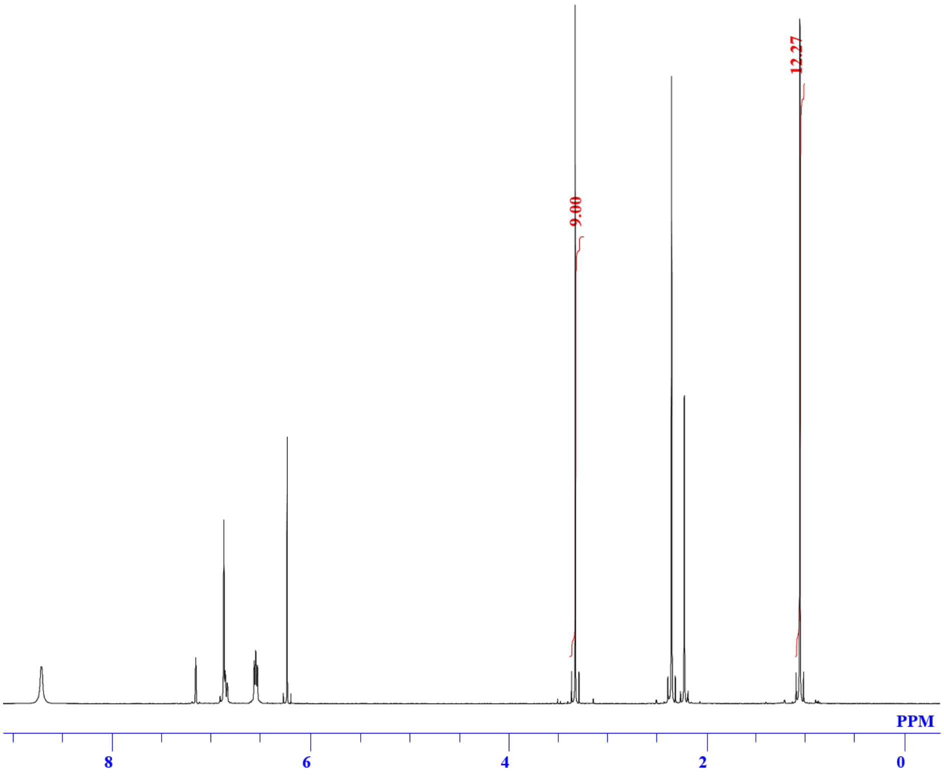

Figure S12. The ^{1}H NMR spectrum of the crude product for the synthesis of **7**

Synthesis of 4a

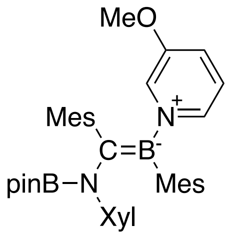

In a glovebox, a toluene solution (3 mL) of Xyl-NC (105 mg, 0.798 mmol) was added to a toluene solution (6 mL) of **1** (300 mg, 0.798 mmol) and 3-methoxypyridine (0.4 mL, 3.99 mmol) in a 30 mL vial at room temperature. During the reaction mixture was stirred at room temperature for 5 min, the color of the resulting solution turned to be blue. Volatiles were removed from the reaction mixture under reduced pressure. The residue was recrystallized from hexane (–35 °C) to afford blue crystals of **4a** (276 mg, 0.448 mmol, 56%). ^{1}H NMR (400 MHz, C_6D_6) δ 1.07 (s, 12H, CH_3 of pin), 2.14 (s, 3H, *p*-CH_3 of Mes), 2.17 (s, 6H, *o*-CH_3 of Xyl or Mes), 2.30 (s, 3H, *p*-CH_3 of Mes), 2.48 (s, 6H, *o*-CH_3 of Xyl or Mes), 2.84 (s, 6H, *o*-CH_3 of Xyl or Mes), 2.87 (s, 3H, OCH_3), 5.96 (dd, *J* = 8, 6 Hz, 1H, 5-CH of pyridine), 6.11 (ddd, *J* = 8, 3, 1 Hz, 1H, 4-CH of pyridine), 6.73 (s, 2H, CH of Mes), 6.86 (dd, *J* = 7, 7 Hz, 1H, 4-CH of Xyl), 6.91 (d, *J* = 7 Hz, 2H, 3,5-CH of Xyl), 7.00 (s, 2H, CH of Mes), 7.69 (d, *J* = 6 Hz, 1H, 6-CH of pyridine), 7.99 (d, *J* = 3 Hz, 1H, 2-CH of pyridine); ^{11}B NMR (160.5 MHz, C_6D_6) δ 29 (s), 23 (s); Similar to the case of **4**, decomposition of **4a** in solution at room temperature prevented us to perform complete characterization with ^{13}C NMR spectrum; mp 91.9-92.7 °C (decomp.); Anal. Calcd for $C_{39}H_{50}B_2N_2O_2$: C, 75.99; H, 8.18; N, 4.54; Found: C, 75.60; H, 8.28; N, 4.14.

Estimation of NMR yield for the formation of 4a with 1 equivalent of Xyl-NC

In a glovebox, a toluene solution (300 μL) of Xyl-NC (10.5 mg, 79.8 μmol) was added to a toluene solution (260 μL) of **1** (30.0 mg, 79.8 μmol) and 3-methoxypyridine (40.3 μL, 399 μmol) in a 15 mL vial at room temperature. After stirring the reaction mixture for 10 min at room temperature, volatiles were removed from the reaction mixture under reduced pressure. A benzene-*d*$_6$ solution (600 μL) of 1,3,5-trimethoxybenzene (13.4 mg, 79.8 μmol) was added to the crude product and the resulting mixture was stirred for 5 min at room temperature. An aliquot (600 μL) of the resulting solution was pipetted to a screw-capped NMR tube. After bringing the NMR tube out from the glovebox, ^{1}H NMR spectra was recorded to estimate the NMR yield of **4a** (65%).

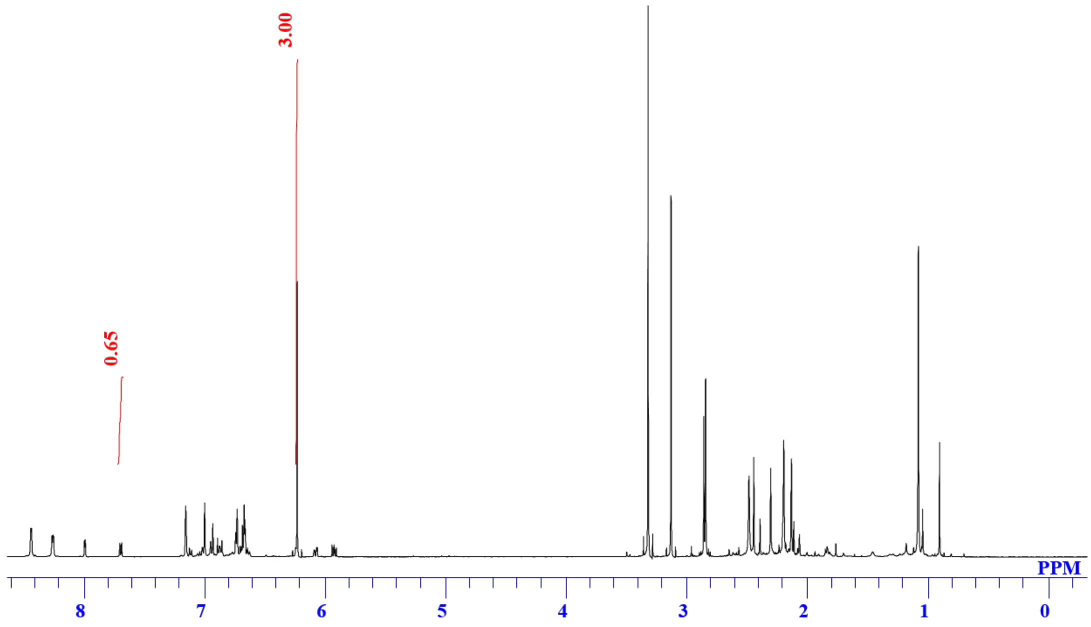

Figure S13. The ^{1}H NMR spectrum of the crude product for the synthesis of **4a**

Estimation of NMR yield for the formation of 4a with 2 equivalent of Xyl-NC

In a glovebox, a toluene solution (300 μL) of Xyl-NC (21.0 mg, 160 μmol) was added to a toluene solution (260 μL) of **1** (30.0 mg, 79.8 μmol) and 3-methoxypyridine (40.3 μL, 399 μmol) in a 15 mL vial at room temperature. After stirring the reaction mixture for 10 min at room temperature, volatiles were removed from the reaction mixture under reduced pressure. A benzene-d_6 solution (600 μL) of 1,3,5-trimethoxybenzene (13.3 mg, 79.1 μmol) was added to the residue and the resulting mixture was stirred for 5 min at room temperature. An aliquot (600 μL) of the resulting solution was pipetted to a screw-capped NMR tube. After bringing the NMR tube out from the glovebox, ^{1}H NMR spectrum was recorded to estimate the NMR yield of **2** (44%) and **4a** (18%).

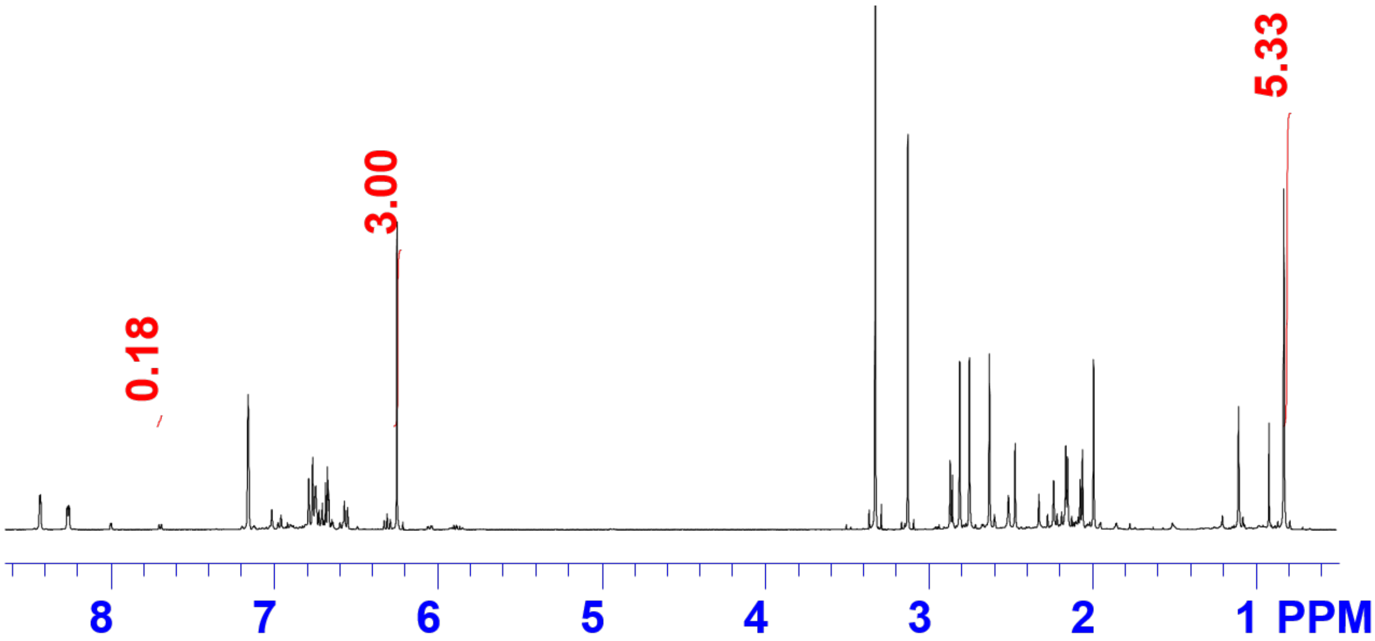

Figure S14. The ^{1}H NMR spectrum of the crude product for the reaction of **1** with 2 eq. Xyl-NC and 3-methoxypyridine

Synthesis of 4b

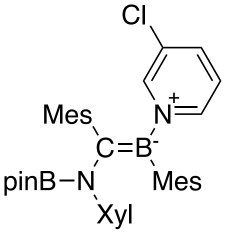

In a glovebox, a benzene-d_6 solution (300 μL) of Xyl-NC (10.5 mg, 79.8 μmol) was added to a benzene-d_6 solution (300 μL) of **1** (30.0 mg, 79.8 μmol) and 3-chloropyridine (37.5 μL, 399 μmol) in a 15 mL vial at room temperature. During the reaction mixture was stirred at room temperature for 5 min, the color of the resulting solution turned to be blue. An aliquot (600 μL) of the resulting solution was pipetted to a screw-capped NMR tube. After bringing the NMR tube out from the glovebox, ^{1}H, ^{11}B, and ^{13}C NMR spectra of the crude product for the synthesis of **4b** was recorded. Since **4b** decomposed upon evaporation of solvent, all the effort to isolate **4b** was failed. Therefore, we are providing only NMR spectroscopic data for **4b**. A few single crystals suitable for X-ray analysis were obtained by recrystallization (–35 °C) of crude product from a reaction with 3-chloropyridine as a solvent. ^{1}H NMR (400 MHz, C_6D_6) δ 1.01 (s, 12H, CH_3 of pin), 2.19 (s, 3H, *p*-CH_3 of

Mes), 2.24 (s, 6H, *o*-CH_3 of Xyl or Mes), 2.26 (s, 3H, *p*-CH_3 of Mes), 2.48 (s; 6H, *o*-CH_3 of Xyl or Mes), 2.72 (s, 6H, *o*-CH_3 of Xyl or Mes), 5.67 (dd, J = 7, 6 Hz, 1H, 3-CH of pyridine), 6.17 (d, J = 7 Hz, 1H, 4-CH of pyridine), 6.77 (s, 2H, CH of Mes), 6.82 (t, J = 8 Hz, 1H, 4-CH of Xyl), 6.86 (d, J = 8 Hz, 2H, 3,5-CH of Xyl), 6.91 (s, 2H, CH of Mes), 7.70 (d, J = 6 Hz, 1H, 2-CH of pyridine), 8.22 (s, 1H, 6-CH of pyridine); ^{11}B NMR (160.5 MHz, C_6D_6) δ 28 (s), 24 (s); Similar to the case of **4**, decomposition of **4a** in solution at room temperature prevented us to perform complete characterization with ^{13}C NMR spectrum.

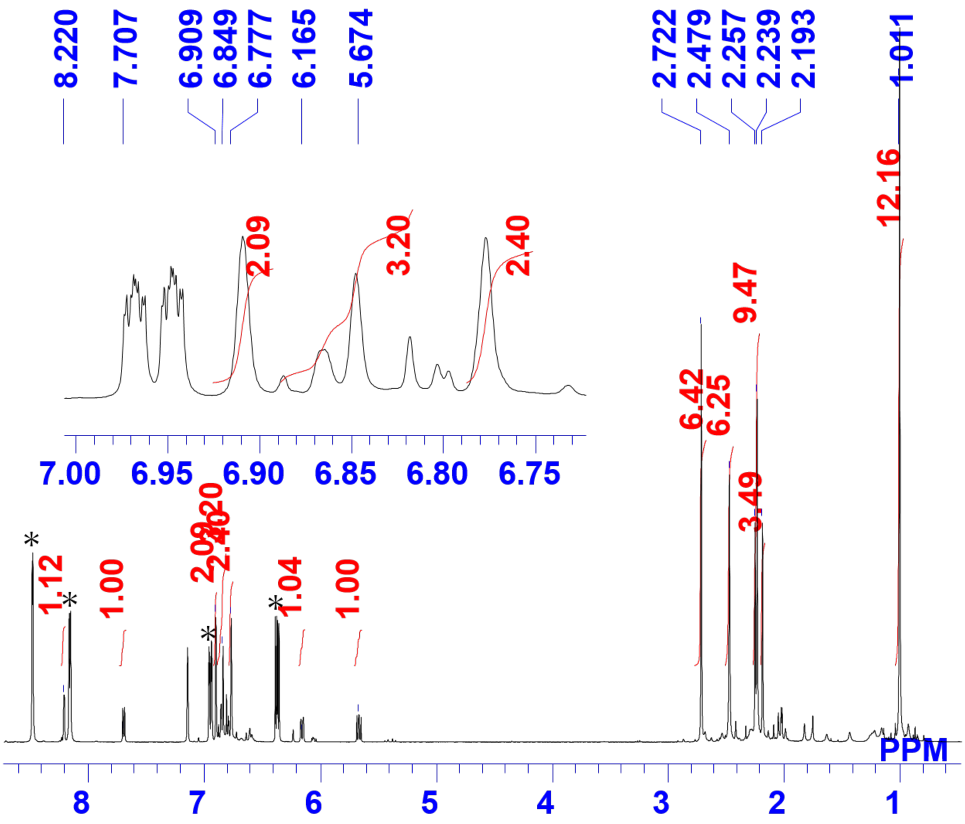

Figure S15. The ^{1}H NMR spectrum(benzene-d_6) of the crude product for the synthesis of **4b** (asterisks denoted signals of 3-chloropyridine)

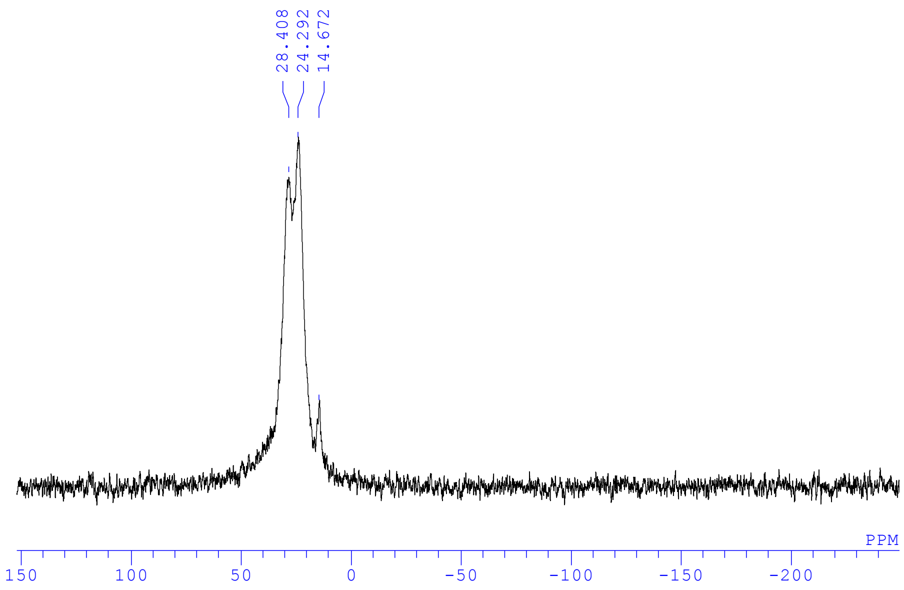

Figure S16. The ^{11}B NMR spectrum (benzene-d_6) of the crude product for the synthesis of **4b**

Estimation of NMR yield for the formation of 4b

In a glovebox, a benzene-d_6 solution (300 μL) of Xyl-NC (10.5 mg, 79.8 μmol) was added to a benzene-d_6 solution (260 μL) of **1** (30.0 mg, 79.8 μmol) and 3-chloropyridine (37.4 μL, 399 μmol) in a 15 mL vial at room temperature. After stirring the reaction mixture for 10 min at room temperature, a benzene-d_6 solution (600 μL) of 1,3,5-trimethoxybenzene (13.5 mg, 80.3 μmol) was added to the reaction mixture and the resulting mixture was stirred for 5 min at room temperature. An aliquot (600 μL) of the resulting solution was pipetted to a screw-capped NMR tube. After bringing the NMR tube out from the glovebox, ^{1}H NMR spectrum was recorded to estimate the NMR yield of **4b** (76%).

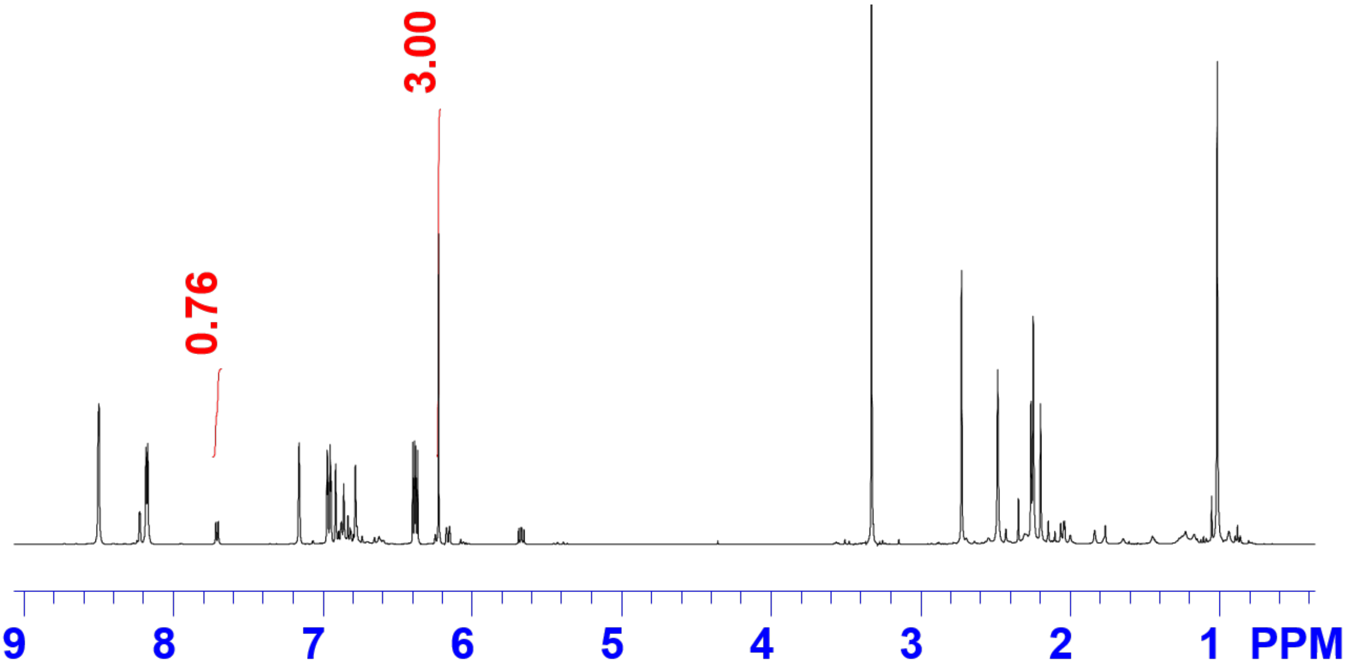

Figure S17. The ^{1}H NMR spectrum of the crude product for the synthesis of **4b**

Estimation of NMR yield for the formation of 4b with 2 equivalent of Xyl-NC

In a glovebox, a toluene solution (300 μL) of Xyl-NC (21.0 mg, 160 μmol) was added to a toluene solution (300 μL) of **1** (30.0 mg, 79.8 μmol) and 3-chloropyridine (37.4 μL, 399 μmol) in a 15 mL vial at room temperature. After stirring the reaction mixture for 10 min at room temperature, volatiles were removed from the reaction mixture under reduced pressure. A benzene-d_6 solution (600 μL) of 1,3,5-trimethoxybenzene (13.5 mg, 80.3 μmol) was added to the residue and the resulting mixture was stirred for 5 min at room temperature. An aliquot (600 μL) of the resulting solution was pipetted to a screw-capped NMR tube. After bringing the NMR tube out from the glovebox, ^{1}H NMR spectrum was recorded to estimate the NMR yield of **2** (66%) and **4b** (26%).

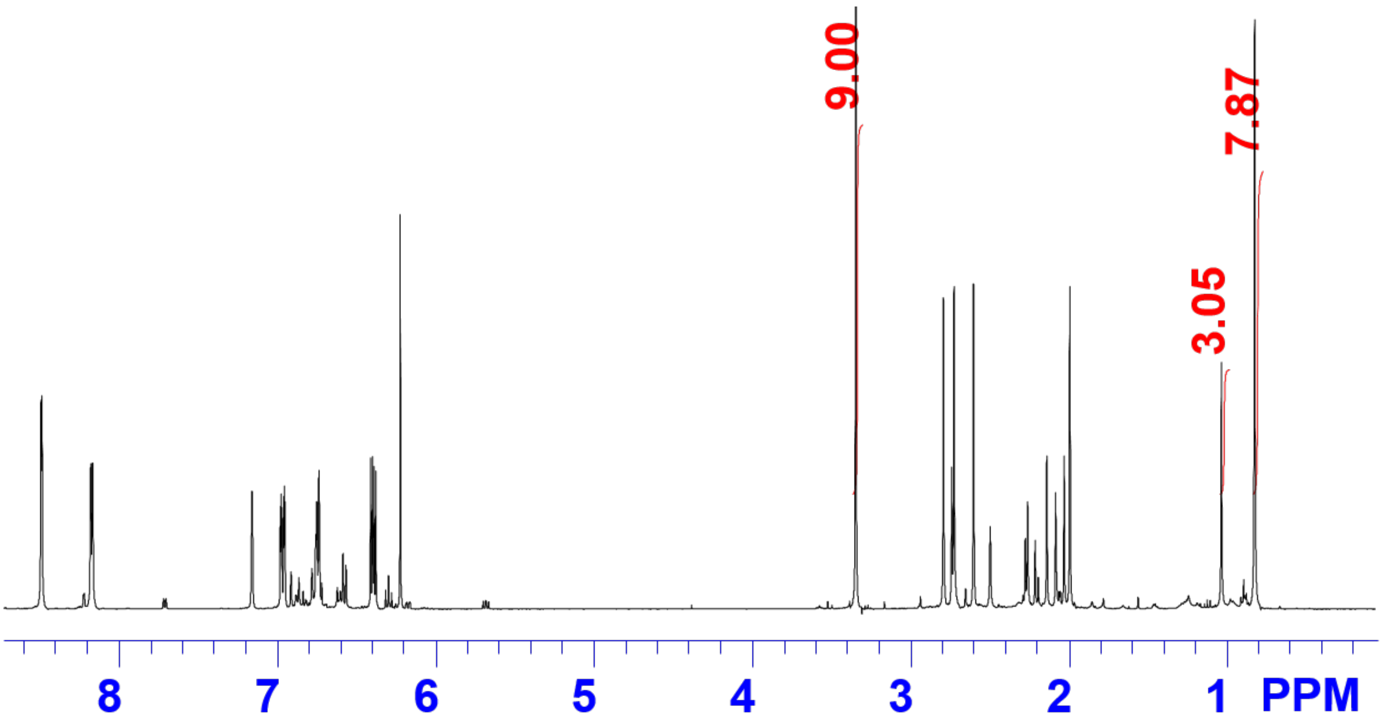

Figure S18. The ^{1}H NMR spectrum of the crude product for the reaction of **1** with 2 eq. Xyl-NC and 3-chloropyridine

Synthesis of 4c

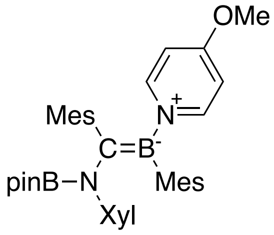

In a glovebox, a toluene solution (0.9 mL) of Xyl-NC (36.9 mg, 0.281 mmol) was added to a toluene solution (0.9 mL) of **1** (106 mg, 0.281 mmol) and 4-methoxypyridine (30.0 μL, 0.281 mmol) in a 15 mL vial at room temperature. During the reaction mixture was stirred at room temperature for 5 min, the color of the resulting solution turned to be red. Volatiles were removed from the reaction mixture under reduced pressure. The residue washed with hexane (–35 °C) to afford red solids of **4c** (45.5 mg, 0.0738 mmol, 26%). ^{1}H NMR (400 MHz, C_6D_6), δ 1.13 (s, 12H, CH_3 of pin), 2.18 (s, 3H, *p*-CH_3 of Mes), 2.23 (s, 6H *o*-CH_3 of Xyl or Mes), 2.34 (s, 3H, *p*-CH_3 of Mes), 2.48 (s, 3H, OCH_3), 2.57 (s, 6H, *o*-CH_3 of Xyl or Mes), 2.92 (s, 6H, *o*-CH_3 of Xyl or Mes), 5.48 (d, J = 7 Hz, 2H, 3-CH of pyridine), 6.82 (s, 2H, CH of Mes), 6.94 (t, J = 6 Hz, 1H, 4-CH of Xyl), 7.02 (d, J = 6 Hz, 2H, 3,5-CH of Xyl), 7.07 (s, 2H, CH of Mes), 7.94 (d, J = 7 Hz, 2H, 2,6-CH of pyridine); ^{11}B NMR (160.5 MHz, C_6D_6) δ 29 (s), 24 (s); Similar to the case of **4**, decomposition of **4c** in solution at room temperature prevented us to perform complete characterization with ^{13}C NMR spectrum; mp 72.3-75.9 °C (decomp.); Anal. Calcd for $C_{39}H_{50}B_2N_2O_3$: C, 75.99; H, 8.18; N, 4.54; Found: C, 76.15; H, 8.24; N, 4.82.

Estimation of NMR yield for the formation of 4c

In a glovebox, a toluene solution (300 μL) of Xyl-NC (10.5 mg, 79.8 μmol) was added to a toluene solution (260 μL) of **1** (30.0 mg, 79.8 μmol) and 4-methoxypyridine (40.3 μL, 399 μmol) in a 15 mL vial at room temperature. After stirring the reaction mixture for 10 min at room temperature, a benzene-d_6 solution (600 μL) of 1,3,5-trimethoxybenzene (13.4 mg, 79.8 μmol) was added to the crude product and the resulting

mixture was stirred for 5 min at room temperature. An aliquot (600 μL) of the resulting solution was pipetted to a screw-capped NMR tube. After bringing the NMR tube out from the glovebox, ^{1}H NMR spectra was recorded to estimate the NMR yield of **4c** (80%).

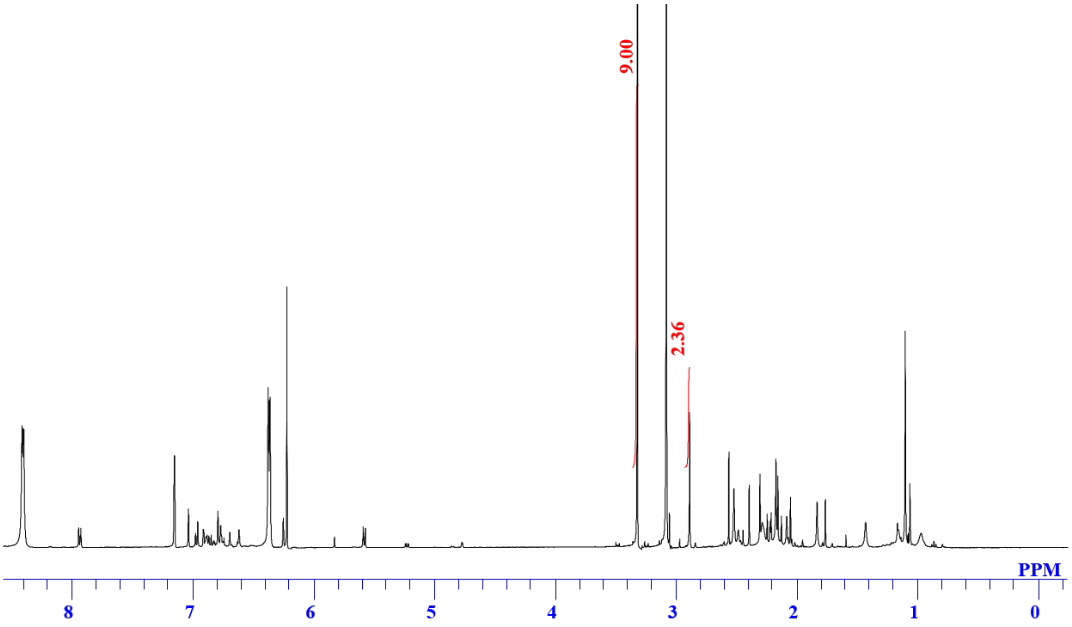

Figure S19. The ^{1}H NMR spectrum of the crude product for the synthesis of **4c**

Estimation of NMR yield for the formation of 4c with 2 equivalent of Xyl-NC

In a glovebox, a toluene solution (300 μL) of Xyl-NC (21.0 mg, 160 μmol) was added to a toluene solution (300 μL) of **1** (30.0 mg, 79.8 μmol) and 4-methoxypyridine (42.5 μL, 399 μmol) in a 15 mL vial at room temperature. After stirring the reaction mixture for 10 min at room temperature, volatiles were removed from the reaction mixture under reduced pressure. A benzene-d_6 solution (600 μL) of 1,3,5-trimethoxybenzene (13.5 mg, 80.3 μmol) was added to the residue and the resulting mixture was stirred for 5 min at room temperature. An aliquot (600 μL) of the resulting solution was pipetted to a screw-capped NMR tube. After bringing the NMR tube out from the glovebox, ^{1}H NMR spectrum was recorded to estimate the NMR yield of **2** (50%) and **4a** (39%).

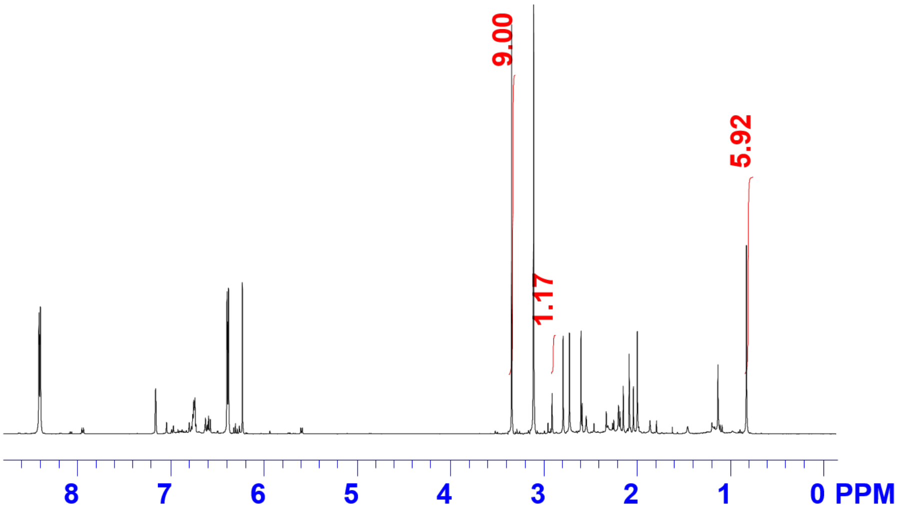

Figure S20. The ^{1}H NMR spectrum of the crude product for the reaction of **1** with 2 eq. Xyl-NC and 4-methoxypyridine

Synthesis of 5a

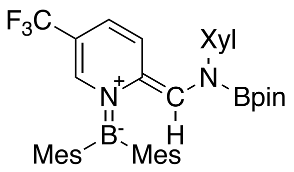

In a glovebox, a toluene solution (3 mL) of Xyl-NC (105 mg, 0.798 mmol) was added to a toluene solution (2.54 mL) of **1** (300 mg, 0.798 mmol) and 3-trifluoromethylpyridine (0.460 mL, 3.99 mmol) in a 15 mL vial at room temperature. During the reaction mixture was stirred at room temperature for 5 min, the color of the resulting solution turned to be green. Volatiles were removed from the reaction mixture under reduced pressure. The residue was recrystallized from pentane at room temperature to afford yellow crystals of **5a** (340 mg, 0.519 mmol, 65%). Single crystals suitable for X-ray diffraction analysis were obtained by recrystallization from hexane (–35 °C). ^{1}H NMR (400 MHz, C_6D_6) δ 0.94 (br s, 12H, CH_3 of pin), 2.00 (br s, 6H, *o*-CH_3 of Xyl or Mes), 2.06 (s, 3H, *p*-CH_3 of Mes), 2.19 (s, 3H, *p*-CH_3 of Mes), 2.24 (br s, 6H, *o*-CH_3 of Xyl or Mes), 2.48 (s, 6H, *o*-CH_3 of Xyl or Mes), 5.43 (d, J = 10 Hz, 1H, CH), 5.57 (d, J = 10 Hz, 1H, CH), 6.08 (s, 1H, CH), 6.64 (s, 2H, CH of Mes), 6.73 (s, 2H, CH of Mes), 6.91 (s, 3H) 7.50 (s, 1H); ^{11}B NMR (160.5 MHz, C_6D_6) δ 50 (s), 23 (s); ^{13}C NMR (126 MHz, C_6D_6) δ 17.9 (CH_3), 21.2 (CH_3), 22.6 (CH_3), 23.1 (CH_3), 24.3 (CH_3 of pin), 83.5 (4° of pin), 112.8 (q, $^{2}J_{FC}$ = 33 Hz, 4°), 115.45 (CH), 115.47 (CH), 120.0 (4°), 122.1 (CH), 124.9 (q, $^{1}J_{FC}$ = 272 Hz, CF_3), 126.3 (CH), 127.5 (CH), 127.9 (4°), 128.5 (CH), 128.9 (CH), 129.3 (CH), 136.5 (br, 4°), 137.0 (4°), 137.4 (q, $^{3}J_{FC}$ = 3 Hz, CH), 137.8 (br, 4°), 139.0 (4°), 140.5 (4°), 141.2 (br, 4°); mp 174.4-177.8 °C (decomp.); Anal. Calcd for $C_{39}H_{47}B_2F_3N_2O_2$: C, 71.58; H, 7.24; N, 4.28; Found: C, 71.63; H, 7.16; N, 4.17.

Estimation of NMR yield for the formation of 5a

In a glovebox, a toluene solution (300 μL) of Xyl-NC (10.7 mg, 80.3 μmol) was added to a toluene solution (300 μL) of **1** (30.0 mg, 79.8 μmol) and 3-trifluoromethylpyridine (45.9 μL, 399 μmol) in a 15 mL vial at room temperature. After stirring the reaction mixture for 10 min at room temperature, volatiles were removed from the reaction mixture under reduced pressure. A benzene-*d*$_6$ solution (600 μL) of 1,3,5-trimethoxybenzene (13.3 mg, 79.1 μmol) was added to the residue and the resulting mixture was stirred for 5 min at room temperature. An aliquot (600 μL) of the resulting solution was pipetted to a screw-capped NMR tube. After bringing the NMR tube out from the glovebox, ^{1}H NMR spectrum was recorded to estimate the NMR yield of **5a** (79%).

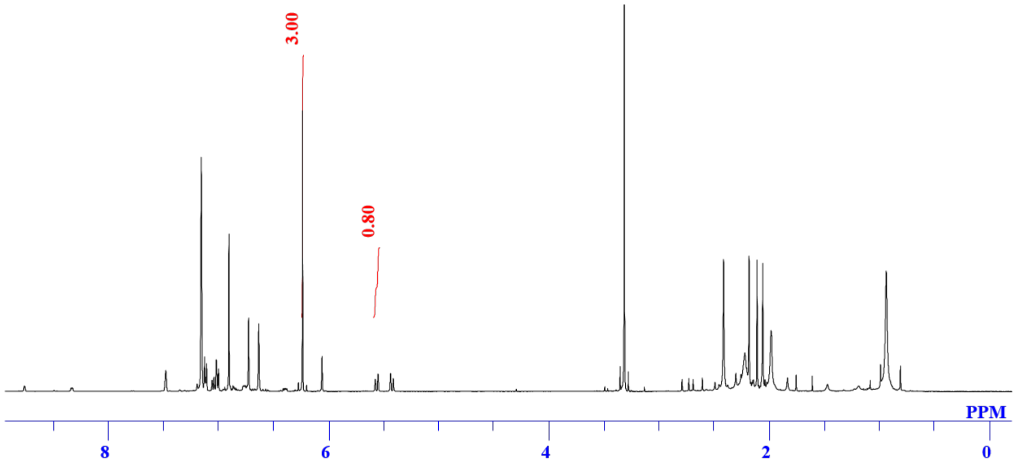

Figure S21. The ^{1}H NMR spectrum of the crude product for the synthesis of **5a**

Synthesis of 5b

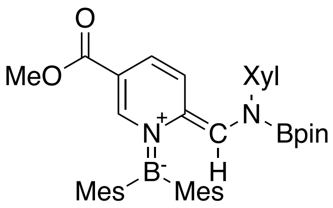

In a glovebox, a toluene solution (0.9 mL) of Xyl-NC (105 mg, 0.798 mmol) was added to a pyridine solution (2.7 mL) of **1** (300 mg, 0.798 mmol) and 3-(methoxycarbonyl)pyridine (546 mg, 3.99 mmol) in a 30 mL vial at room temperature. During the reaction mixture was stirred at room temperature for 5 min, the color of the resulting solution turned to be red. Volatiles were removed from the reaction mixture under reduced pressure. The residue was recrystallized from hexane (–35 °C) to afford yellow crystals of **5b** (159 mg, 0.247 mmol, 31%). ^{1}H NMR (400 MHz, C_6D_6) δ 0.95 (br s, 12H, CH_3 of pin), 2.04 (br s, 6H, *o*-CH_3 of Xyl or Mes), 2.08 (s, 3H, *p*-CH_3 of Mes), 2.20 (s, 3H, *p*-CH_3 of Mes), 2.28 (br s, 6H, *o*-CH_3 of Xyl or Mes), 2.44 (br s, 6H, *o*-CH_3 of Xyl or Mes), 3.13 (s, 3H, OCH_3), 5.51 (d, J = 10 Hz, 1H, CH), 6.03 (t, J = 1 Hz, 1H, CH), 6.41 (dt, J = 10, 1 Hz, 1H, CH), 6.66 (s, 2H, CH of Mes), 6.75 (s, 2H, CH of Mes), 6.88-6.93 (br m, 3H, CH of Xyl), 8.07 (dd, J = 2, 1 Hz, 1H, CH); ^{11}B NMR (160.5 MHz, C_6D_6) δ 52 (s), 24 (s); ^{13}C NMR (126 MHz, C_6D_6) δ 17.9 (CH_3), 21.2 (CH_3), 22.8 (br, CH_3), 23.2 (br, CH_3), 24.4 (CH_3 of pin), 50.8 (OCH_3), 83.3 (4° of pin), 113.5 (4°), 119.3 (CH), 119.9 (CH), 121.2 (4°), 124.9 (CH), 127.4 (CH), 128.5 (CH), 128.6, (CH), 128.9 (CH), 129.3 (CH), 136.5 (4°), 136.9 (br, 4°), 137.1 (4°), 138.2 (br, 4°), 139.0 (4°), 140.7 (4°), 141.4 (br, 4°), 144.8 (CH), 165.8 (C=O); mp 181.8-184.9 °C (decomp.); Anal. Calcd for $C_{40}H_{50}B_2N_2O_4$: C, 74.55; H, 7.81; N, 4.35; Found: C, 74.11; H, 8.17; N, 4.25.

Estimation of NMR yield for the formation of 5b

In a glovebox, a toluene solution (300 μL) of Xyl-NC (10.5 mg, 79.8 μmol) was added to a toluene solution (300 μL) of **1** (30.0 mg, 79.8 μmol) and 3-(methoxycarbonyl) pyridine (54.0 mg, 39.9 μmol) in a 15 mL vial at room temperature. After stirring the reaction mixture for 10 min at room temperature, volatiles were removed from the reaction mixture under reduced pressure. A benzene-d_6 solution (600 μL) of 1,3,5-trimethoxybenzene (13.6 mg, 80.9 μmol) was added to the residue and the resulting mixture was stirred for 5 min at room temperature. An aliquot (600 μL) of the resulting solution was pipetted to a screw-capped NMR tube. After bringing the NMR tube out from the glovebox, ^{1}H NMR spectrum was recorded to estimate the NMR yield of **5b** (67%).

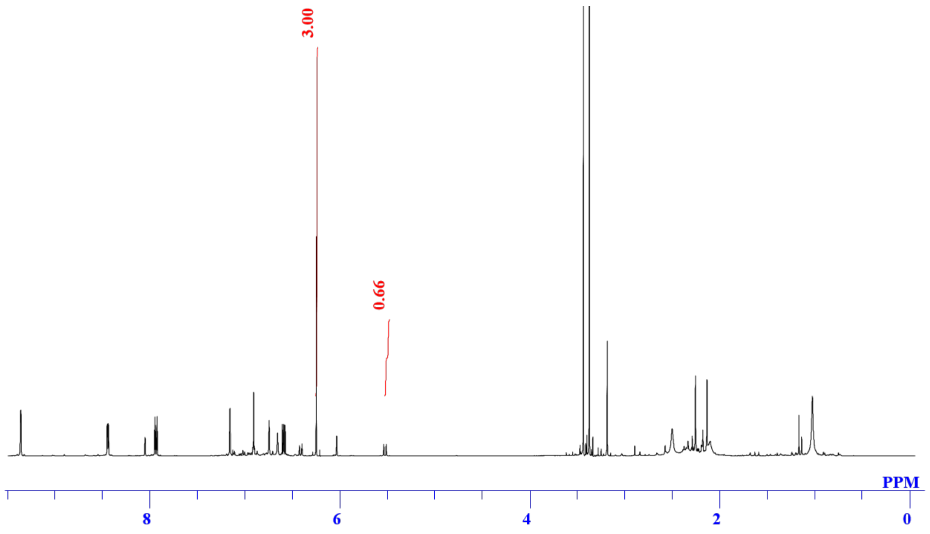

Figure S22. The ^{1}H NMR spectrum of the crude product for the synthesis of **5b**

Synthesis of 5c

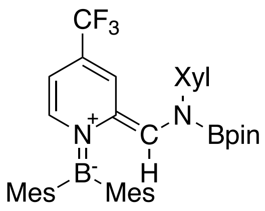

In a glovebox, a toluene solution (2.5 mL) of Xyl-NC (174 mg, 1.33 mmol) was added to a toluene solution (2.5 mL) of **1** (500 mg, 1.33 mmol) and 4-trifluoromethylpyridine (770 μL) in a 30 mL vial at room temperature. During the reaction mixture was stirred at room temperature for 5 min, the color of the resulting solution turned to be green. Volatiles were removed from the reaction mixture under reduced pressure. The residue was recrystallized from pentane at room temperature to afford yellow crystals of **5c** (276 mg, 0.422 mmol, 32%). Single crystals suitable for X-ray diffraction analysis were obtained by recrystallization from hexane (–35 °C). ^{1}H NMR (400 MHz, C_6D_6) δ 0.93 (br s, 12H, CH_3 of pin), 1.97 (br s, 6H, *o*-CH_3 of Mes or Xyl), 2.15 (s, 3H, *p*-CH_3 of Mes), 2.18 (s, 3H, *p*-CH_3 of Mes), 2.27 (br s, 6H, *o*-CH_3 of Mes or Xyl), 2.41 (br s, 6H, *o*-CH_3 of Mes or Xyl), 5.26 (dd, J = 8, 1 Hz, 1H, CH of pyridine), 5.88 (br s, 1H, CH), 6.14 (s, 1H, CH), 6.69-6.80 (m, 5H, CH of Mes and pyridine), 6.91 (br s, 3H, CH of Xyl); ^{11}B NMR (160.5 MHz, C_6D_6) δ 49 (s), 24 (s); ^{13}C NMR (126 MHz, C_6D_6, two B-bonded carbon atoms were not detected) δ 17.7 (CH_3), 21.18 (CH_3), 21.20 (CH_3), 22.6 (CH_3), 23.1 (CH_3), 24.3 (CH_3 of pin), 83.6 (4° of pin), 103.2 (CH), 119.3 (4°), 121.6 (q, $^{3}J_{FC}$ = 4 Hz, CH), 122.6 (q, $^{2}J_{FC}$ = 32 Hz, 4°), 124.1 (q, $^{1}J_{FC}$ = 272 Hz, CF_3), 127.9 (CH), 128.7 (CH), 128.9 (CH), 129.1 (CH), 136.2 (4°), 136.8 (4°), 137.3 (4°), 137.9 (CH), 138.0 (4°), 138.5 (4°), 140.2 (4°), 141.2 (4°); mp 178.0-181.5 °C (decomp.); Anal. Calcd for $C_{39}H_{47}B_2F_3N_2O_2$: C, 71.58; H, 7.24; N, 4.28; Found: C, 71.38; H, 7.16; N, 4.08.

Estimation of NMR yield for the formation of 5c

In a glovebox, a benzene-d_6 solution (300 μL) of Xyl-NC (10.4 mg, 79.2 μmol) was added to a benzene-d_6 solution (300 μL) of **1** (29.8 mg, 79.2 μmol) and 4-trifluoromethyl pyridine (46.2 μL, 399 μmol) in a 15 mL vial at room temperature. After stirring the reaction mixture for 10 min at room temperature, a benzene-d_6 solution (300 μL) of 1,3,5-trimethoxybenzene (13.1 mg, 78.4 μmol) was added to the crude product and the resulting mixture was stirred for 5 min at room temperature. An aliquot (600 μL) of the resulting solution was pipetted to a screw-capped NMR tube. After bringing the NMR tube out from the glovebox, ^{1}H NMR spectrum was recorded to estimate the NMR yield of **5c** (72%).

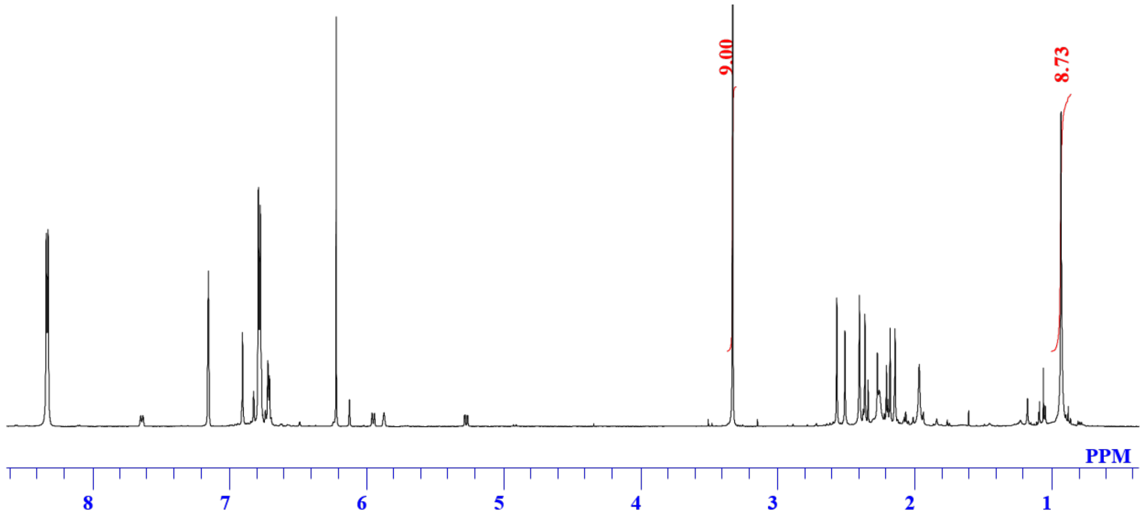

Figure S23. The ^{1}H NMR spectrum of the crude product for the synthesis of **5c**

Synthesis of 5d

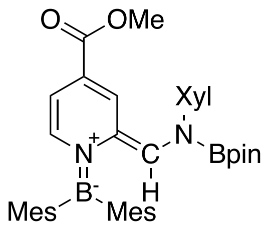

In a glovebox, a toluene solution (3 mL) of Xyl-NC (105 mg, 0.798 mmol) was added to a toluene solution (3 mL) of **1** (300 mg, 0.798 mmol) and 4-(methoxycarbonyl)pyridine (472 µL, 3.99 mmol) in a 30 mL vial at room temperature. During the reaction mixture was stirred at room temperature for 5 min, the color of the resulting solution turned to be green. Volatiles were removed from the reaction mixture under reduced pressure. The residue was recrystallized from hexane (–35 °C) to afford yellow crystals of **5d** (164.6 mg, 0.255 mmol, 32%). ^{1}H NMR (400 MHz, C_6D_6) δ 0.97 (s, 12H, CH_3 of pin), 2.01 (s, 6H, *o*-CH_3 of Xyl or Mes), 2.14 (s, 3H, *p*-CH_3 of Mes), 2.19 (s, 3H, *p*-CH_3 of Mes), 2.28 (br s, 6H, *o*-CH_3 of Xyl or Mes), 2.43 (s, 6H, *o*-CH_3 of Xyl or Mes), 3.29 (s, 3H, OCH_3), 5.96 (dd, *J* = 8, 1 Hz, 1H, CH), 6.13 (s, 1H, CH of pyridine), 6.66-6.75 (m, 5H, CH of Mes and Xyl), 6.78 (d, *J* = 8 Hz, 1H, CH of pyridine), 6.98 (br s, 3H, CH of Mes and pyridine); ^{11}B NMR (160.5 MHz, C_6D_6) δ 48 (s), 24 (s); ^{13}C NMR (126 MHz, C_6D_6) δ 17.9 (CH_3), 21.2 (CH_3) 22.6 (CH_3), 23.1 (CH_3), 23.2 (CH_3), 24.4 (CH_3 of pin), 51.0 (OCH_3), 83.6 (4° of pin), 106.6 (CH), 122.0 (4°), 123.0 (4°), 127.5 (CH), 128.8 (CH), 128.9 (CH), 129.0 (CH) 129.3 (CH), 135.8 (CH), 136.1 (4°), 136.6 (4°), 137.6 (4°), 138.3 (4°), 140.4 (4°), 141.2 (4°), 165.3 (COO); mp 187.2-190.8 °C (decomp.); Anal. Calcd for $C_{40}H_{50}B_2N_2O_4$: C, 74.55; H, 7.81; N, 4.35; Found: C, 74.26; H, 7.57; N, 4.34.

Estimation of NMR yield for the formation of 5d

In a glovebox, a benzene-*d*$_6$ solution (300 µL) of Xyl-NC (10.5 mg, 79.8 µmol) was added to a benzene-*d*$_6$ solution (300 µL) of **1** (30.0 mg, 79.8 µmol) and 4-(methoxycarbonyl)pyridine (47.2 µL, 399 µmol) in a 15 mL vial at room temperature. After stirring the reaction mixture for 10 min at room temperature, a benzene-*d*$_6$ solution (300 µL) of 1,3,5-trimethoxybenzene (13.7 mg, 81.4 µmol) was added to the crude product and the resulting mixture was stirred for 5 min at room temperature. An aliquot (600 µL) of the resulting solution was pipetted to a screw-capped NMR tube. After bringing the NMR tube out from the glovebox, ^{1}H NMR spectrum was recorded to estimate the NMR yield of **5d** (49%).

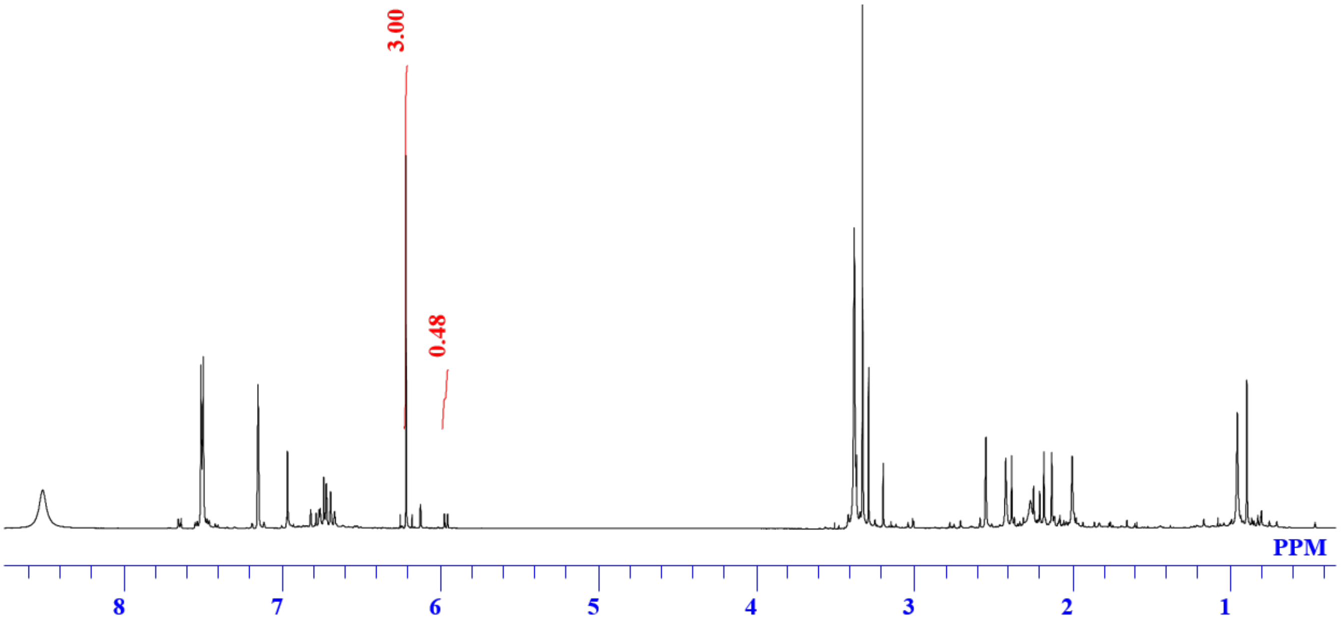

Figure S24. The ^{1}H NMR spectrum of the crude product for the synthesis of **5d**

Confirmation for the formation of 7a-c

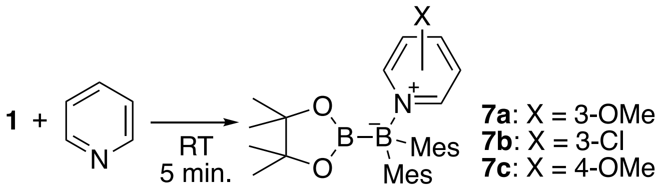

The reactions above were checked by ^{11}B NMR spectroscopy and two major signals in the following three experiments were tentatively assigned as **7a-c** without isolation of them.

Reaction of 1 with 3-methoxypyridine

In a glovebox, 3-methoxypyridine (42.5 µL, 421 µmol) was added to a toluene solution (558 µL) of **1** (15.0 mg, 39.9 µmol) in a 15 mL vial at room temperature. After stirring the reaction mixture for 10 min at room temperature, an aliquot (600 µL) of the resulting solution was pipetted to a screw-capped NMR tube. After bringing the NMR tube out from the glovebox, ^{11}B NMR spectra was recorded. In comparison with the ^{11}B NMR spectrum of **7**, we tentatively assigned this species as sp^2-sp^3 diborane **7a** by a coordination of pyridine derivative. ^{11}B NMR (160.5 MHz, C_6D_6) δ 39 (s), 1 (s).

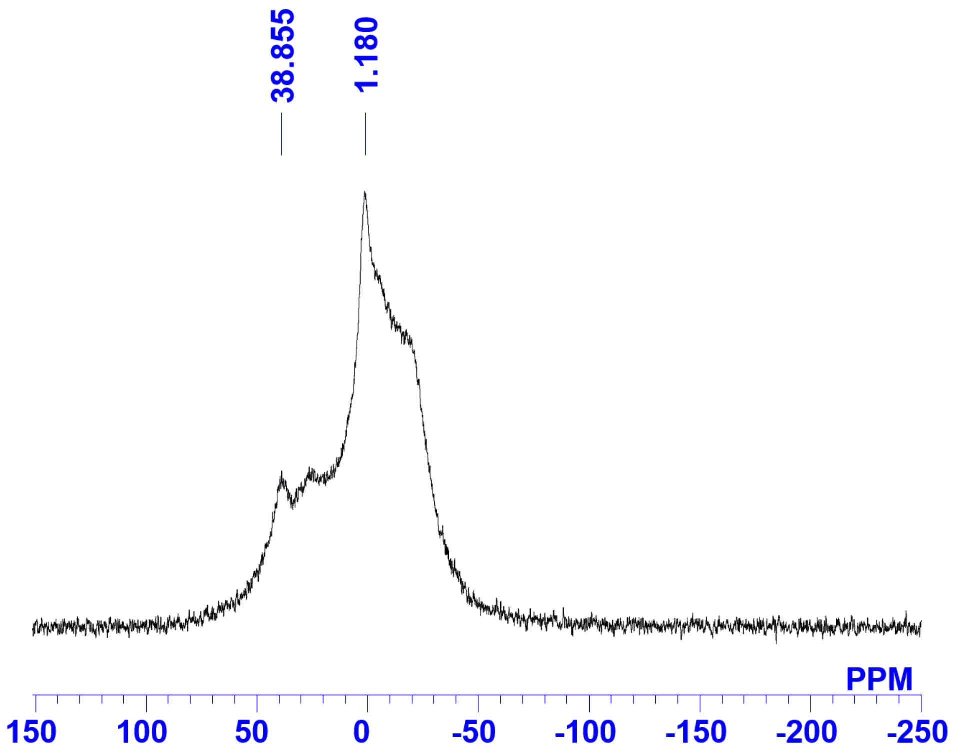

Figure S25. The ^{11}B NMR spectrum of the crude product in the reaction of **1** with 3-methoxypyridine

The reaction of 1 with 4-methoxypyridine

In a glovebox, 4-methoxypyridine (40.3 µL, 339 µmol) was added to a toluene solution (560 µL) of **1** (10.5 mg, 79.8 µmol) in a 15 mL vial at room temperature. After stirring the reaction mixture for 10 min at room temperature, an aliquot (600 µL) of the resulting solution was pipetted to a screw-capped NMR tube. After bringing the NMR tube out from the glovebox, ^{11}B NMR spectrum was recorded. In comparison with the ^{11}B

NMR spectrum of **7**, we tentatively assigned this species as sp^2-sp^3 diborane **7b** by a coordination of pyridine derivative. ^{11}B NMR (160.5 MHz, C_6D_6) δ 39 (s), –1 (s).

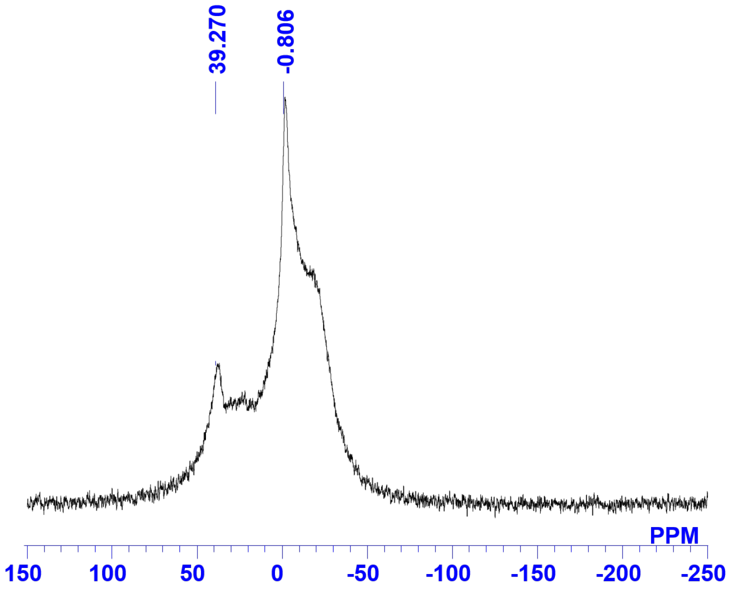

Figure S26. The ^{11}B NMR spectrum of the crude product in the reaction of **1** with 4-methoxypyridine

The reaction of 1 with 3-chloropyridine

In a glovebox, 3-chloropyridine (600 μL, 6.39 mmol) was added to a solid of **1** (15.0 mg, 39.9 μmol) in a 15 mL vial at room temperature. After stirring the reaction mixture for 10 min at room temperature, an aliquot (600 μL) of the resulting solution was pipetted to a screw-capped NMR tube. After bringing the NMR tube out from the glovebox, ^{11}B NMR spectrum was recorded. In comparison with the ^{11}B NMR spectrum of **7**, we tentatively assigned this species as sp^2-sp^3 diborane **7c** by a coordination of pyridine derivative. ^{11}B NMR (160.5 MHz, C_6D_6) δ 40 (s), 10 (s).

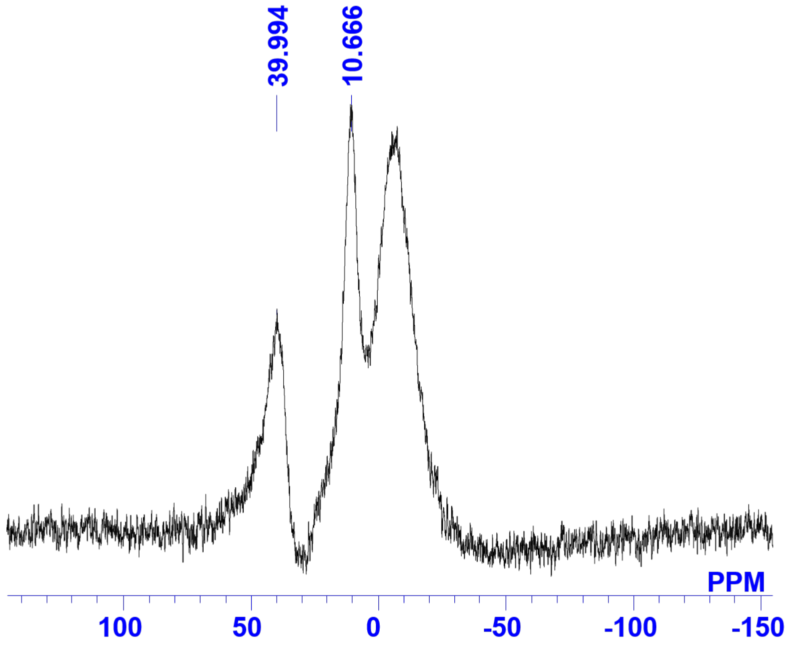

Figure S27. The ^{11}B NMR spectrum of the reaction of **1** with 3-chloropyridine

Synthesis of 9a

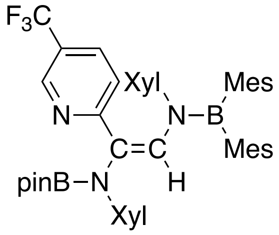

In a glovebox, a toluene solution (300 µL) of Xyl-NC (21.0 mg, 160 µmol) was added to a pyridine solution (254 µL) of **1** (30.0 mg, 79.8 µmol) in a 15 mL vial at room temperature. During the reaction mixture was stirred at room temperature for 5 min, the color of the resulting solution turned to be red. Volatiles were removed from the reaction mixture under reduced pressure. The residue was recrystallized from hexane (–35 °C) to afford yellow crystals of **9a** (8.0 mg, 10 µmol, 13%). Because of the existence of the equilibrium, signals in the ^{1}H, ^{11}B, and ^{13}C NMR spectrum could not be assigned as noted in the main text. Therefore, a characteristic signal of pyridine core at 8.42 ppm was used for the estimation of the NMR yield. ^{11}B NMR (160.5 MHz, C_6D_6, as an equilibrium mixture) δ 48 (br s), 22 (br s), 9 (br s, minor), 0.6 (br s, minor); mp 209.4-210.5 °C (decomp.); Anal. Calcd for $C_{48}H_{56}B_2F_3N_3O_2$: C, 73.39; H, 7.19; N, 5.35; Found: C, 73.11; H, 7.00; N, 5.40.

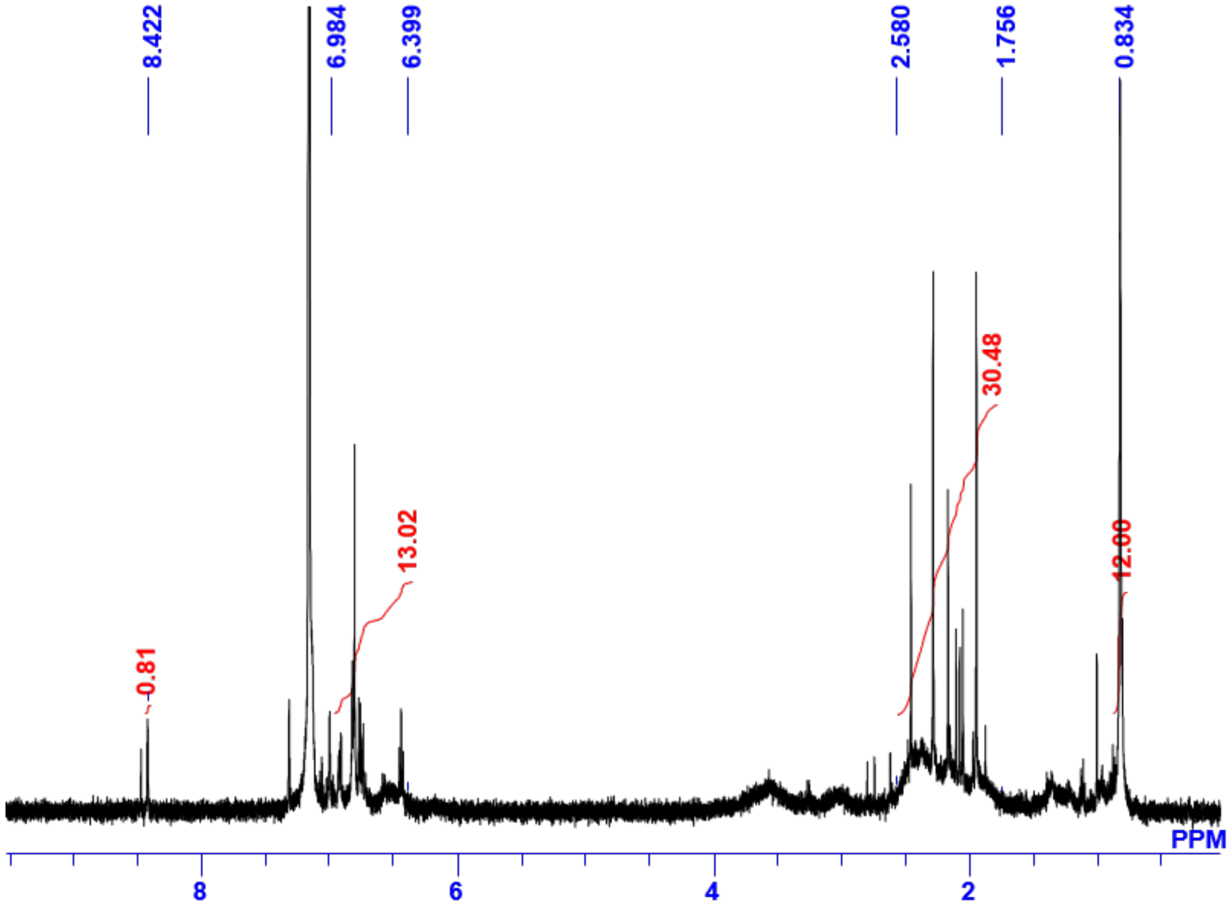

Figure S28. ^{1}H NMR spectrum of **9a** as an equilibrium mixture (only signals of major species are labeled)

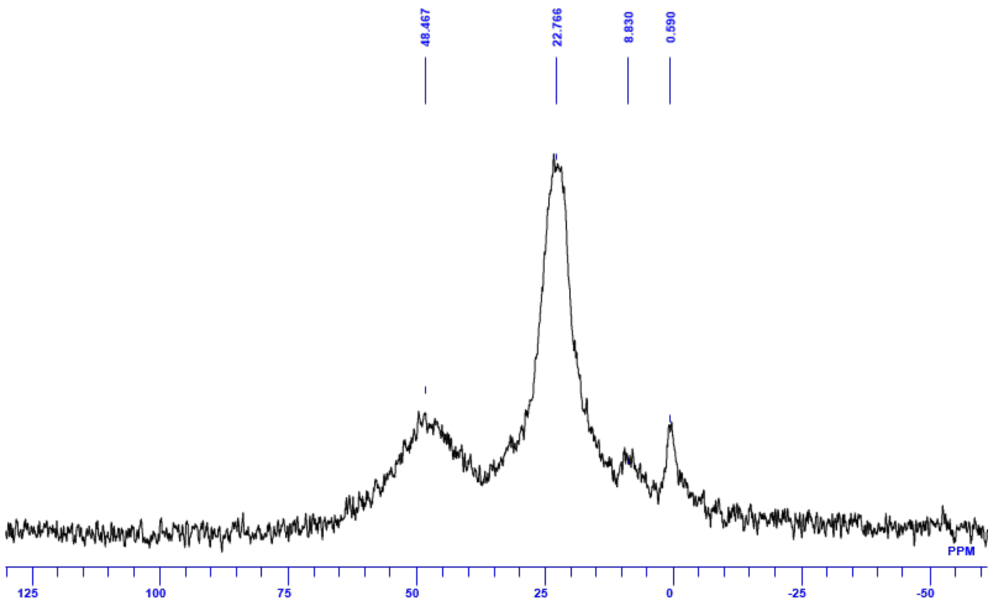

Figure S29. ^{11}B NMR spectrum of **9a** as an equilibrium mixture

Estimation of NMR yield for the formation of 9a

In a glovebox, a toluene solution (300 μL) of Xyl-NC (21.2 mg, 162 μmol) was added to a toluene solution (260 μL) of **1** (30.0 mg, 79.8 μmol) and 3-trifluoromethylpyridine (45.9 μL, 399 μmol) in a 15 mL vial at room temperature. After stirring the reaction mixture for 10 min at room temperature, volatiles were removed from the reaction mixture under reduced pressure. A benzene-d_6 solution (600 μL) of 1,3,5-trimethoxybenzene (13.4 mg, 79.8 μmol) was added to the residue and the resulting mixture was stirred for 5 min at room temperature. An aliquot (600 μL) of the resulting solution was pipetted to a screw-capped NMR tube. After bringing the NMR tube out from the glovebox, ^{1}H NMR spectrum was recorded to estimate the NMR yield of **9a** (31%).

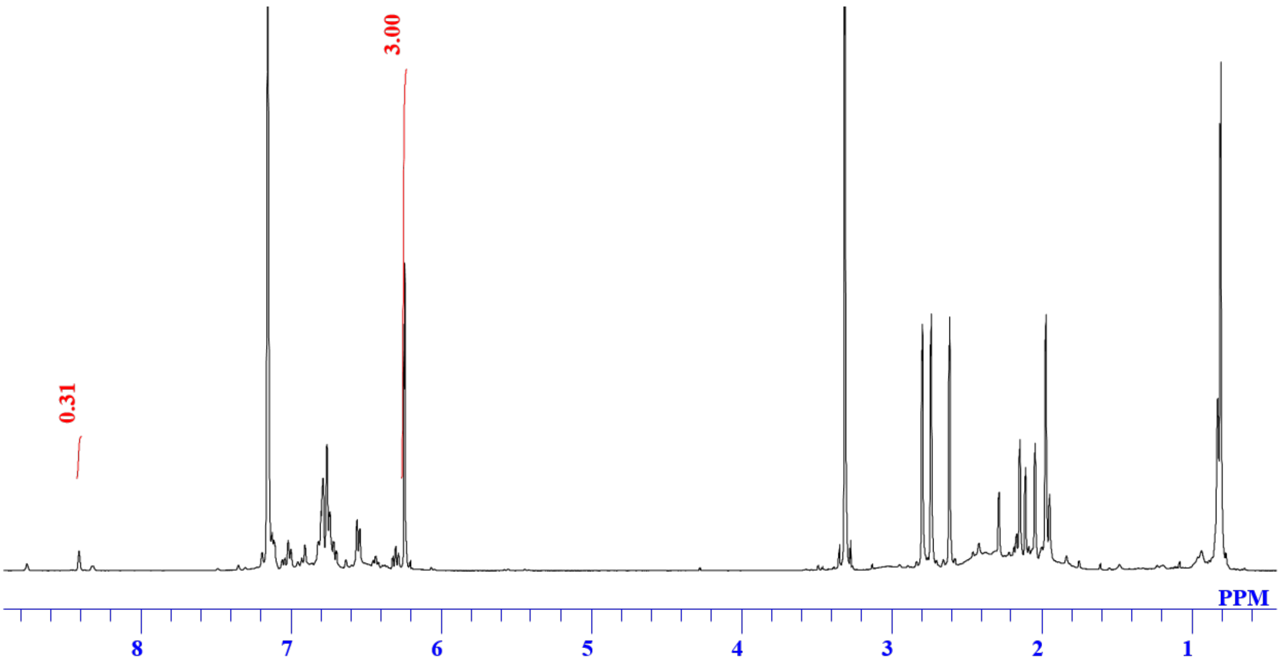

Figure S30. The ^{1}H NMR spectrum of the crude product for the synthesis of **9a**

Synthesis of 9b

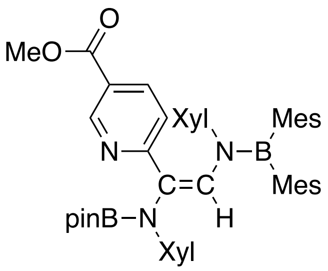

In a glovebox, a toluene solution (300 µL) of Xyl-NC (182 mg, 532 µmol) was added to a toluene solution (900 µL) of **1** (100 mg, 266 µmol) and 3-(methoxycarbonyl)pyridine (70.4 mg, 1.33 mmol) in a 30 mL vial at room temperature. During the reaction mixture was stirred at room temperature for 5 min, the color of the resulting solution turned to be red. Volatiles were removed from the reaction mixture under reduced pressure. The residue was recrystallized from hexane (–35 °C) to afford red crystals of **9b** (22.7 mg, 29.3 µmol, 11%). Because of the existence of the equilibrium, signals in the ^{1}H, ^{11}B, and ^{13}C NMR spectrum could not be assigned as noted in the main text. Therefore, a characteristic signal of pinacol moiety at 0.92 ppm was used for the estimation of the NMR yield. ^{11}B NMR (160.5 MHz, C_6D_6) δ 50 (br s), 22 (br s), 9 (br s, minor); mp 205.4-211.0 °C (decomp.); Anal. Calcd for $C_{49}H_{59}B_2N_3O_4$: C, 75.88; H, 7.67; N, 5.41; Found: C, 75.83; H, 7.87; N, 5.22.

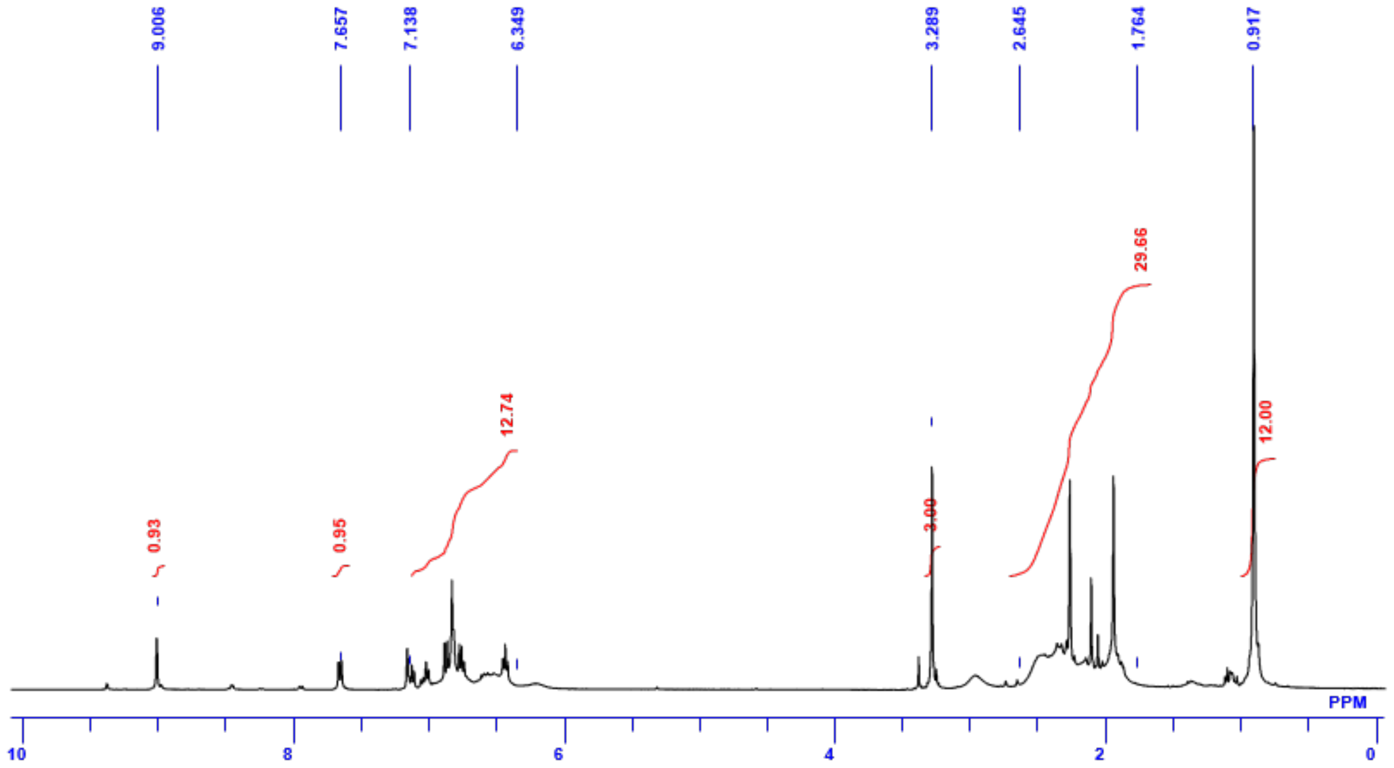

Figure S31. The ^{1}H NMR spectrum of **9b** as an equilibrium mixture (only signals of major species are labeled)

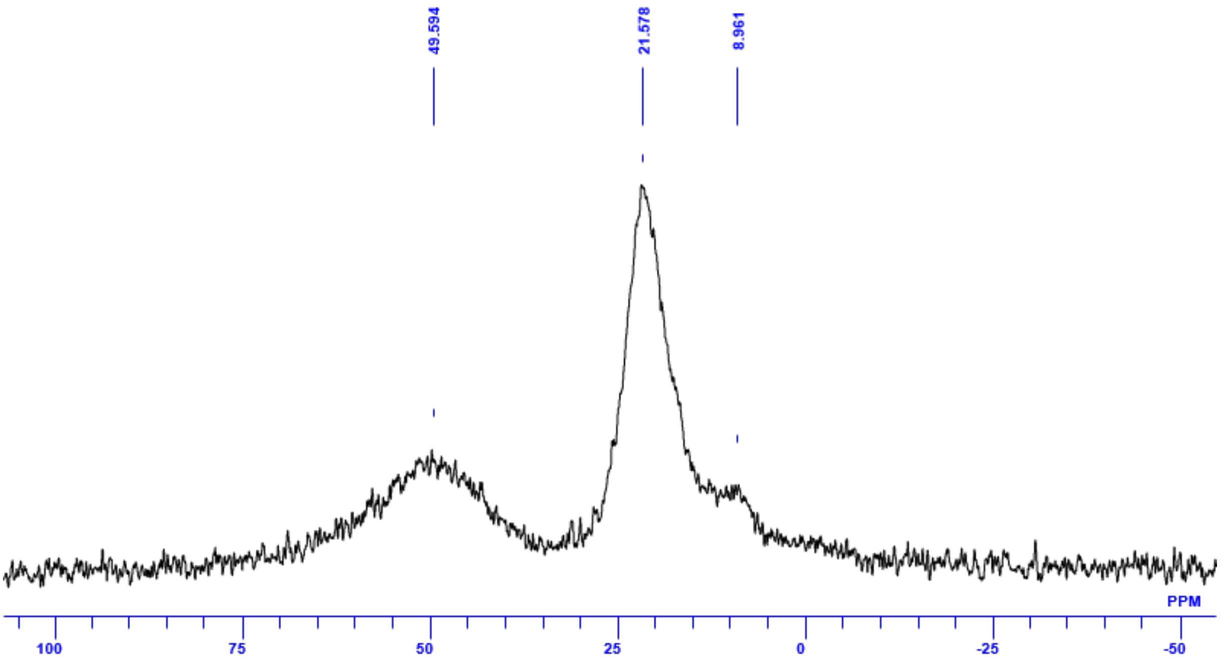

Figure S32. The ^{11}B NMR spectrum of **9b** as an equilibrium mixture

Estimation of NMR yield for the formation of 9b

In a glovebox, a toluene solution (300 µL) of Xyl-NC (21.2 mg, 162 µmol) was added to a toluene solution (300 µL) of **1** (30.0 mg, 79.8 µmol) and 3-(methoxycarbonyl)pyridine (54.6 mg, 399 µmol) in a 15 mL vial at room temperature. After stirring the reaction mixture for 10 min at room temperature, volatiles were removed from the reaction mixture under reduced pressure. A benzene-d_6 solution (600 µL) of 1,3,5-trimethoxybenzene (13.6 mg, 80.9 µmol) was added to the residue and the resulting mixture was stirred for 5 min at room temperature. An aliquot (600 µL) of the resulting solution was pipetted to a screw-capped NMR tube. After bringing the NMR tube out from the glovebox, ^{1}H NMR spectra was recorded to estimate the NMR yield of **9b** (24%).

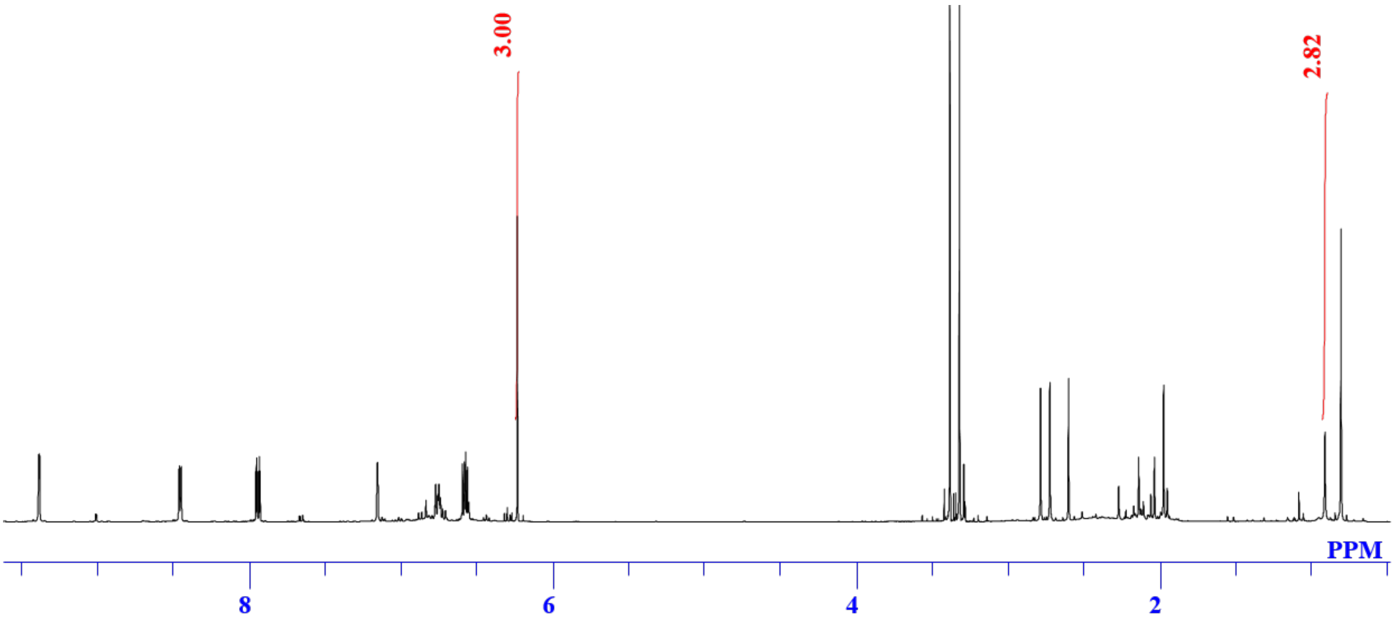

Figure S33. The ^{1}H NMR spectrum of the crude product for the synthesis of **9b**

Synthesis of 9c

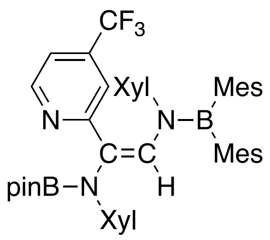

In a glovebox, a toluene solution (300 μL) of Xyl-NC (21.0 mg, 160 μmol) was added to a toluene solution (254 μL) of **1** (30.0 mg, 79.8 μmol) in a 30 mL vial at room temperature. During the reaction mixture was stirred at room temperature for 5 min, the color of the resulting solution turned to be red. Volatiles were removed from the reaction mixture under reduced pressure. The residue was recrystallized from hexane (–35 °C) to afford red crystals of **3c** (10.8 mg, 13.7 μmol, 17%). In this case, we could isolate **9c** and characterize all the signals in NMR spectra. A characteristic signal of pyridine core at 8.02 ppm was used for the estimation of the NMR yield (Figure SX). ^{1}H NMR (400 MHz, C_6D_6) δ 0.84 (s, 12H, CH_3 of pin), 1.78-2.72 (br, 24H, CH_3), 1.96 (s, 3H, CH_3), 2.27 (s, 3H, CH_3), 6.36-6.96 (br, 4H, CH of 2Mes), 6.42 (dd, J = 5, 1 Hz, 1H), 6.51 (t, J = 8 Hz, 2H), 6.73-6.84 (m, 4H), 8.02 (d, J = 6 Hz, 1H, 2-CH of pyridine); ^{11}B NMR (160.5 MHz, C_6D_6) δ 49 (s), 23 (s); ^{13}C NMR (126 MHz, C_6D_6) δ 19.3 (br, CH_3), 20.9 (CH_3), 21.2 (CH_3), 22.7 (CH_3), 23.7 (br, CH_3), 24.3 (CH_3 of pin), 82.4 (4° of pin), 116.9 (q, $^{3}J_{FC}$ = 4 Hz, CH), 120.3 (q, $^{3}J_{FC}$ = 4 Hz, CH), 123.6 (q, $^{1}J_{FC}$ = 273 Hz, CF_3), 125.0 (CH), 125.6 (CH), 126.5 (CH), 127.9 (4°), 128.2 (4°), 128.4 (br, CH), 128.7 (br, CH), 129.3 (CH), 131.0 (4°), 134.9 (CH), 136.0 (q, $^{2}J_{FC}$ = 32 Hz, 4°), 136.8 (br, 4°), 137.1 (4°), 137.4 (4°), 137.7 (br, 4°), 140.3 (br, 4°), 141.5 (4°), 141.7 (br, 4°), 144.6 (4°), 148.7 (CH), 157.2 (4°); mp 224.1-226.8 °C (decomp.); Anal. Calcd for $C_{48}H_{56}B_2F_3N_3O_2$: C, 73.39; H,7.19; N, 5.35; Found: C, 73.23; H, 6.91; N, 5.16.

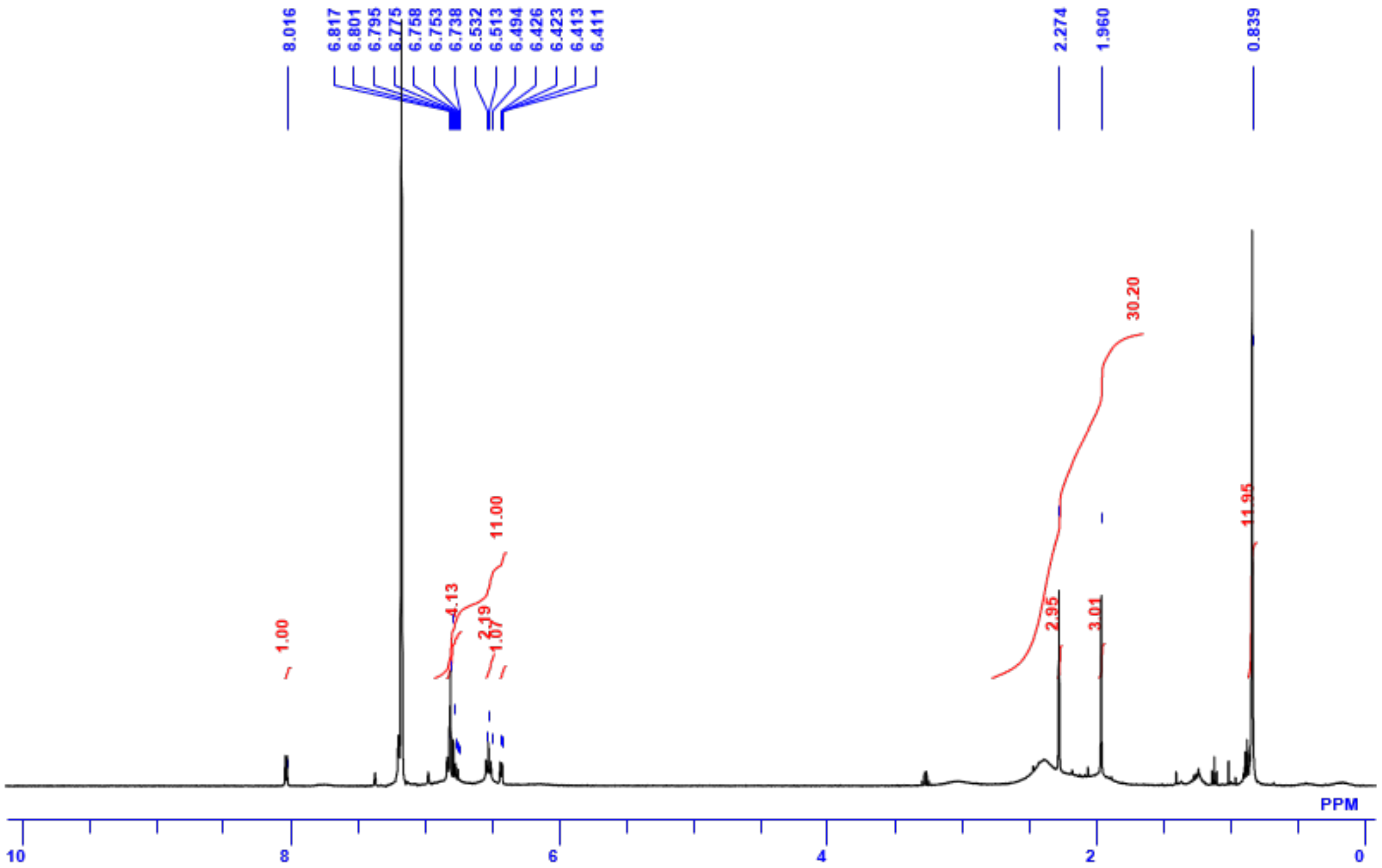

Figure S34. The ^{1}H NMR spectrum of the isolated **9c**

Estimation of NMR yield for the formation of 9c

In a glovebox, a toluene solution (300 μL) of Xyl-NC (21.0 mg, 160 μmol) was added to a toluene solution (260 μL) of **1** (30.0 mg, 79.8 μmol) and 4-trifluoromethylpyridine (46.2 μL, 399 μmol) in a 15 mL vial at room temperature. After stirring the reaction mixture for 10 min at room temperature, volatiles were removed from the reaction mixture under reduced pressure. A benzene-d_6 solution (600 μL) of 1,3,5-trimethoxybenzene (13.8 mg, 82.1 μmol) was added to the residue and the resulting mixture was stirred for 5 min at room temperature. An aliquot (600 μL) of the resulting solution was pipetted to a screw-capped NMR tube. After bringing the NMR tube out from the glovebox, ^{1}H NMR spectrum was recorded to estimate the NMR yield of **9c** (22%).

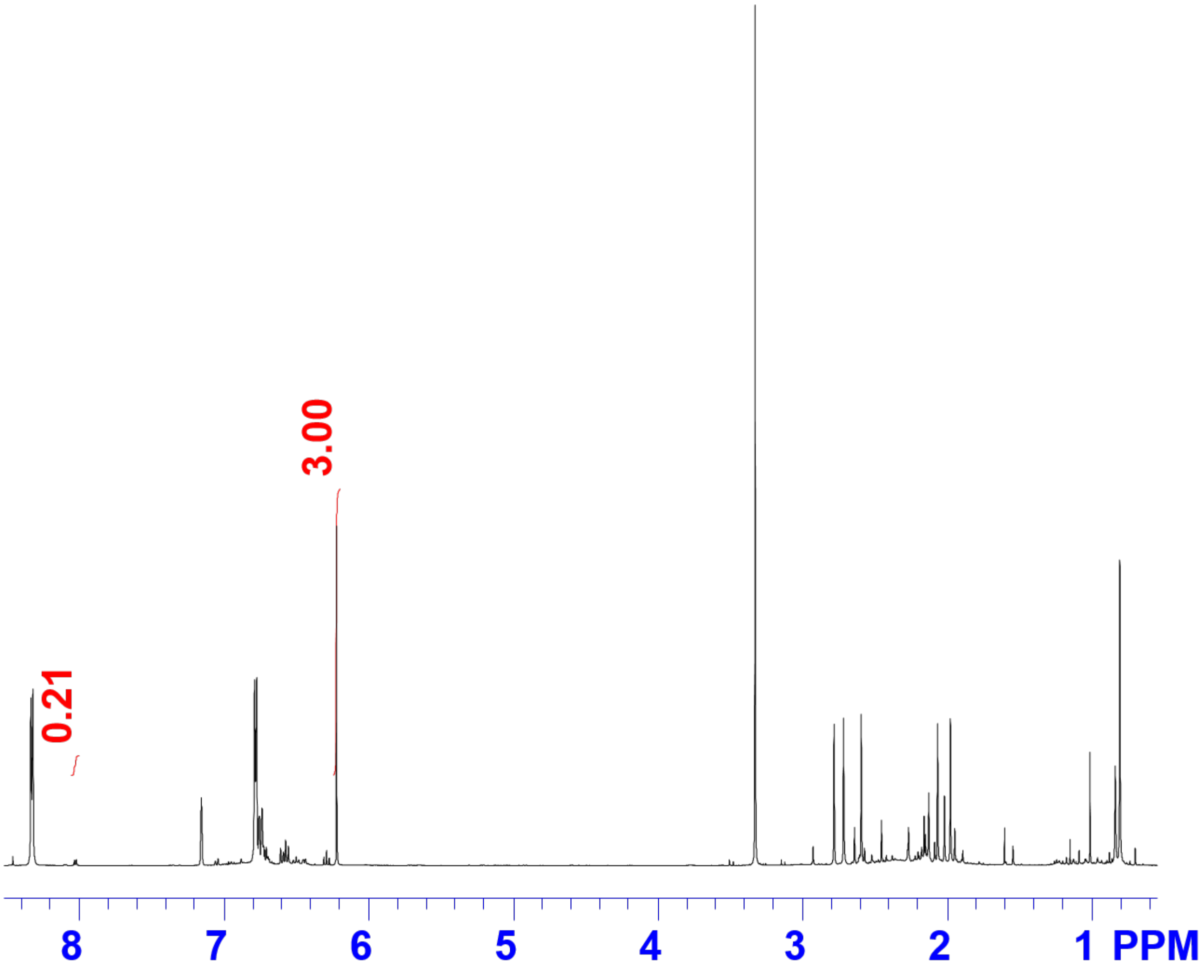

Figure S35. The ^{1}H NMR spectrum of the crude product for the synthesis of **9c**

Synthesis of 9d

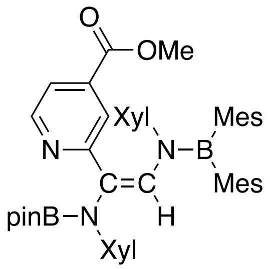

In a glovebox, a toluene solution (300 μL) of Xyl-NC (21.0 mg, 160 μmol) was added to a toluene solution (300 μL) of **1** (30.0 mg, 79.8 μmol) and 4-(methoxycarbonyl)yridine (47.2 μL, 399 μL) in a 15 mL vial at room temperature. During the reaction mixture was stirred at room temperature for 5 min, the color of the resulting solution turned to be red. Volatiles were removed from the reaction mixture under reduced pressure. The residue was recrystallized from hexane (–35 °C) to afford

red crystals of **9d** (10.8 mg, 13.7 μmol, 17%). Single crystals suitable for X-ray diffraction analysis were obtained by recrystallization from hexane at room temperature. In this case, we could isolate **9d** and characterize all the signals in NMR spectra. A characteristic signal of pyridine core at 8.13 ppm was used for the estimation of the NMR yield (Figure SX). ^{1}H NMR (400 MHz, C_6D_6) δ 0.92 (s, 12H, CH_3 of pin), 1.85-2.69 (br, 24H, CH_3), 1.96 (s, 3H, CH_3), 2.27 (s, 3H, CH_3), 3.34 (s, 3H, OCH_3), 6.12-6.96 (br, 5H, CH), 6.46 (t, J = 7 Hz, 1H, CH), 6.76 (t, J = 8 Hz, 1H, CH), 6.83 (d, J = 7 Hz, 2H), 6.87 (s, 1H), 7.07 (d, J = 5, 2 Hz, 1H, 3-CH of pyridine), 7.60 (s, 1H, 3-CH of pyridine), 8.13 (d, J = 5 Hz, 1H, 2-CH of pyridine); ^{11}B NMR (160.5 MHz, C_6D_6) δ 48 (br s), 22 (br s); ^{13}C NMR (126 MHz, C_6D_6, two B-bonded carbon atoms were not detected) δ 19.6 (br, CH_3), 20.9 (CH_3), 21.2 (CH_3), 23.7 (br, CH_3), 24.7 (CH_3 of pin), 51.8 (OCH_3), 82.1 (4° of pin), 120.6 (CH), 124.0 (CH), 125.2 (CH), 126.2 (CH), 128.1 (4°), 128.2 (4°), 128.5 (CH), 128.6 (CH), 128.7 (CH), 129.3 (4°), 130.9 (4°), 135.0 (4°), 135.9 (4°), 137.1 (4°), 137.3 (4°), 137.9 (4°), 140.6 (4°), 141.5 (4°), 144.5 (4°), 147.6 (CH), 155.9 (4°), 165.2 (C=O); mp 194.4-195.7 °C (decomp.); Anal. Calcd for $C_{49}H_{59}B_2N_3O_4$: C, 75.88; H, 7.67;.N, 5.42; Found: C, 75.97; H, 7.71; N, 5.17.

Estimation of NMR yield for the formation of 9d

In a glovebox, a benzene-d_6 solution (300 μL) of Xyl-NC (20.9 mg, 159 μmol) was added to a benzene-d_6 solution (300 μL) of **1** (29.9 mg, 79.5 μmol) and 4-(methoxycarbonyl)pyridine (47.2 μL, 399 μL) in a 15 mL vial at room temperature. After stirring the reaction mixture for 10 min at room temperature, a benzene-d_6 solution (600 μL) of 1,3,5-trimethoxybenzene (13.7 mg, 81.5 μmol) was added to the crude product and the resulting mixture was stirred for 5 min at room temperature. An aliquot (600 μL) of the resulting solution was pipetted to a screw-capped NMR tube. After bringing the NMR tube out from the glovebox, ^{1}H NMR spectrum was recorded to estimate the NMR yield of **9d** (24%).

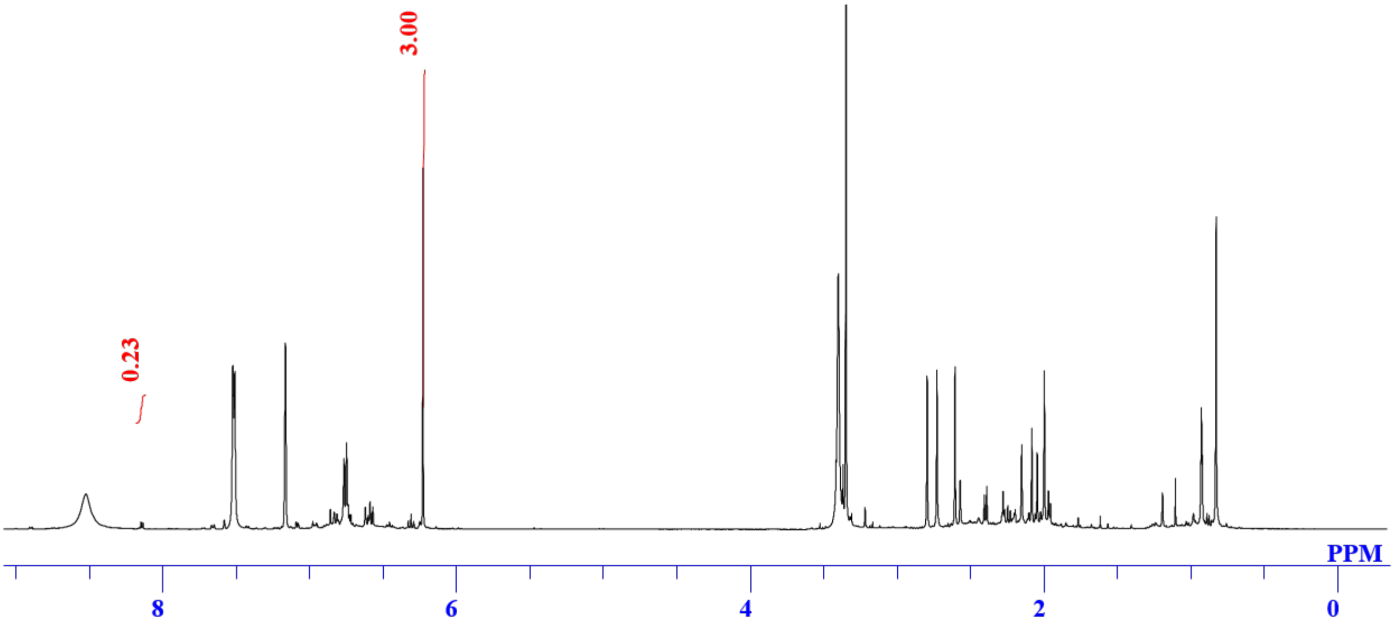

Figure S36. The ^{1}H NMR spectrum of the crude product for the synthesis of **9d**

Synthesis of 10

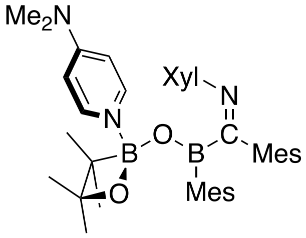

In a glovebox, a toluene solution (5 mL) of Xyl-NC (173 mg, 1.33 mmol) was added to a toluene solution (10 mL) of **1** (500 mg, 1.33 mmol) and 4-dimethylaminopyridine (163. mg, 1.33 mmol) in a 30 mL vial at room temperature. During the reaction mixture was stirred at room temperature for 5 min, the color of the resulting solution turned to be brown. Volatiles were removed from the reaction mixture under reduced pressure. The residue was recrystallized from toluene (–35 °C) to afford yellow crystals of **10** (174 mg, 0.276 mmol, 21%). ^{1}H NMR (400 MHz, C_6D_6) δ 1.21 (br s, 6H, CH_3 of oxaboretane), 1.49 (br s, 6H, CH_3 of oxaboretane), 1.76 (s, 3H, *p*-CH_3 of Mes), 1.85 (br s, 6H, *o*-CH_3 of Xyl or Mes), 2.08 (s, 3H, *p*-CH_3 of Mes), 2.12-2.17 (br m, 12H, $N(CH_3)_2$ and *o*-CH_3 of Xyl or Mes), 2.32 (br s, 6H, *o*-CH_3 of Mes), 6.08 (br, 2H, 3,5-CH of DMAP), 6.25 (s, 2H, CH of Mes), 6.64 (s, 2H, CH of Mes), 6.76 (br m, 2H, 3,5-CH of Xyl), 6.86 (t, J = 7 Hz, 1H, 4-CH of Xyl), 8.47 (br, 2H, 2,6-CH of DMAP); ^{11}B NMR (160.5 MHz, C_6D_6) δ 42 (s), 7 (s); ^{13}C NMR (126 MHz, C_6D_6) δ 20.8 (CH_3), 21.1 (CH_3), 21.2 (CH_3), 21.3 (CH_3), 21.5 (CH_3), 22.4 (CH_3), 27.5 (CH_3), 38.4 (NCH_3), 78.7 (4° of pin), 79.3 (4° of pin), 106.2 (br, CH), 121.9 (CH), 128.9 (CH), 129.1 (CH), 129.3 (CH), 135.6 (4°), 135.8 (4°), 136.0 (4°), 136.1 (4°), 138.8 (4°), 140.4 (4°), 140.9 (br, 4°), 141.5 (4°), 143.3 (br, 4°), 152.7 (4°), 154.7 (br, 4°), 192.4 (4°, C=N); mp 79.9-81.6 °C (decomp.); HRMS (ESI^{+}) Calc for $C_{40}H_{53}B_2N_3O_2$: 629.4324, found: 629.4321.

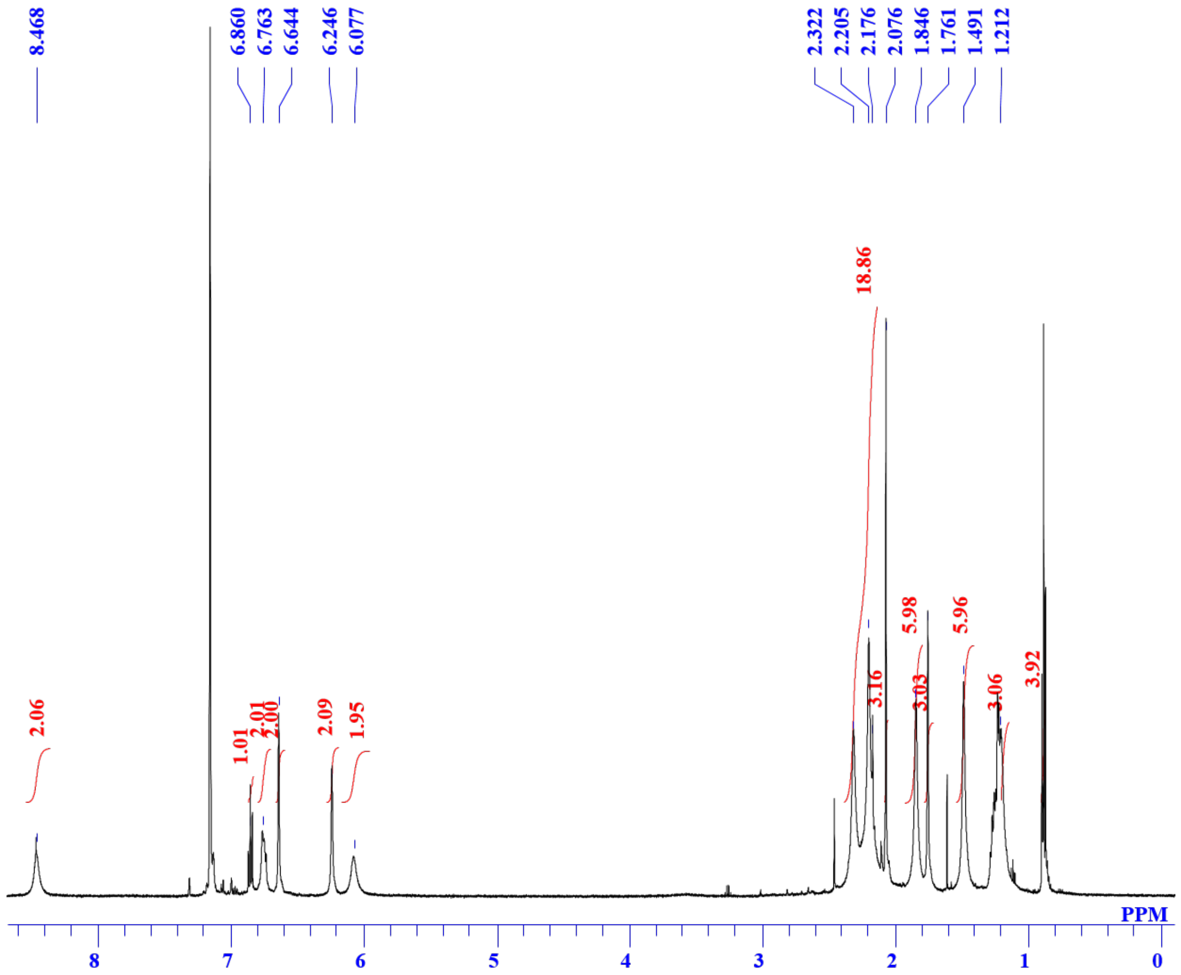

Figure S37. The ^{1}H NMR spectrum (benzene-*d*$_6$) of **10**

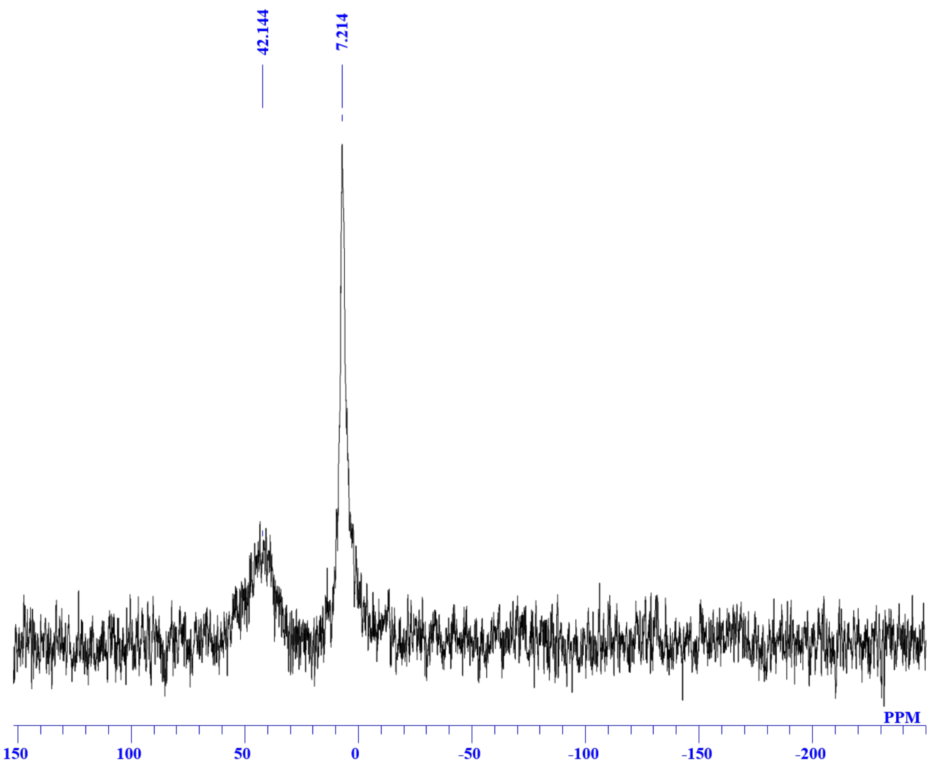

Figure S38. The ^{11}B NMR spectrum (benzene-d_6) of **10**

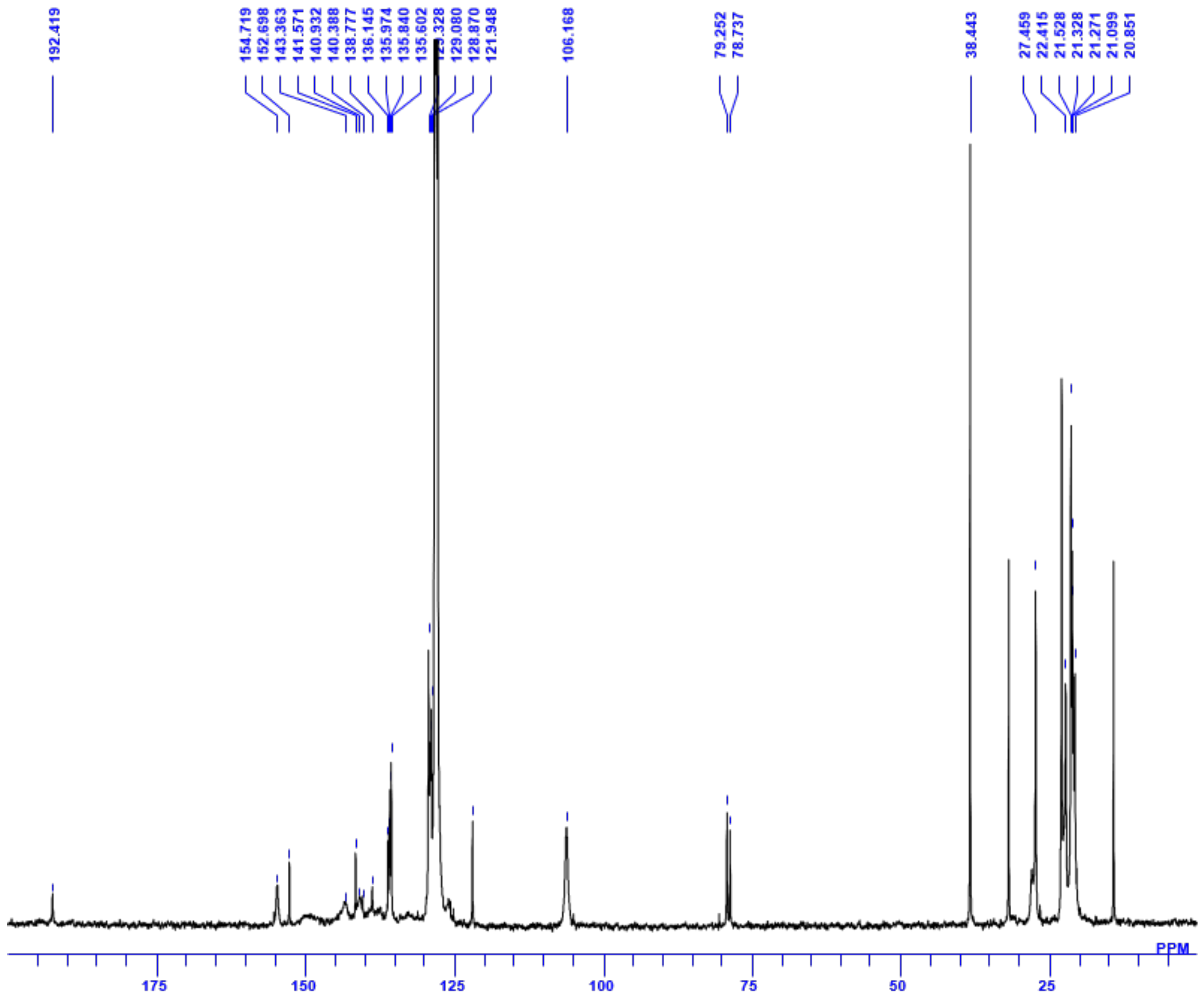

Figure S39. The ^{13}C NMR spectrum (benzene-d_6) of **10**

Estimation of NMR yield for the formation of 10

In a glovebox, a toluene solution (300 µL) of Xyl-NC (10.4 mg, 79.3 µmol) was added to a toluene solution (600 µL) of **1** (30.1 mg, 80.0 µmol) and 4-dimethylaminopyridine (9.9 mg, 81.0 µmol) in a 15 mL vial at room temperature. After stirring the reaction mixture for 10 min at room temperature, volatiles were removed from the reaction mixture under reduced pressure. A benzene-d_6 solution (600 µL) of 1,3,5-trimethoxybenzene (13.7 mg, 81.5 µmol) was added to residue and the resulting mixture was stirred for 5 min at room temperature. An aliquot (600 µL) of the resulting solution was pipetted to a screw-capped NMR tube. After bringing the NMR tube out from the glovebox, ^{1}H NMR spectrum was recorded to estimate the NMR yield of **10** (67%).

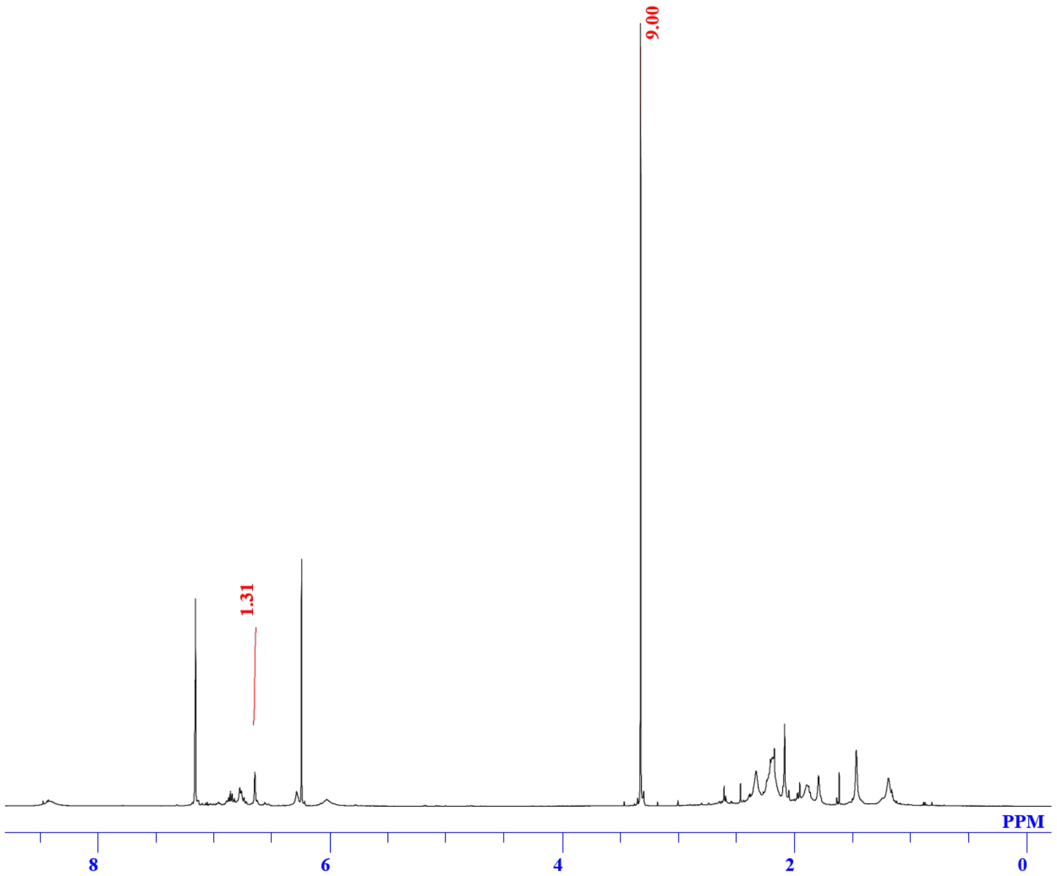

Figure S40. The ^{1}H NMR spectrum of the crude product for the synthesis of **10**

Synthesis of 11

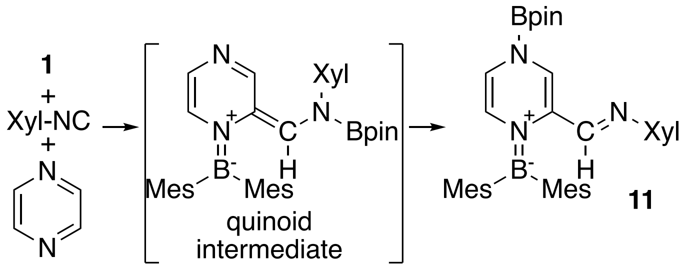

In a glovebox, a toluene solution (1.5 mL) of Xyl-NC (69.8 mg, 532 µmol) was added to a toluene solution (3 mL) of **1** (200 mg, 523 µmol) in a 15 mL vial at room temperature. During the reaction mixture was stirred at room temperature for 5 min, the color of the resulting solution turned to be green. Volatiles were removed from the reaction mixture under reduced pressure. The residue was recrystallized from hexane (–35 °C) to afford yellow crystals of **11** (72.8 mg, 124 µmol, 23%). Single crystals suitable for X-ray diffraction analysis were obtained by recrystallization from hexane at room temperature. ^{1}H NMR (400 MHz, C_6D_6) δ

0.94 (s, 12H, CH_3 of pin), 1.82 (s, 6H, *o*-CH_3 of Xyl or Mes), 2.11 (s, 3H, *p*-CH_3 of Mes), 2.16 (s, 3H, *p*-CH_3 of Mes), 2.22-2.66 (br, 12H, *o*-CH_3 of Xyl or Mes), 5.66 (dd, *J* = 6, 1 Hz, 1H, CH), 5.45 (d, *J* = 6 Hz, 1H, CH), 6.59 (br s, 2H, CH of Mes), 6.77, (br s, 2H, CH of Mes), 6.80 (s, 1H, CH), 6.83 (t, *J* = 8 Hz, 1H, 4-CH of Xyl), 6.93 (d, *J* = 8 Hz, 2H, 3,5-CH of Xyl), 7.01 (s, 1H, CH); ^{11}B NMR (160.5 MHz, C_6D_6) δ 45 (s), 22 (s); ^{13}C NMR (126 MHz, C_6D_6) δ 18.0 (CH_3), 21.16 (CH_3), 21.24 (CH_3), 22.3 (CH_3), 24.5 (CH_3), 84.3 (4° of pin), 115.6 (CH), 119.1 (CH), 123.4 (CH), 128.3 (CH), 128.7 (CH), 132.0 (CH), 137.5 (4°), 138.2 (4°), 139.7 (4°), 141.0 (4°), 141.5 (4°), 151.4 (4°), 157.3 (CH); mp 197.6-204.4 °C (decomp.); Anal. Calcd for $C_{37}H_{47}B_2N_3O_2$: C, 75.65; H, 8.07; N, 7.15; Found: C, 75.29; H, 8.17; N, 6.88.

Estimation of NMR yield for the formation of 11

In a glovebox, a toluene solution (300 μL) of Xyl-NC (10.5 mg, 79.8 μmol) was added to a toluene solution (300 μL) of **1** (30.0 mg, 79.8 μmol) and pyrazine (32.5 mg, 399 μmol) in a 15 mL vial at room temperature. After stirring the reaction mixture for 10 min at room temperature, volatiles were removed from the reaction mixture under reduced pressure. A benzene-*d*$_6$ solution (600 μL) of 1,3,5-trimethoxybenzene (13.6 mg, 80.9 μmol) was added to the residue and the resulting mixture was stirred for 5 min at room temperature. An aliquot (600 μL) of the resulting solution was pipetted to a screw-capped NMR tube. After bringing the NMR tube out from the glovebox, ^1H NMR spectrum was recorded to estimate the NMR yield of **11** (35%).

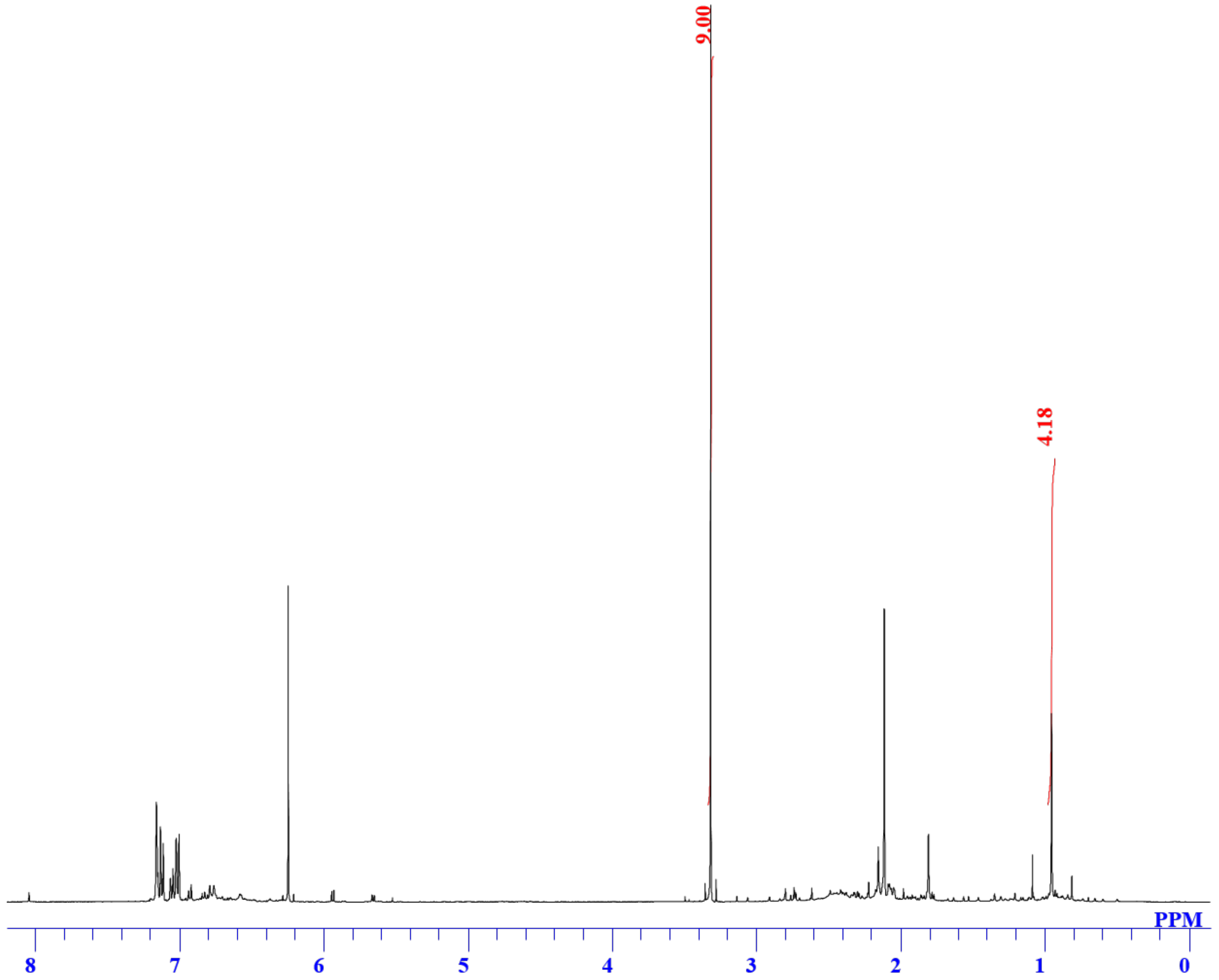

Figure S41. The ^1H NMR spectrum of the crude product for the synthesis of **11**

Synthesis of 12

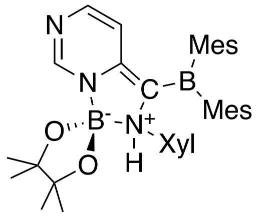

In a glovebox, a toluene solution (3 mL) of Xyl-NC (105 mg, 798 μmol) was added to a toluene solution (3 mL) of **1** (300 mg, 798 μmol) and pyrimidine (320 μL, 3.99 mmol) in a 30 mL vial at room temperature. During the reaction mixture was stirred at room temperature for 5 min, the color of the resulting solution turned to be green. Volatiles were removed from the reaction mixture under reduced pressure. The residue was recrystallized from hexane (–35 °C) to afford green crystals of **12** (209 mg, 359 μmol, 45%). ^{1}H NMR (400 MHz, C_6D_6) δ 0.63 (s, 3H, CH_3), 0.99 (s, 3H, CH_3), 1.08 (s, 3H, CH_3), 1.13 (s, 3H, CH_3), 1.51 (s, 3H, *p*-CH_3 of Mes), 1.56 (s, 3H, *p*-CH_3 of Mes), 2.12 (s, 3H, *o*-CH_3 of Mes or Xyl), 2.16 (s, 6H, *o*-CH_3 of Mes or Xyl), 2.44 (s, 3H, *o*-CH_3 of Mes or Xyl), 2.50 (s, 3H, *o*-CH_3 of Mes or Xyl), 2.76 (s, 3H, *o*-CH_3 of Mes or Xyl), 6.29 (dd, *J* = 7, 1 Hz, 1H, CH), 6.51 (d, *J* = 7 Hz, 1H, CH), 6.55 (br s, 1H, CH of Mes), 6.66 (br s, 1H, CH of Mes), 6.69 (br s, 1H, CH of Mes), 6.75 (t, *J* = 7 Hz, 1H, 4-CH of Xyl), 6.79 (d, *J* = 7 Hz, 2H, 3,5-CH of Xyl), 6.85 (br s, 1H, NH), 7.18 (s, 1H, CH of Mes), 8.50 (d, *J* = 1 Hz 1H, CH); ^{11}B NMR (160.5 MHz, C_6D_6) δ 56 (s), 10 (s); ^{13}C NMR (126 MHz, C_6D_6, three B-bonded carbon atoms were not detected) δ 16.6 (CH_3), 20.1 (br, CH_3), 20.5 (CH_3), 21.1 (CH_3), 22.2 (CH_3), 22.5 (CH_3), 22.8 (CH_3), 25.6 (CH_3), 25.9 (CH_3), 26.0 (CH_3), 27.7 (CH_3), 81.58 (4° of pin), 81.64 (4° of pin), 110.0 (CH), 126.4 (CH), 128.5 (CH), 128.6 (CH), 128.8 (CH), 129.8 (CH), 131.4 (CH), 133.8 (4°) 133.8 (4°), 137.4 (4°), 137.6 (4°), 139.4 (4°), 140.5 (4°), 140.6 (4°), 141.0 (4°), 142.0 (4°), 150.9 (CH), 152.1 (CH), 158.6 (CH); mp 223.2-223.5 °C (decomp.); Anal. Calcd for $C_{37}H_{47}B_2N_3O_2$: C, 75.65; H, 8.07; N, 7.15; Found: C, 75.63; H, 8.04; N, 6.78.

Estimation of NMR yield for the formation of 12

In a glovebox, a toluene solution (200 μL) of Xyl-NC (10.5 mg, 79.8 μmol) was added to a toluene solution (368 μL) of **1** (30.0 mg, 79.8 μmol) and pyrimidine (32.0 μL, 399 μmol) in a 15 mL vial at room temperature. After stirring the reaction mixture for 10 min at room temperature, volatiles were removed from the reaction mixture under reduced pressure. A benzene-d_6 solution (600 μL) of 1,3,5-trimethoxybenzene (13.3 mg, 79.1 μmol) was added to the residue and the resulting mixture was stirred for 5 min at room temperature. An aliquot (600 μL) of the resulting solution was pipetted to a screw-capped NMR tube. After bringing the NMR tube out from the glovebox, ^{1}H NMR spectrum was recorded to estimate the NMR yield of **12** (66%).

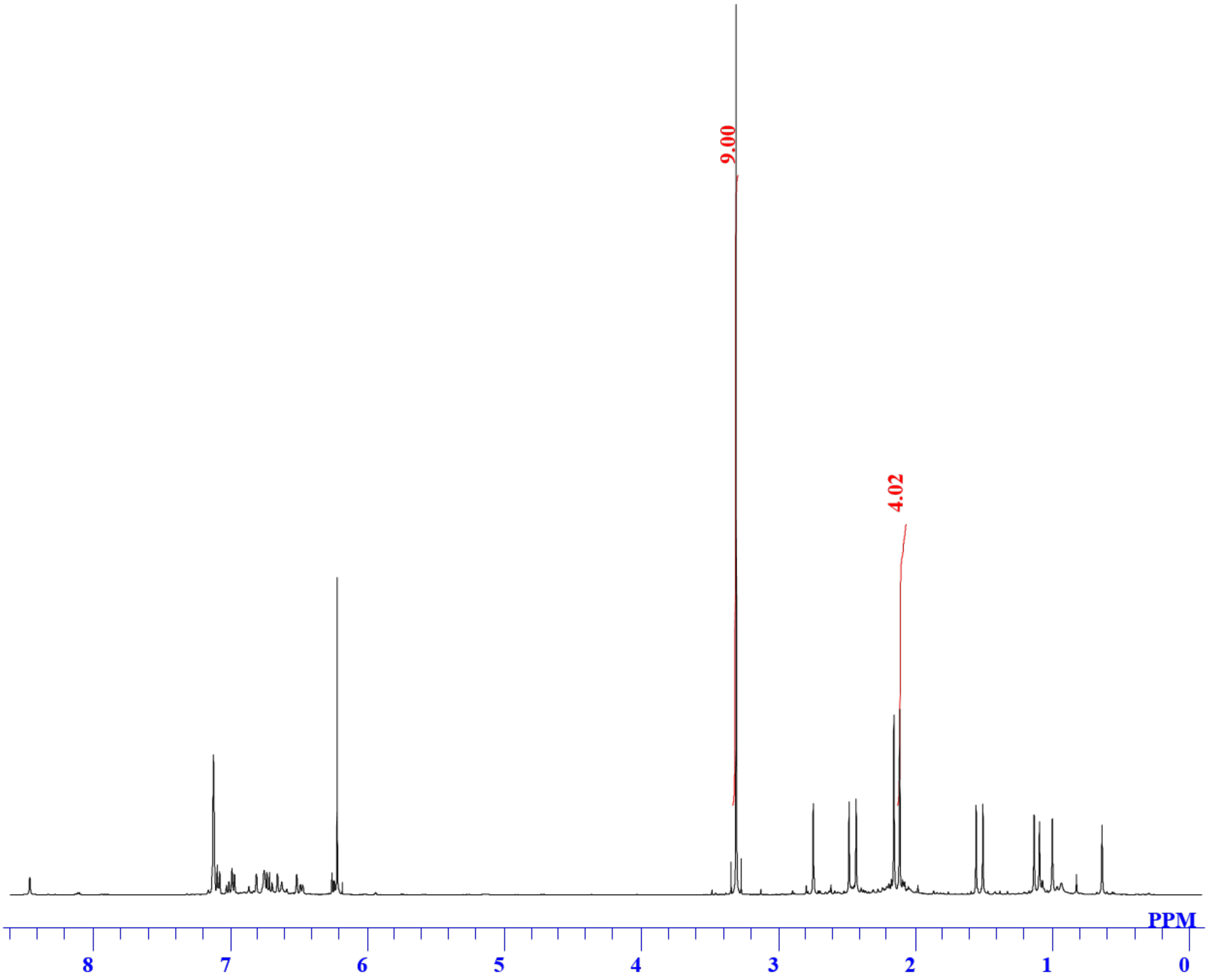

Figure S42. The ^{1}H NMR spectrum of the crude product for the synthesis of **12**

Synthesis of 13

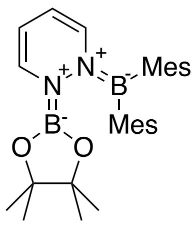

In a glovebox, a toluene solution (1.5 mL) of Xyl-NC (52.5 mg, 399 µmol) was added to a toluene solution (1.5 mL) of **1** (150 mg, 399 µmol) and pyridazine (145 µL, 2.00 mmol) in a 15 mL vial at room temperature. During the reaction mixture was stirred at room temperature for 5 min, the color of the resulting solution turned to be brown. Volatiles were removed from the reaction mixture under reduced pressure. The residue was recrystallized from hexane (–35 °C) to afford yellow crystals of **13** (74.5 mg, 128 µmol, 32%). ^{1}H NMR (400 MHz, C_6D_6) δ 0.53 (br s, 3H, CH_3 of pin), 0.83 (br s, 3H, CH_3 of pin), 0.91 (br s, 3H, CH_3 of pin), 0.96 (br s, 3H, CH_3 of pin), 2.17 (s, 6H, *o*-CH_3 of Mes), 2.20 (s, 3H, *p*-CH_3 of Mes), 2.26 (s, 3H, *p*-CH_3 of Mes), 2.79 (s, 3H, *o*-CH_3 of Mes), 2.90 (s, 3H, *o*-CH_3 of Mes), 4.95-5.04 (m, 2H, CH), 6.40 (d, J = 8 Hz, 1H, CH), 6.66 (m, 1H, CH), 6.67 (br s, 1H, CH of Mes), 6.70 (br s, 1H, CH of Mes), 6.87 (br s, 1H, CH of Mes), 6.91 (br s, 1H, CH of Mes); ^{11}B NMR (160.5 MHz, C_6D_6) δ 44 (s), 23 (s); ^{13}C NMR (126 MHz, C_6D_6) δ 21.2 (CH_3), 21.3 (CH_3), 22.1 (br, CH_3), 22.4 (CH_3), 23.0 (CH_3), 23.6 (br, CH_3), 24.6 (br, CH_3), 25.3 (br, CH_3), 83.6 (4°), 106.6 (br,

CH), 111.4 (br, CH), 127.9 (CH), 128.2 (CH), 128.6 (CH), 128.8 (CH), 129.0 (CH), 130.6 (CH), 133.4 (CH), 137.0 (4°), 137.37 (4°), 137.38 (4°), 140.29 (4°), 140.33 (4°), 141.3 (4°), 144.6 (4°); mp 54.5-57.8 °C (decomp.); Anal. Calcd for $C_{28}H_{38}B_2N_2O_2$: C, 75.65; H, 8.07; N, 7.15; Found: C, 75.29; H, 8.17; N, 6.88.

Estimation of NMR yield for the formation of 13

In a glovebox, a benzene-d_6 solution (300 µL) of Xyl-NC (10.5 mg, 79.8 µmol) was added to a toluene solution (368 µL) of **1** (30.0 mg, 79.8 µmol) and pyridazine (28.9 µL, 399 µmol) in a 15 mL vial at room temperature. After stirring the reaction mixture for 10 min at room temperature, a benzene-d_6 solution (600 µL) of 1,3,5-trimethoxybenzene (13.8 mg, 82.1 µmol) was added to the crude product and the resulting mixture was stirred for 5 min at room temperature. An aliquot (600 µL) of the resulting solution was pipetted to a screw-capped NMR tube. After bringing the NMR tube out from the glovebox, ^{1}H NMR spectrum was recorded to estimate the NMR yield of **13** (63%).

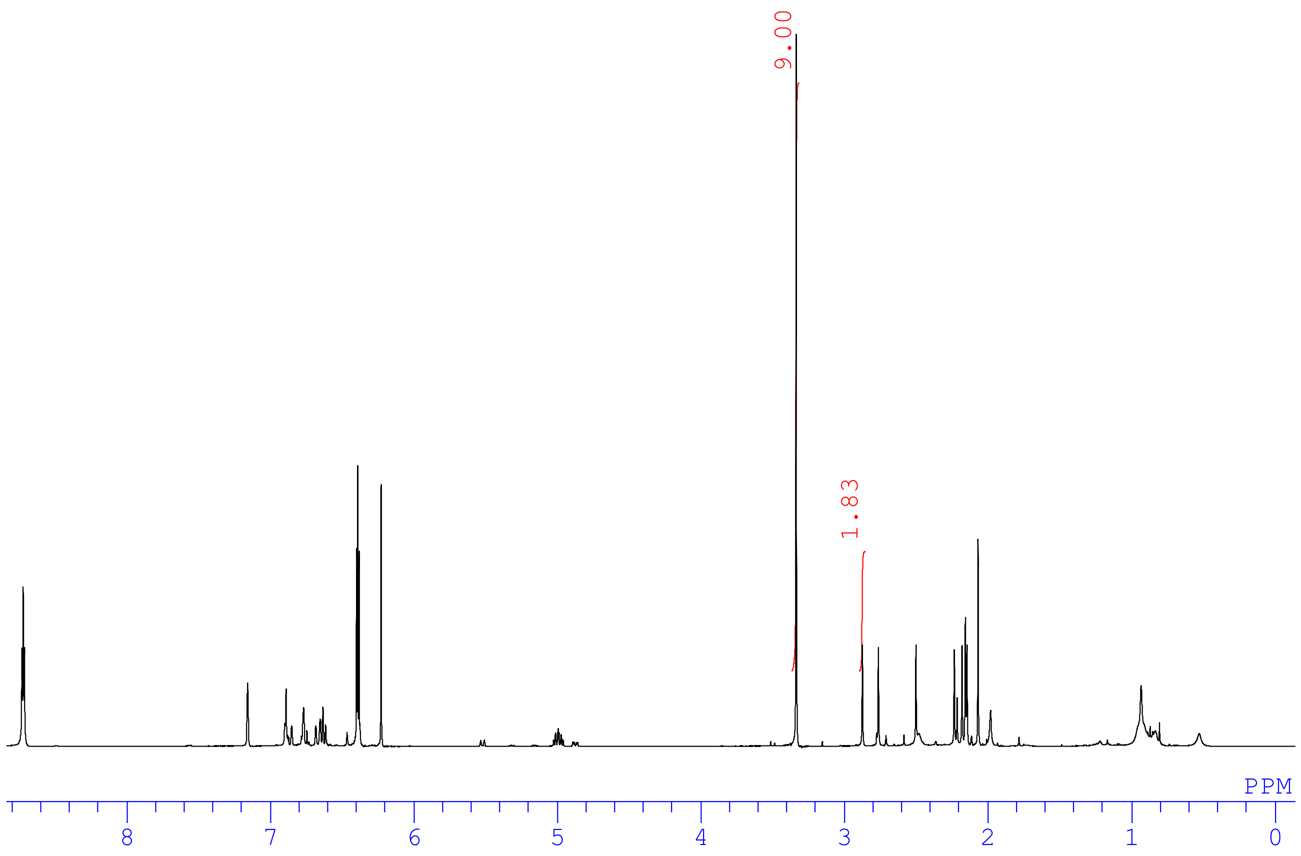

Figure S43. The ^{1}H NMR spectrum of the crude product for the synthesis of **13**

Synthesis of 5e

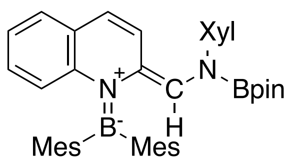

In a glovebox, a quinoline solution (5 mL) of Xyl-NC (175 mg, 1.33 mmol) was added to a quinoline solution (5 mL) of **1** (500 mg, 1.33 mmol) in a 120 mL Schlenk flask at room temperature. During the reaction mixture was stirred at room temperature for 5 min, the color of the resulting solution turned to be green. Volatiles were removed from the reaction mixture under reduced pressure at 90 °C. The residue was recrystallized from

hexane (–35 °C) to afford yellow crystals of **5e** (275 mg, 0.439 mmol, 33%). ^{1}H NMR (500 MHz, C_6D_6), δ 0.99 (br s, 12H, CH_3 of pin), 1.92 (s, 6H, *o*-CH_3 of Xyl or Mes), 2.14 (s, 3H, *p*-CH_3 of Mes), 2.22 (s, 3H, *p*-CH_3 of Mes), 2.42 (s, 6H, *o*-CH_3 of Xyl or Mes), 2.52 (s, 6H, *o*-CH_3 of Xyl or Mes), 5.55 (d, *J* = 10 Hz, 1H,CH), 5.90 (d, *J* = 10 Hz, 1H, CH), 6.63 (s, 1H, CH), 6.65-6.71 (m, 2H, CH), 6.73 (s, 2H, CH of Mes) , 6.76 (s, 2H, CH of Mes), 6.78 (m, 1H, CH) , 6.93 (s, 3H, CH of Xyl), 7.39 (m, 1H, CH); ^{11}B NMR (160.5 MHz, C_6D_6) δ 50 (s), 23 (s); ^{13}C NMR (126 MHz, C_6D_6) δ 17.7 (CH_3), 21.1 (CH_3), 21.2 (CH_3), 22.9 (CH_3), 24.0 (CH_3), 24.5 (CH_3), 83.4 (4°), 119.8 (CH), 122.6 (4°), 122.8 (CH), 123.5 (CH), 123.7 (CH), 126.7 (CH), 126.8 (CH), 127.4 (CH), 127.6 (CH), 128.36 (4°), 128.41 (CH), 128.6 (4°), 128.7 (CH), 128.8 (4°), 129.0, (CH), 129.3 (4°), 136.2 (4°), 136.8 (4°), 137.9 (4°), 139.1 (4°), 140.4 (4°), 141.2 (4°), 141.3 (br, 4°), 141.5 (br, 4°), 141.6 (4°), 141.9 (br, 4°), 143.9 (4°); mp 160.0-162.7 °C (decomp.); Anal. Calcd for $C_{42}H_{50}B_2N_2O_2$: C, 79.26; H, 7.92; N, 4.40; Found: C, 79.19; H, 7.79; N, 4.33.

Estimation of NMR yield for the formation of 5e

In a glovebox, a toluene solution (300 μL) of Xyl-NC (10.5 mg, 79.8 μmol) was added to a toluene solution (260 μL) of **1** (30.0 mg, 79.8 μmol) and quinoline (47.3 μL, 399 μmol) in a 15 mL vial at room temperature. After stirring the reaction mixture for 10 min at room temperature, volatiles were removed from the reaction mixture under reduced pressure. A benzene-*d*$_6$ solution (600 μL) of 1,3,5-trimethoxybenzene (13.2 mg, 78.5 μmol) was added to the crude product and the resulting mixture was stirred for 5 min at room temperature. An aliquot (600 μL) of the resulting solution was pipetted to a screw-capped NMR tube. After bringing the NMR tube out from the glovebox, ^{1}H NMR spectra was recorded to estimate the NMR yield of **5e** (54%).

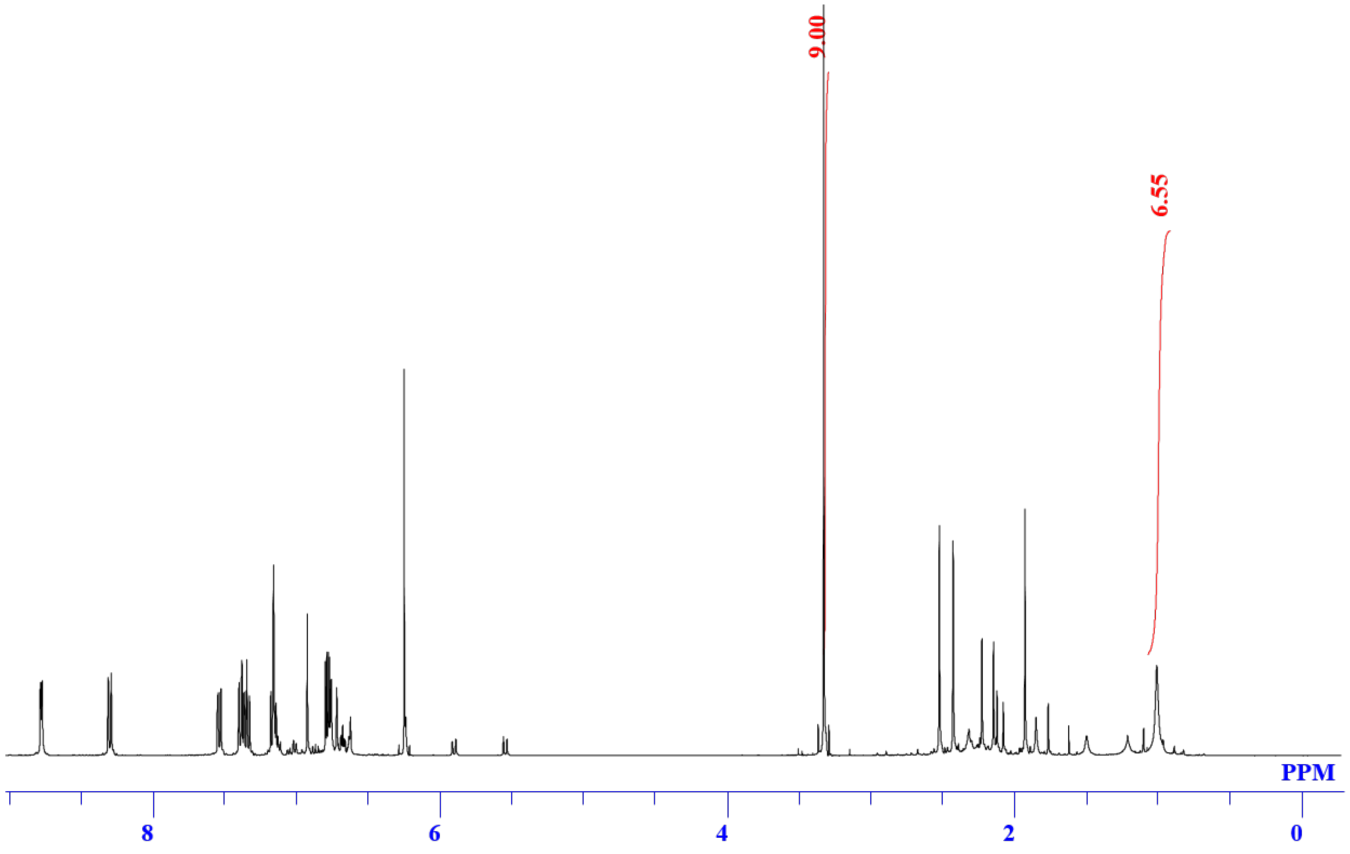

Figure S44. The ^{1}H NMR spectrum of the crude product for the synthesis of **5e**

Isolation of 9e by reaction of 6e with isocyanide

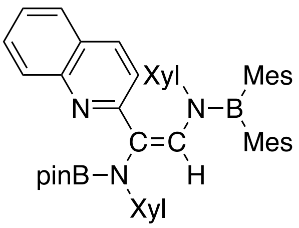

In a glovebox, a toluene solution (0.9 mL) of Xyl-NC (34.3 mg, 261 μmol) was added to a toluene solution (1.8 mL) of **5e** (166 mg, 261 μmol) in a 15 mL vial at room temperature. During the reaction mixture was stirred at 60 °C for 2 days, the color of the resulting solution turned to be red. Volatiles were removed from the reaction mixture under reduced pressure. The residue was recrystallized from acetonitrile (–35 °C) to afford orange crystals of **9e** (128 mg, 167 μmol, 64%). ^{1}H NMR (400 MHz, C_6D_6) δ 0.84 (s, 12H, CH_3 of pin), 1.65-2.80 (br, 24H, CH_3×8), 1.93 (CH_3), 2.32 (CH_3), 6.20 (t, J = 8 Hz, 1H, 4-CH of Xyl), 6.50 (br, 2H, CH of Mes), 6.67 (t, J = 8 Hz, 1H, 4-CH of Xyl), 6.67 (d, J = 8 Hz, 2H, 3,5-CH of Xyl), 6.91 (d, J = 8 Hz, 2H, 3,5-CH of Xyl), 6.98-7.06 (m, 2H, CH), 7.15 (d, J = 8 Hz, 2H, CH), 7.20 (d, J = 8 Hz, 2H, CH), 7.24 (dd, J = 7, 1 Hz, 2H, CH), 7.26 (dd, J = 7, 1 Hz, 2H, CH), 8.00 (d, J = 8 Hz, 1H, CH); ^{11}B NMR (160.5 MHz, C_6D_6) δ 49 (s), 24 (s); ^{13}C NMR (126 MHz, C_6D_6) δ 19.6 (br, CH_3), 20.9 (CH_3), 21.3 (CH_3), 23.0 (br, CH_3), 23.8 (CH_3), 24.2 (CH_3), 25.3 (br, CH_3), 82.4 (4° of pin), 122.6 (CH), 124.9 (CH), 125.8 (CH), 126.2 (CH), 127.04 (CH), 127.09 (CH), 127.9 (CH), 128.4 (CH), 128.6 (CH), 128.7 (CH), 129.4 (CH), 130.1 (CH), 132.4 (4°), 133.2 (CH), 135.0 (4°), 135.4 (4°), 137.0 (4°), 137.2 (4°), 137.9 (4°), 140.8 (4°), 141.7 (4°), 142.0 (4°), 144.7 (4°), 147.5 (4°), 156.1 (4°); mp 201.4-204.9 °C (decomp.); Anal. Calcd for $C_{53}H_{62}B_2N_4O_2$ (containing 1 eq. of acetonitrile): C, 78.71; H, 7.73; N, 6.93; Found: C, 78.42; H, 7.40; N, 6.62.

Estimation of NMR yield for the formation of 9e from 1

In a glovebox, a toluene solution (216 μL) of Xyl-NC (10.3 mg, 78.9 μmol) was added to a toluene solution (300 μL) of **6** (50.2 mg, 78.9 μmol) in a 15 mL vial at room temperature. After stirring the reaction mixture for 2 days at 60 °C, volatiles were removed from the reaction mixture under reduced pressure. A benzene-d_6 solution (600 μL) of 1,3,5-trimethoxybenzene (13.6 mg, 80.9 μmol) was added to the residue and the resulting mixture was stirred for 5 min at room temperature. An aliquot (600 μL) of the resulting solution was pipetted to a screw-capped NMR tube. After bringing the NMR tube out from the glovebox, ^{1}H NMR spectrum was recorded to estimate the NMR yield of **9e** (68%).

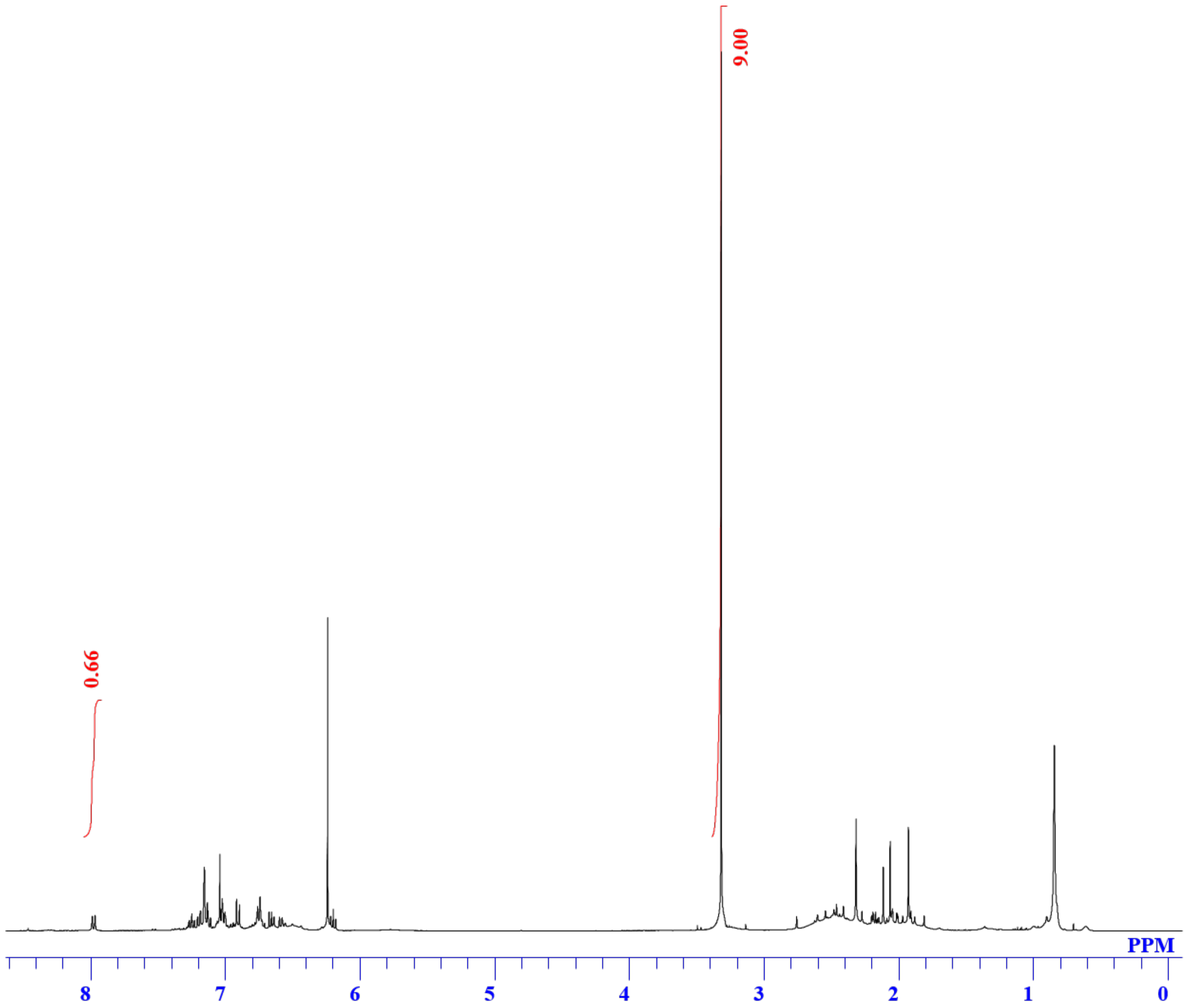

Figure S45. The ^{1}H NMR spectrum of the crude product for the synthesis of **9e**

Estimation of NMR yield for the reaction of 1 with 2 eq. Xyl-NC and quinoline

In a glovebox, a benzene-d_6 solution (300 μL) of Xyl-NC (21.0 mg, 160 μmol) was added to a benzene-d_6 solution (300 μL) of **1** (30.0 mg, 79.8 μmol) and quinoline (47.3 μL, 399 μmol) in a 15 mL vial at room temperature. After stirring the reaction mixture for 10 min at room temperature, a benzene-d_6 solution (600 μL) of 1,3,5-trimethoxybenzene (13.5 mg, 80.3 μmol) was added to the crude product and the resulting mixture was stirred for 5 min at room temperature. An aliquot (600 μL) of the resulting solution was pipetted to a screw-capped NMR tube. After bringing the NMR tube out from the glovebox, ^{1}H NMR spectrum was recorded to estimate the NMR yield of **3** (50%) and **5e** (21%). It should be noted that **9e** did not form at all in this reaction.

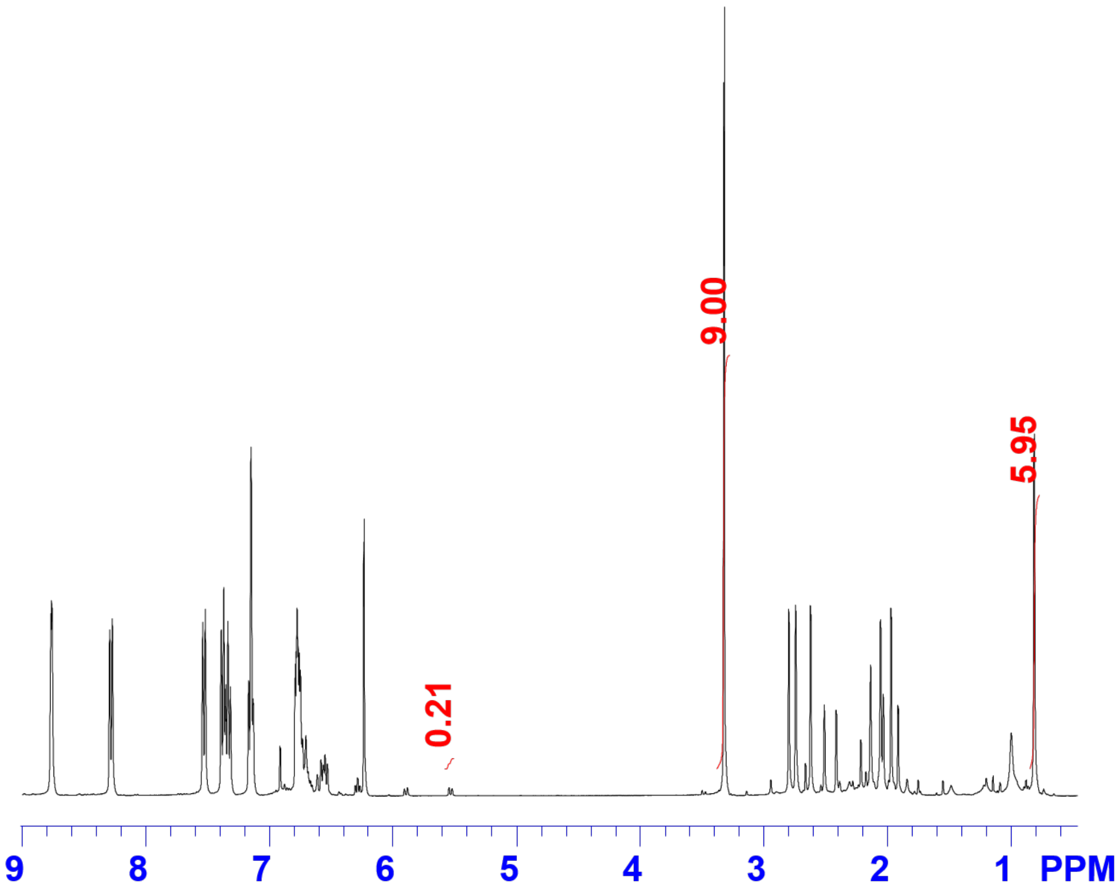

Figure S46. The ^{1}H NMR spectrum of the crude product for the reaction of **1** with 2 eq. Xyl-NC and quinoline

Synthesis of 4f

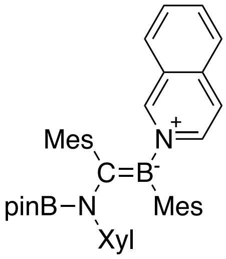

In a glovebox, a toluene solution (1.8 mL) of Xyl-NC (63.0 mg, 478 μmol) was added to a toluene solution (1.8 mL) of **1** (180 mg, 478 μmol) and isoquinoline (281 μL, 2.40 mmol) in a 15 mL vial at room temperature. During the reaction mixture was stirred at room temperature for 5 min, the color of the resulting solution turned to be black. Volatiles were removed from the reaction mixture under reduced pressure. The residue was recrystallized from hexane (–35 °C) to afford blue crystals of **4f** (74.3 mg, 117 μmol, 24%). ^{1}H NMR (400 MHz, C_6D_6) δ 1.09 (s, 12H, CH_3 of pin), 2.06 (s, 3H, CH_3), 2.33 (s, 9H, CH_3), 2.58 (s, 6H, CH_3), 2.88 (s, 6H, CH_3), 6.34 (d *J* = 7 Hz, 1H, 4-CH of isoquinoline), 6.69 (d, *J* = 8 Hz, 1H, 5-CH of quinoline), 6.70 (s, 2H, CH of Mes), 6.73 (d, *J* = 8 Hz, 1H, 6-CH of isoquinoline), 6.81 (dd, *J* = 8, 7 Hz, 1H, 7-CH of isoquinoline), 6.91 (t, *J* = 7 Hz, 1H, 4-CH of Xyl), 6.93 (d, *J* = 8 Hz, 1H, 8-CH of isoquinoline), 6.96 (d, *J* = 7 Hz, 2H, 3,5-CH of Xyl), 7.02 (s, 2H, CH of Mes), 7.89 (d, *J* = 7 Hz, 1H, 3-CH of isoquinoline), 8.81 (s, 1H, 1-CH of isoquinoline); ^{11}B NMR (160.5 MHz, C_6D_6) δ 29 (s), 24 (s); ^{13}C NMR (126 MHz, C_6D_6) δ 20.2 (CH_3), 21.1 (CH_3), 21.5 (CH_3), 22.5 (CH_3), 24.7 (CH_3), 25.3 (CH_3), 81.3 (4° of pin), 120.4 (CH), 121.0 (CH), 124.8 (CH), 125.9 (CH), 126.6 (CH), 127.0 (CH), 127.6 (CH), 127.9 (CH), 129.3 (CH), 129.5 (CH), 132.2 (CH), 134.1 (4°), 134.4 (4°), 135.9 (4°), 137.5 (CH), 138.2 (4°), 141.4 (4°), 142.7 (4°), 143.0 (4°), 145.3 (4°), 148.4 (CH), 153.0 (CH); mp 100.4-102.7 °C (decomp.); Anal. Calcd for $C_{42}H_{50}B_2N_2O_2$: C, 79.23; H, 7.92; N, 4.40; Found: C, 78.95; H, 7.65; N, 4.36.

Estimation of NMR yield for the formation of 4f

In a glovebox, a benzene-d_6 solution (300 μL) of Xyl-NC (10.5 mg, 79.8 μmol) was added to a toluene solution (368 μL) of **1** (30.0 mg, 79.8 μmol) and isoquinoline (46.9 μL, 399 μmol) in a 15 mL vial at room temperature. After stirring the reaction mixture for 10 min at room temperature, a benzene-d_6 solution (600 μL) of 1,3,5-trimethoxybenzene (13.4 mg, 79.8 μmol) was added to the crude product and the resulting mixture was stirred for 5 min at room temperature. An aliquot (600 μL) of the resulting solution was pipetted to a screw-capped NMR tube. After bringing the NMR tube out from the glovebox, ^{1}H NMR spectrum was recorded to estimate the NMR yield of **4f** (50%).

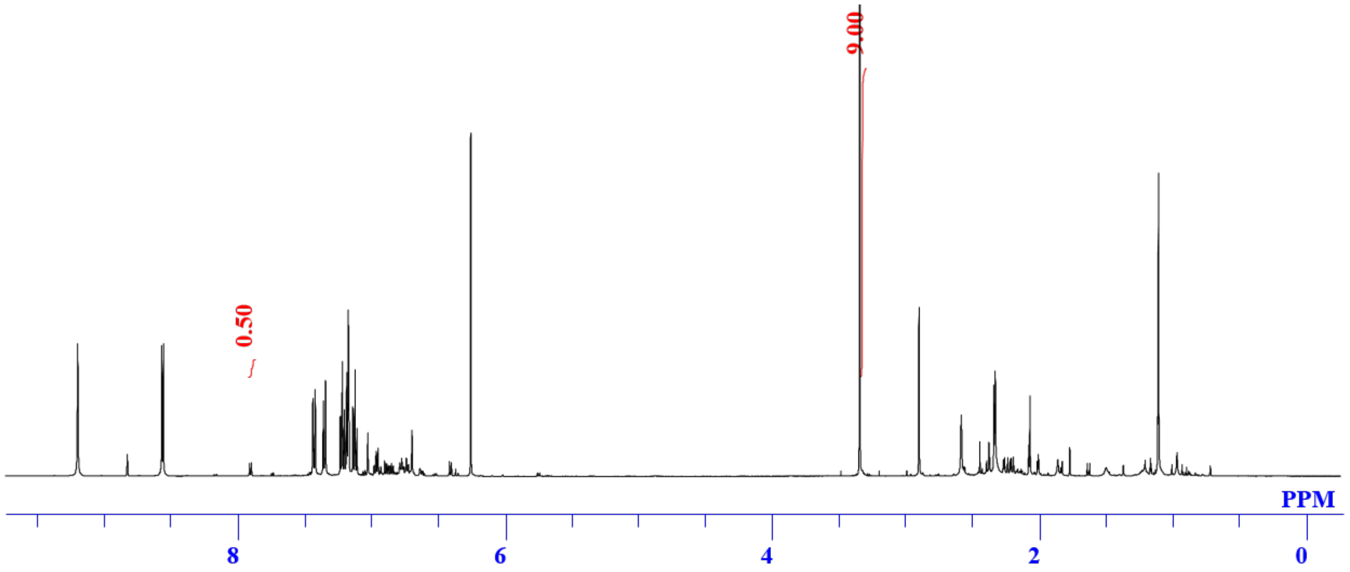

Figure S47. The ^{1}H NMR spectrum of the crude product for the synthesis of **4f**

Synthesis of 14 by hydrolysis of 5

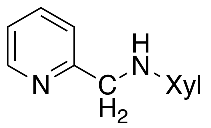

To a THF solution (5 mL) of **5** (150 mg, 0.256 mmol) in a 20 mL J.young tube, water (0.500 mL) was added at room temperature. The reaction mixture was stirred at 50 °C for 12 h (the color of the resulting solution turned to be red). The solvent was removed in vacuo to yield a brown oil, which was further subjected to flush chromatography on silica gel with a mixed eluent (AcOEt : hexane = 2 : 1). The first band (Rf = 0.86) was collected and solvent was removed by evaporation to afford **14** as brown oil (41.3 mg, 76 %). Spectroscopic data of 14 matched with those in the literature.[2]

Details for X-ray diffraction analysis

Details of the crystal data and a summary of the intensity data collection parameters are listed in Table S1. In each case a suitable crystal was mounted with mineral oil (Aldrich) or perfluoropolyalkylether (viscosity 80 cSt., abcr GmbH) to the glass fiber and transferred to the goniometer of Rigaku VariMax Saturn CCD diffractometer with graphite-monochromated MoKα radiation (λ = 0.71075 Å). All the following procedure for analysis, Yadokari-XG was used as a graphical interface.[3] The structures were solved by direct methods with (SIR-2014)[4] and refined by full-matrix least-squares techniques against F^2 (SHELXL-2014).[5] The intensities were corrected for Lorentz and polarization effects. The non-hydrogen atoms were refined anisotropically. Hydrogen atoms were placed using AFIX instructions. All the obtained crystal structures except those in the main text are shown in Figures S48-S58.

Table S1. Crystallographic data and structure refinement details for **4**, **4a**, **4b**, **5**, **5a**, **5b**, **5c**, **5d**, **5e**, **6**, **7**, **9a**, **9b**, **9c**, **9d**, **10**, **11**, **12**, and **13**

	**4**	**4a**	**4b**	**5**
formula	$C_{38}H_{48}B_2N_2O_2$	$C_{45}H_{64}B_2N_2O_3$	$C_{38}H_{47}B_2ClN_2O_2$	$C_{38}H_{48}B_2N_2O_2$
fw	586.40	702.60	620.84	586.40
T (K)	93(2)	93(2)	93(2)	93(2)
λ (Å)	0.71075	0.71075	0.71075	0.71075
cryst syst	Monoclinic	Orthorhombic	Monoclinic	Monoclinic
space group	$P2_1/a$	$Pna2_1$	$P2_1/c$	$P2_1/n$
a, (Å)	17.600(2)	19.208(3)	10.777(5)	8.565(3)
b, (Å)	19.466(2)	13.253(2)	8.468(4)	33.036(12)
c, (Å)	20.232(3)	16.091(3)	38.298(17)	11.991(5)
α, (°)	90	90	90	90
β, (°)	96.305(2)	90	95.088(7)	90.444(4)
γ, (°)	90	90	90	90
V, (Å^3)	6889.6(15)	4096.2(12)	3481(3)	3393(2)
Z	8	4	4	4
D_{calc}, (g / cm^3)	1.131	1.139	1.185	1.148
μ (mm^{-1})	0.068	0.069	0.145	0.069
F(000)	2528	1528	1328	1264
cryst size (mm)	0.23×0.19×0.09	0.23×0.13×0.08	0.20×0.18×0.01	0.21×0.06×0.03
2θ range, (deg)	3.039-26.000	3.074-27.490	3.064-25.999	2.995-25.998
reflns collected	50487	32588	29769	31248
indep reflns/R_{int}	13497/0.0566	9364/0.0717	6830/0.1037	6669/0.1301
Params	817	474	418	409
GOF on F^2	1.128	1.025	1.163	1.101
R_1, wR_2 [I>2σ(I)]	0.0749, 0.1645	0.0700, 0.1510	0.0983, 0.1784	0.0904, 0.1686
R_1, wR_2 (all data)	0.0994, 0.1804	0.0952, 0.1669	0.1323, 0.1962	0.1455, 0.1963

	**5a**	**5b**	**5c**	**5d**	**5e**
formula	$C_{117}H_{141}B_6F_9N_6O_6$	$C_{45}H_{62}B_2N_2O_4$	$C_{39}H_{47}B_2F_3N_2O_2$	$C_{40}H_{50}B_2N_2O_4$	$C_{89}H_{112}B_4N_4O_4$
fw	1963.21	716.58	654.40	644.44	1345.06
T (K)	93(2)	93(2)	93(2)	93(2)	93(2)
λ (Å)	0.71075	0.71075	0.71075	0.71075	0.71075
cryst syst	Orthorhombic	Triclinic	Monoclinic	Triclinic	Triclinic
space group	$P2_12_12_1$	P-1	$P2_1/c$	P-1	P-1
a, (Å)	16.004(4)	11.4491(18)	14.252(3)	8.4242(16)	9.894(3)
b, (Å)	21.403(5)	12.8149(17)	17.527(3)	15.698(3)	12.714(4)
c, (Å)	32.930(8)	15.929(2)	14.877(3)	28.081(5)	16.323(4)
α, (°)	90	74.652(8)	90	89.956(7)	101.384(5)
β, (°)	90	86.760(8)	93.855(3)	87.717(6)	91.511(4)
γ, (°)	90	65.452(5)	90	89.896(6)	100.757(4)
V, (Å^3)	11279(5)	2046.2(5)	3707.7(11)	3710.7(12)	1973.2(10)
Z	4	2	4	4	1
D_{calc}, (g / cm^3)	1.156	1.163	1.172	1.154	1.132
μ (mm^{-1})	0.080	0.072	0.081	0.073	0.067
F(000)	4176	776	1392	1384	726
cryst size (mm)	0.23×0.15×0.01	0.27×0.23×0.20	0.10×0.08×0.01	0.18×0.10×0.02	0.24×0.23×0.10
2θ range, (deg)	3.112-25.000	3.051-27.459	3.010-24.999	3.059-25.000	3.197-27.452
reflns collected	94264	16987	25208	25540	16334
indep reflns/R_{int}	19805/0.0668	9031/0.0445	6508/0.0759	12818/0.0842	8672/0.0526
Params	1511	535	482	993	490
GOF on F^2	1.143	0.968	1.101	1.061	0.992
R_1, wR_2 [I>2σ(I)]	0.0893, 0.2177	0.0750, 0.2066	0.0767, 0.1719	0.0962, 0.1939	0.0659, 0.1350
R_1, wR_2 (all data)	0.0961, 0.2235	0.1155, 0.2377	0.1162, 0.1975	0.1958, 0.2616	0.1217, 0.1656

	**6**	**7**	**9a**	**9b**	**9c**
formula	$C_{53}H_{63}B_2N_3O_2$	$C_{29}H_{39}B_2NO_2$	$C_{48}H_{56}B_2F_3N_3O_2$	$C_{49}H_{59}B_2N_3O_4$	$C_{48}H_{56}B_2F_3N_3O_2$
fw	795.68	455.23	785.57	775.61	785.57
T (K)	93(2)	93(2)	93(2)	93(2)	93(2)
λ (Å)	0.71075	0.71075	0.71075	0.71075	0.71075
cryst syst	Triclinic	Monoclinic	Monoclinic	Triclinic	Monoclinic
space group	P-1	$P2_1/n$	$P2_1/c$	P-1	$P2_1/n$
a, (Å)	11.821(3)	8.7230(16)	11.335(4)	8.034(3)	14.692(7)
b, (Å)	12.930(3)	23.773(4)	10.991(4)	10.856(4)	11.210(5)
c, (Å)	16.755(4)	13.264(3)	35.202(13)	26.173(10)	27.115(12)
α, (°)	101.050(3)	90	90	97.069(5)	90
β, (°)	95.8819(19)	99.499(3)	95.263(5)	97.132(8)	105.567(7)
γ, (°)	111.862(3)	90	90	102.212(9)	90
V, (Å^3)	2289.7(9)	2712.8(9)	4367(3)	2187.4(15)	4302(3)
Z	2	4	4	2	4
D_{calc}, (g / cm^3)	1.154	1.115	1.195	1.178	1.213
μ (mm^{-1})	0.069	0.067	0.080	0.073	0.082
F(000)	856	984	1672	832	1672
cryst size (mm)	0.15×0.10×0.08	0.20×0.20×0.03	0.20×0.20×0.05	0.20×0.20×0.04	0.23×0.15×0.05
2θ range, (deg)	3.052-26.000	3.006-25.000	3.026-24.997	3.020-24.998	3.002-24.999
reflns collected	16981	18336	35806	14963	35133
indep reflns/R_{int}	8800/0.0645	4741/0.0450	7671/0.0713	7572/0.0745	7551/0.0903
Params	555	317	594	538	594
GOF on F^2	0.989	1.129	1.151	1.060	1.198
R_1, wR_2 [I>2σ(I)]	0.0652, 0.1318	0.0652, 0.1570	0.0829, 0.1914	0.0897, 0.1617	0.0985, 0.2047
R_1, wR_2 (all data)	0.1247, 0.1605	0.0811, 0.1711	0.1014, 0.2045	0.1616, 0.2030	0.1242, 0.2199

	**9d**	**10**	**11**	**12**	**13**
formula	$C_{49}H_{59}B_2N_3O_4$	$C_{54}H_{69}B_2N_3O_2$	$C_{37}H_{47}B_2N_3O_2$	$C_{37}H_{47}B_2N_3O_2$	$C_{28}H_{38}B_2N_2O_2$
fw	775.61	813.74	587.39	587.39	456.22
T (K)	93(2)	93(2)	93(2)	93(2)	93(2)
λ (Å)	0.71075	0.71075	0.71075	0.71075	0.71075
cryst syst	Triclinic	Monoclinic	Monoclinic	Monoclinic	Monoclinic
space group	P-1	$P2_1/c$	$P2_1/a$	$P2_1/n$	$P2_1/n$
a, (Å)	10.951(2)	10.6242(17)	15.319(2)	10.981(2)	10.134(3)
b, (Å)	14.210(3)	20.635(4)	13.7912(18)	10.430(2)	18.980(6)
c, (Å)	16.118(3)	22.708(4)	17.481(3)	28.550(6)	13.580(4)
α, (°)	104.201(3)	90	90	90	90
β, (°)	92.6541(11)	100.578(3)	112.051(2)	94.578(4)	96.976(6)
γ, (°)	111.940(3)	90	90	90	90
V, (Å^3)	2228.6(8)	4893.6(14)	3422.9(8)	3259.7(11)	2592.7(14)
Z	2	4	4	4	4
D_{calc}, (g / cm^3)	1.156	1.104	1.140	1.197	1.169
μ (mm^{-1})	0.072	0.065	0.069	0.073	0.071
F(000)	832	1760	1264	1264	984
cryst size (mm)	0.20×0.10×0.02	0.21×0.20×0.16	0.24×0.12×0.08	0.15×0.08×0.02	0.15×0.07×0.03
2θ range, (deg)	3.005-27.477	3.031-24.999	3.023-27.460	3.123-27.457	3.022-24.999
reflns collected	18527	32992	27777	26271	17541
indep reflns/R_{int}	9851/0.0401	8602/0.0629	7810/0.0441	7333/0.0671	4511/0.0537
Params	538	605	409	409	317
GOF on F^2	0.986	1.115	1.032	1.065	1.105
R_1, wR_2 [I>2σ(I)]	0.0590, 0.1245	0.0985, 0.2623	0.0603, 0.1402	0.0762, 0.1771	0.0632, 0.1424
R_1, wR_2 (all data)	0.1020, 0.1501	0.1416, 0.3028	0.0847, 0.1588	0.1068, 0.1986	0.0829, 0.1560

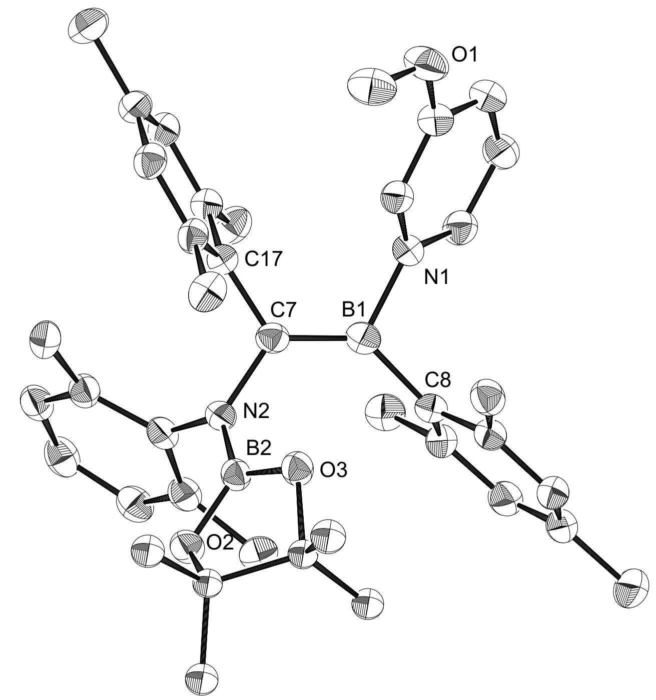

Figure S48. Molecular structure of **4a**

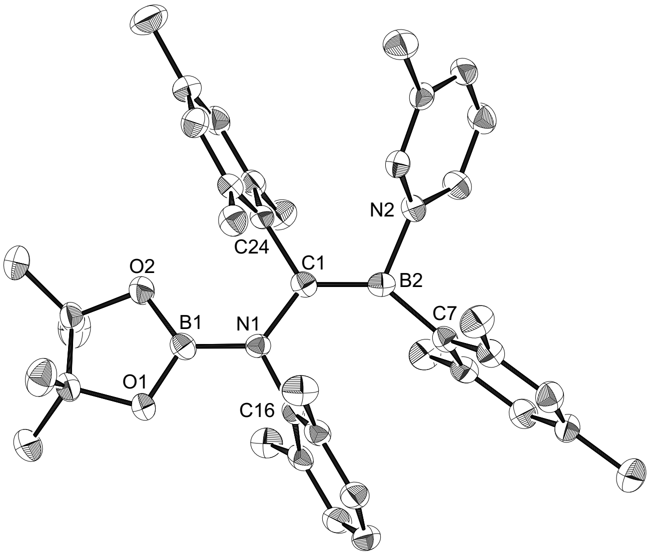

Figure S49. Molecular structure of **4b**

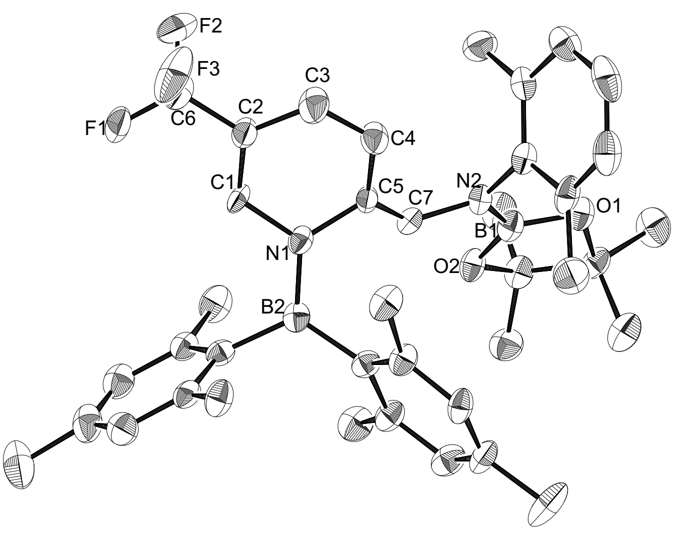

Figure S50. Molecular structure of **5a**

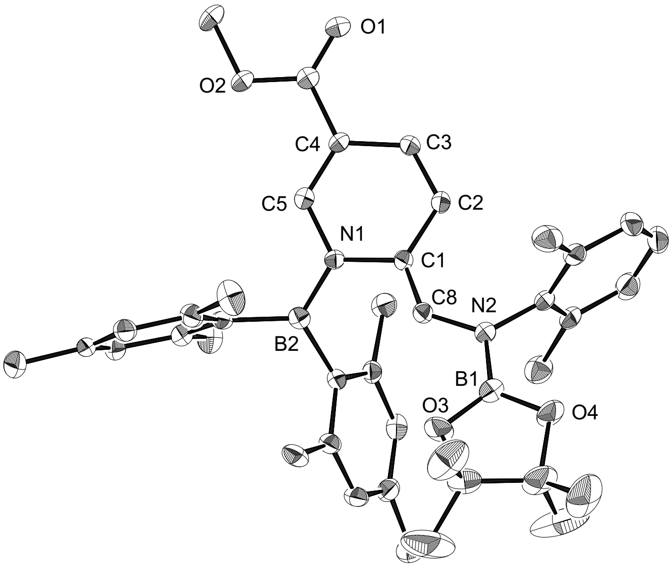

Figure S51. Molecular structure of **5b**

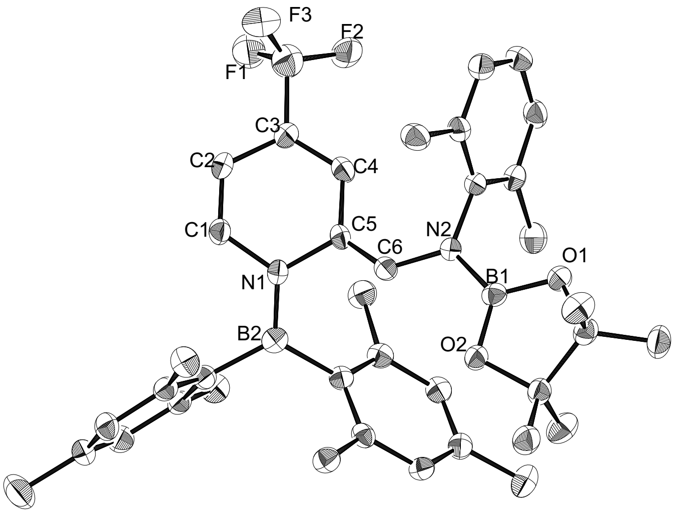

Figure S52. Molecular structure of **5c**

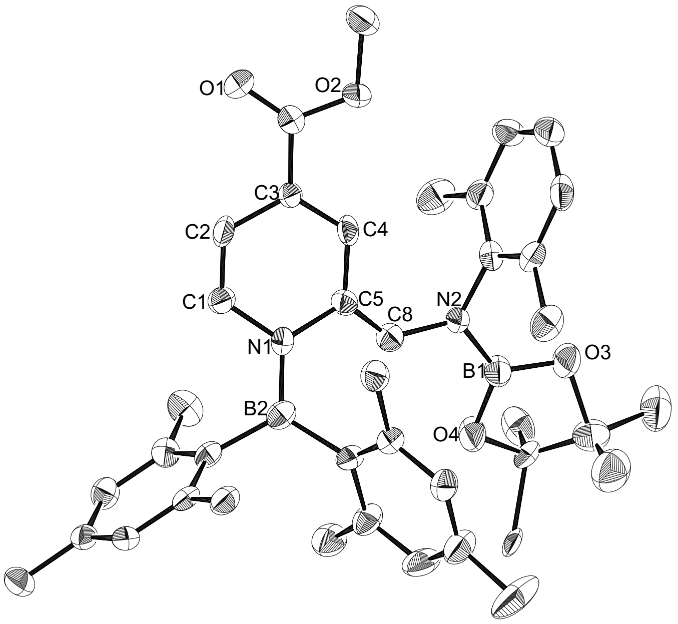

Figure S53. Molecular structure of **5d**

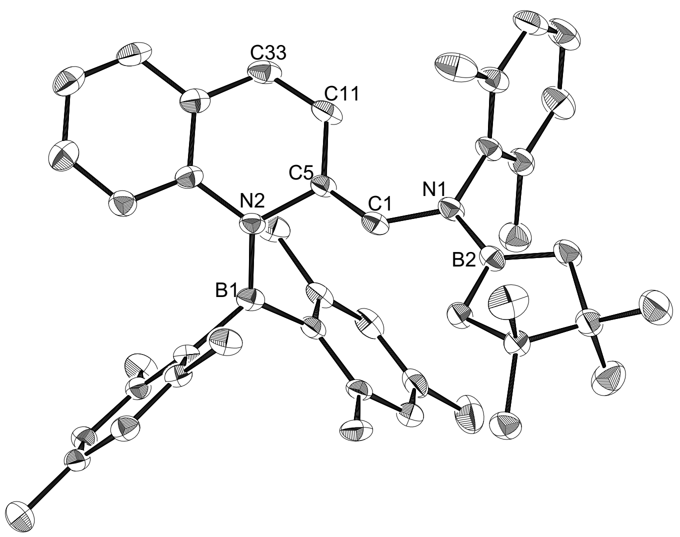

Figure S54. Molecular structure of **5e**

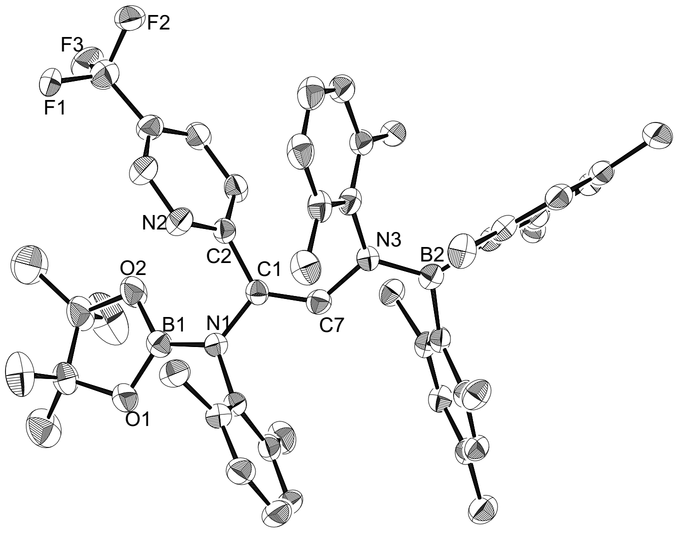

Figure S55. Molecular structure of **9a**

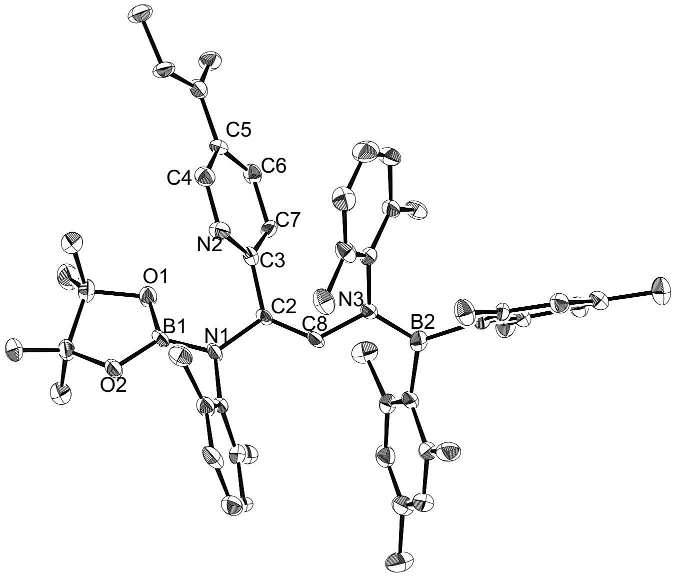

Figure S56. Molecular structure of **9b**

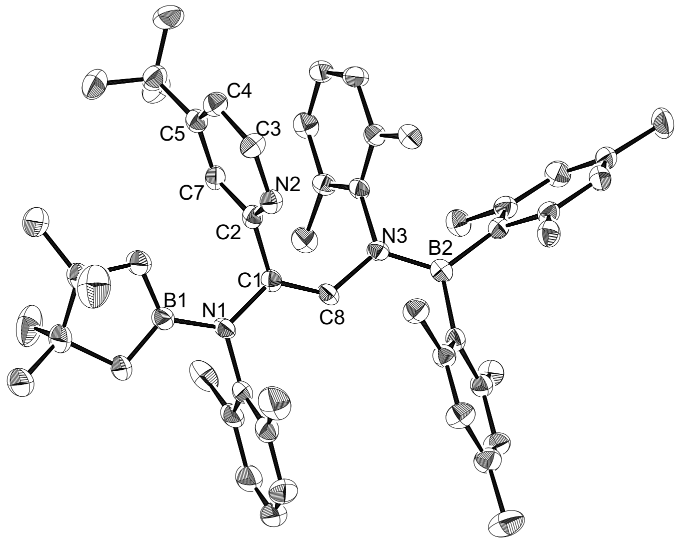

Figure S57. Molecular structure of **9c**

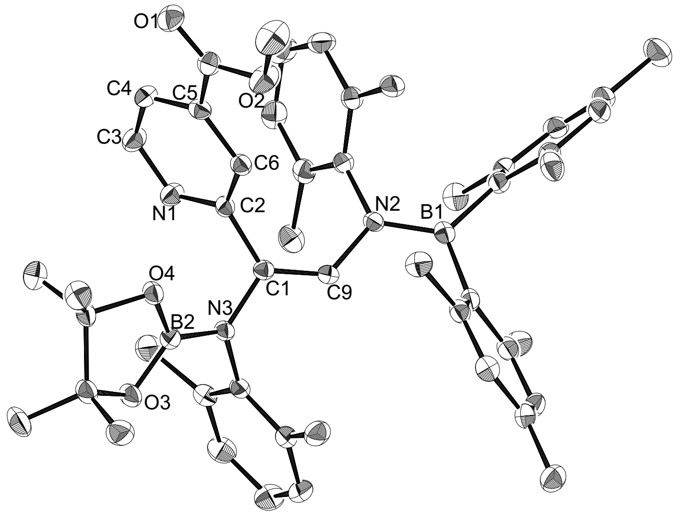

Figure S58. Molecular structure of **9d**

Computational details

Gaussian 16 (rev. A.03) software package was employed to perform all of the calculations.[6] The full model of **4**, **7**, and **8** were calculated by geometry optimization at the B3LYP[7]/6-31+g(d)[8] level of theory in the presence of solvent *n*-hexane using the SCIPCM solvation method.[9] The optimized geometries of **4**, **7**, and **8** are available as Supporting Information in .xyz format. At the optimized structure, TD-DFT[10] calculations were performed to estimate UV-vis spectrum of **4** with CAM-B3LYP[11]/6-31+g(d)[8] level of theory.

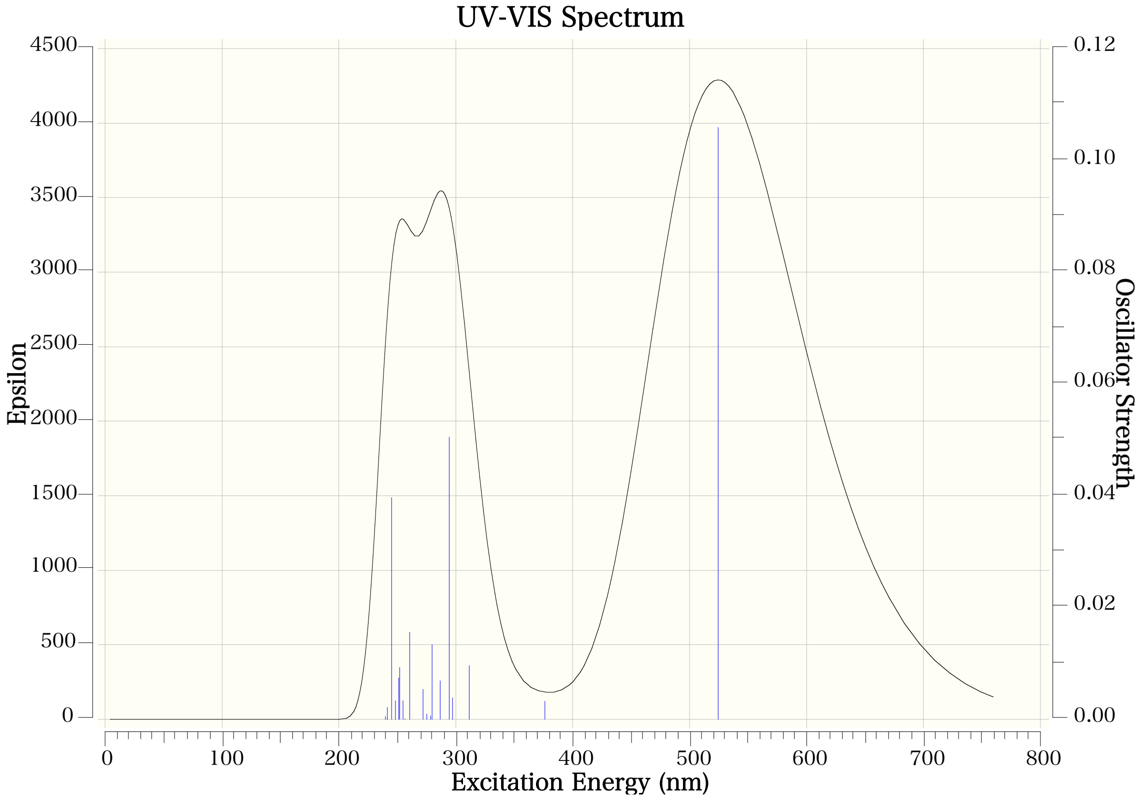

Figure S59. Simulated UV-vis spectrum of **4**

Table S2. Results of TD-DFT calculations of **4**

Excitation energies and oscillator strengths:

Excited State 1:		Singlet-A	
2.3641 eV	524.44 nm	f=0.1059	<S**2>=0.000
	158 ->159	0.69868	
Excited State 2:		Singlet-A	
3.2969 eV	376.07 nm	f=0.0032	<S**2>=0.000
	158 ->160	0.69883	
Excited State 3:		Singlet-A	
3.9800 eV	311.52 nm	f=0.0096	<S**2>=0.000
	154 ->159	0.14784	
	155 ->159	-0.45839	
	156 ->159	0.49231	

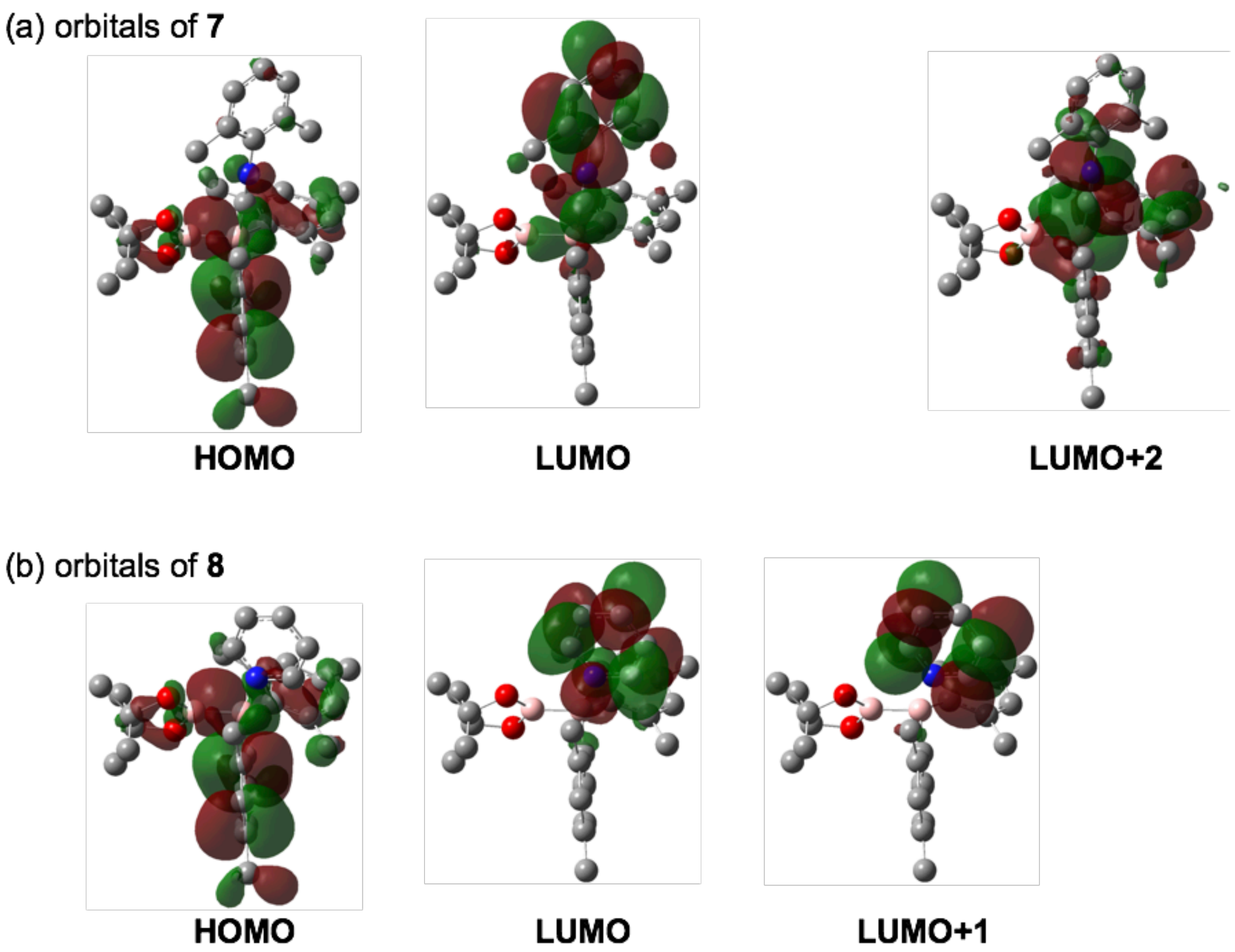

Figure S60. Frontier orbitals and related orbitals of **7** and **8**.

References

S1. H. Asakawa, K.-H. Lee, Z. Lin and M. Yamashita, *Nat. Commun.*, 2014, **5**, 4245.

2. Z. Huang, K. Song, F. Liu, J. Long, H. Hu, H. Gao and Q. Wu, *J. Polym. Sci. A, Polym. Chem.*, 2008, **46**, 1618-1628.

3. (a) C. Kabuto, S. Akine, T. Nemoto and E. Kwon, *J. Cryst. Soc. Jpn.*, 2009, **51**, 218-224; (b) C. Kabuto, S. Akine and E. Kwon, *J. Cryst. Soc. Jpn.*, 2009, **51**, 218-224.

4. M. C. Burla, R. Caliandro, B. Carrozzini, G. L. Cascarano, C. Cuocci, C. Giacovazzo, M. Mallamo, A. Mazzone and G. Polidori, *J. Appl. Crystallogr.*, 2015, **48**, 306-309.

5. G. Sheldrick, *Act. Cryst. Sec. C*, 2015, **71**, 3-8.

6. M. J. Frisch, G. W. Trucks, H. B. Schlegel, G. E. Scuseria, M. A. Robb, J. R. Cheeseman, G. Scalmani, V. Barone, G. A. Petersson, H. Nakatsuji, X. Li, M. Caricato, A. V. Marenich, J. Bloino, B. G. Janesko, R. Gomperts, B. Mennucci, H. P. Hratchian, J. V. Ortiz, A. F. Izmaylov, J. L. Sonnenberg, Williams, F. Ding, F. Lipparini, F. Egidi, J. Goings, B. Peng, A. Petrone, T. Henderson, D. Ranasinghe, V. G. Zakrzewski, J. Gao, N. Rega, G. Zheng, W. Liang, M. Hada, M. Ehara, K. Toyota, R. Fukuda, J. Hasegawa, M. Ishida, T. Nakajima, Y. Honda, O. Kitao, H. Nakai, T. Vreven, K. Throssell, J. A. Montgomery Jr., J. E. Peralta, F. Ogliaro, M. J. Bearpark, J. J. Heyd, E. N. Brothers, K. N. Kudin, V. N. Staroverov, T. A. Keith, R. Kobayashi, J. Normand, K. Raghavachari, A. P. Rendell, J. C. Burant, S. S. Iyengar, J. Tomasi, M. Cossi, J. M. Millam, M. Klene, C. Adamo, R. Cammi, J. W. Ochterski, R. L. Martin, K. Morokuma, O. Farkas, J. B. Foresman and D. J. Fox, *Journal*, 2016.

7. (a) C. Lee, W. Yang and R. G. Parr, *Phys. Rev. B*, 1988, **37**, 785-789; (b) A. D. Becke, *Phys. Rev. A*, 1988, **38**, 3098-3100; (c) B. Miehlich, A. Savin, H. Stoll and H. Preuss, *Chem. Phys. Lett.*, 1989, **157**, 200-206.

8. S. Huzinaga, J. Andzelm, M. Klobukowski, E. Radzio-Andzelm, Y. Sakai and H. Tatewaki, *Gaussian basis sets for molecular calculations*, Elsevier, 1984.

9. (a) J. B. Foresman, T. A. Keith, K. B. Wiberg, J. Snoonian and M. J. Frisch, *J. Phys. Chem.*, 1996, **100**, 16098-16104; (b) J. Tomasi, B. Mennucci and R. Cammi, *Chem. Rev.*, 2005, **105**, 2999-3094.

10. (a) C. Adamo and D. Jacquemin, *Chem. Soc. Rev.*, 2013, **42**, 845-856; (b) A. D. Laurent, C. Adamo and D. Jacquemin, *Phys. Chem. Chem. Phys.*, 2014, **16**, 14334-14356.

11. T. Yanai, D. P. Tew and N. C. Handy, *Chem. Phys. Lett.*, 2004, **393**, 51-57.
